# Supplementary material for: Identifying the best PCR enzyme for library amplification in NGS
Source: Microb Genom. 2024 Apr 5;10(4):001228. doi: 10.1099/mgen.0.001228 (PMC11092157; doi:10.1099/mgen.0.001228)
Supplement: Uncited Supplementary Material 1. [file mgen-10-01228-s001.pdf]

Identification of optimal library PCR enzymes

Quail et al.,

Supplementary Figures

Supplementary Figure 1: Library yield (ng/ul) obtained after 14 cycles of amplification with 1ng of each test microbial genome; BP: *Bordetella pertussis*, EC: *Escherichia coli,* CD: *Clostridioides difficile*, and PF: *Plasmodium falciparum. For each PF was also amplified using a 94C denaturation temperature, "PF 94C".*

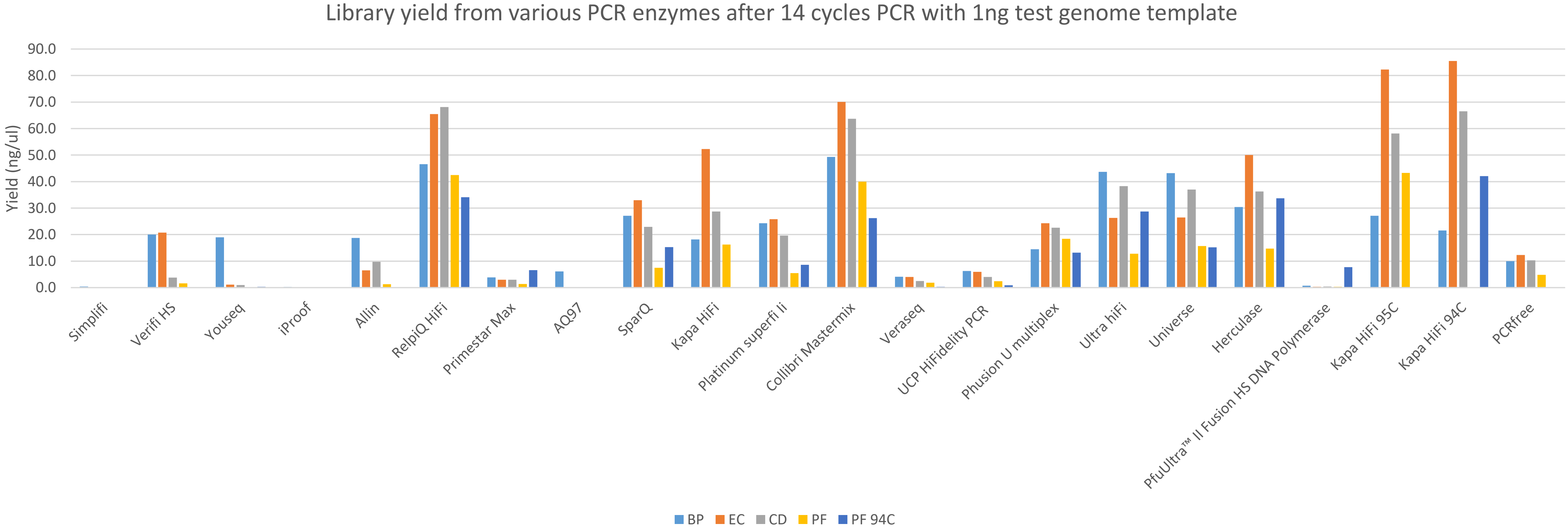

Supplementary Figure 2. Library yield (ng/ul) obtained after 14 cycles of amplification with 1ng of each test microbial genome; Top: *Bordetella pertussis*, Bottom: *E. coli*

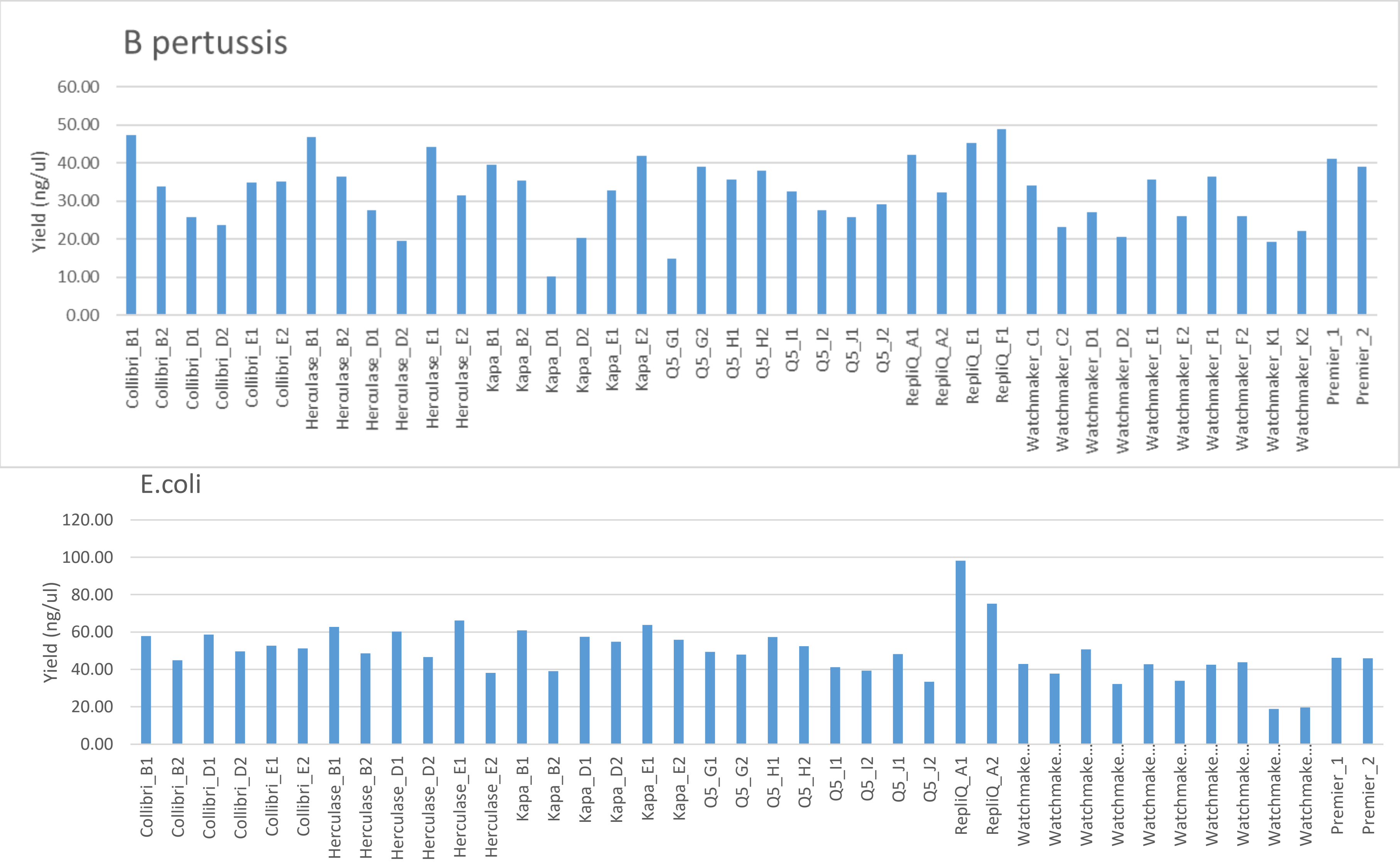

Supplementary Figure 3. Library yield (ng/ul) obtained after 14 cycles of amplification with 1ng of test microbial genome; Top: *C difficile*, Bottom: *P falciparum*

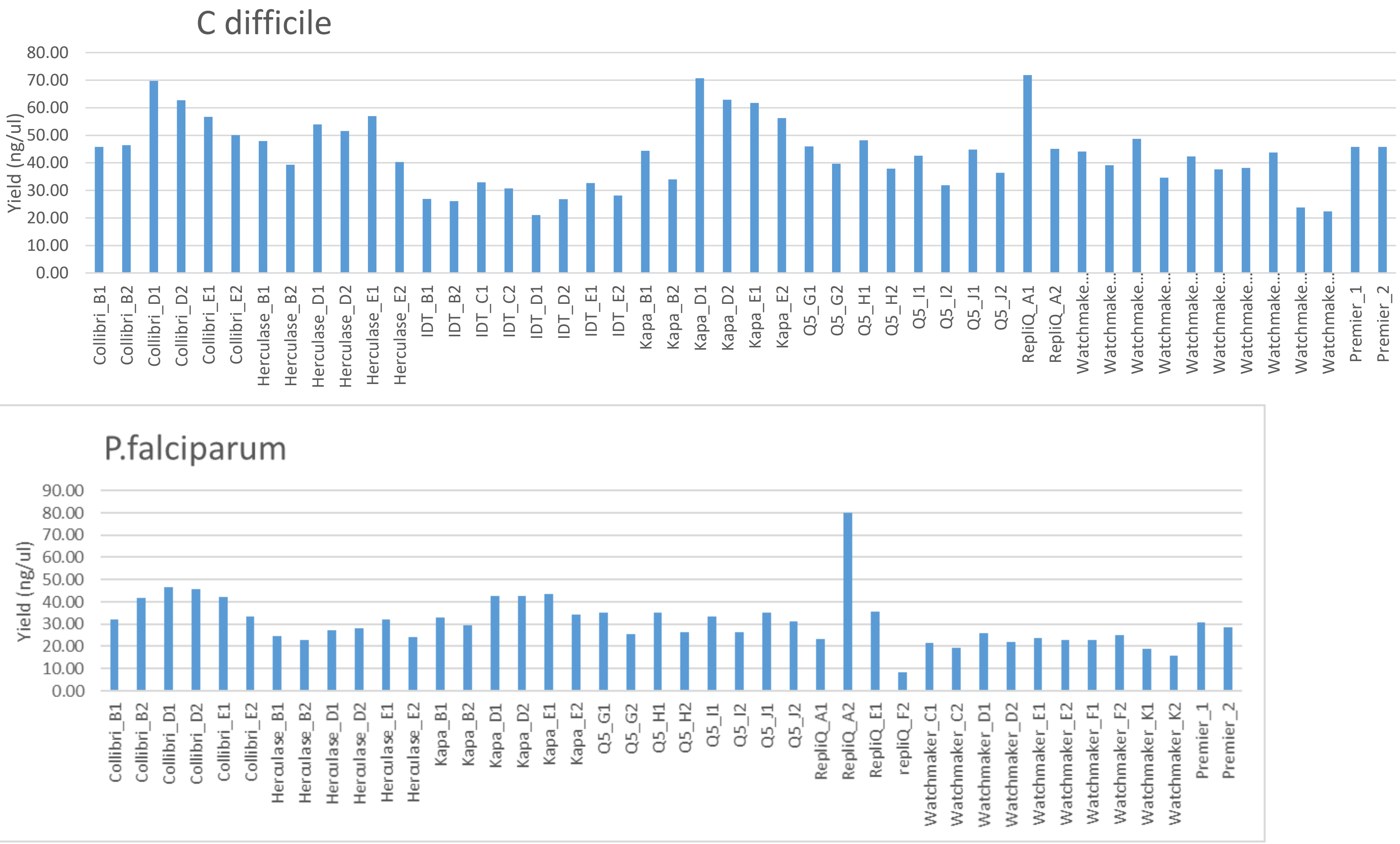

Supplementary Figure 4. Low coverage index value for Human NA12878 sequence 25x coverage datasets after amplification with various enzymes.

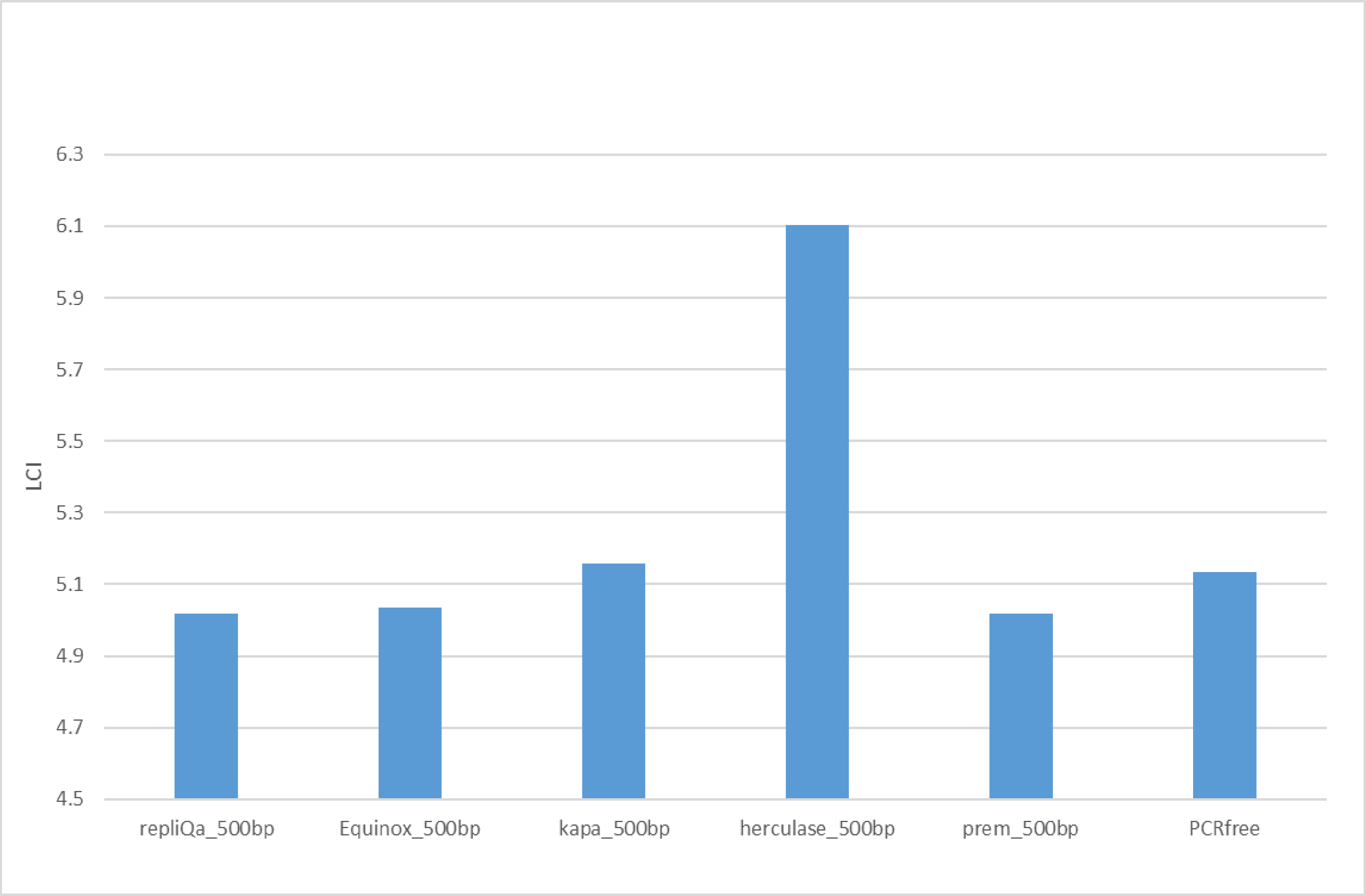

Supplementary Figure 5. Library yields after 14 cycles PCR with 1ng of E.coli Illumina adapter ligated template either without beads present (blue), in the presence of ampure beads (orange) or streptavidin beads (grey).

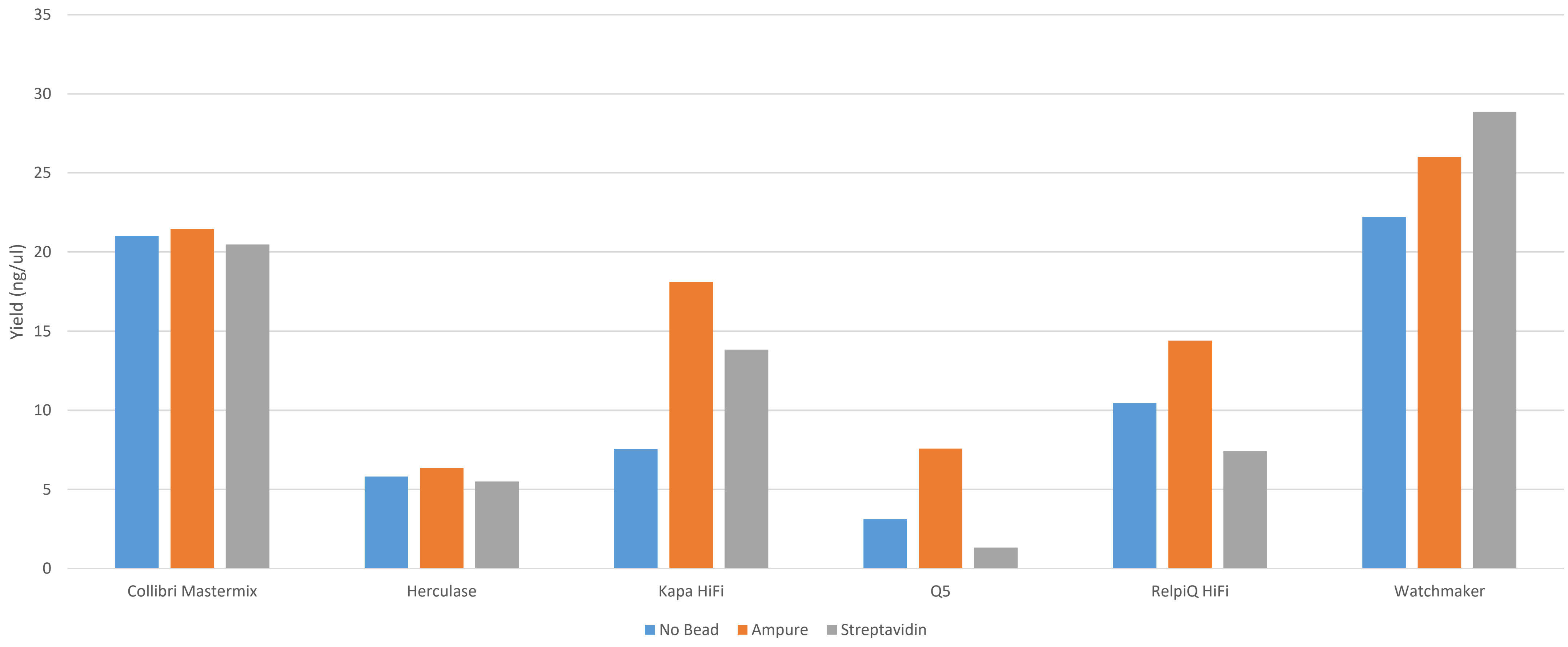

Supplementary Figure 6. Library yields after 14 cycles PCR with 1ng of B. pertussis Illumina adapter ligated template with extension for 5 seconds (blue), 15 seconds (orange), 30 seconds (grey) and 60 seconds (yellow).

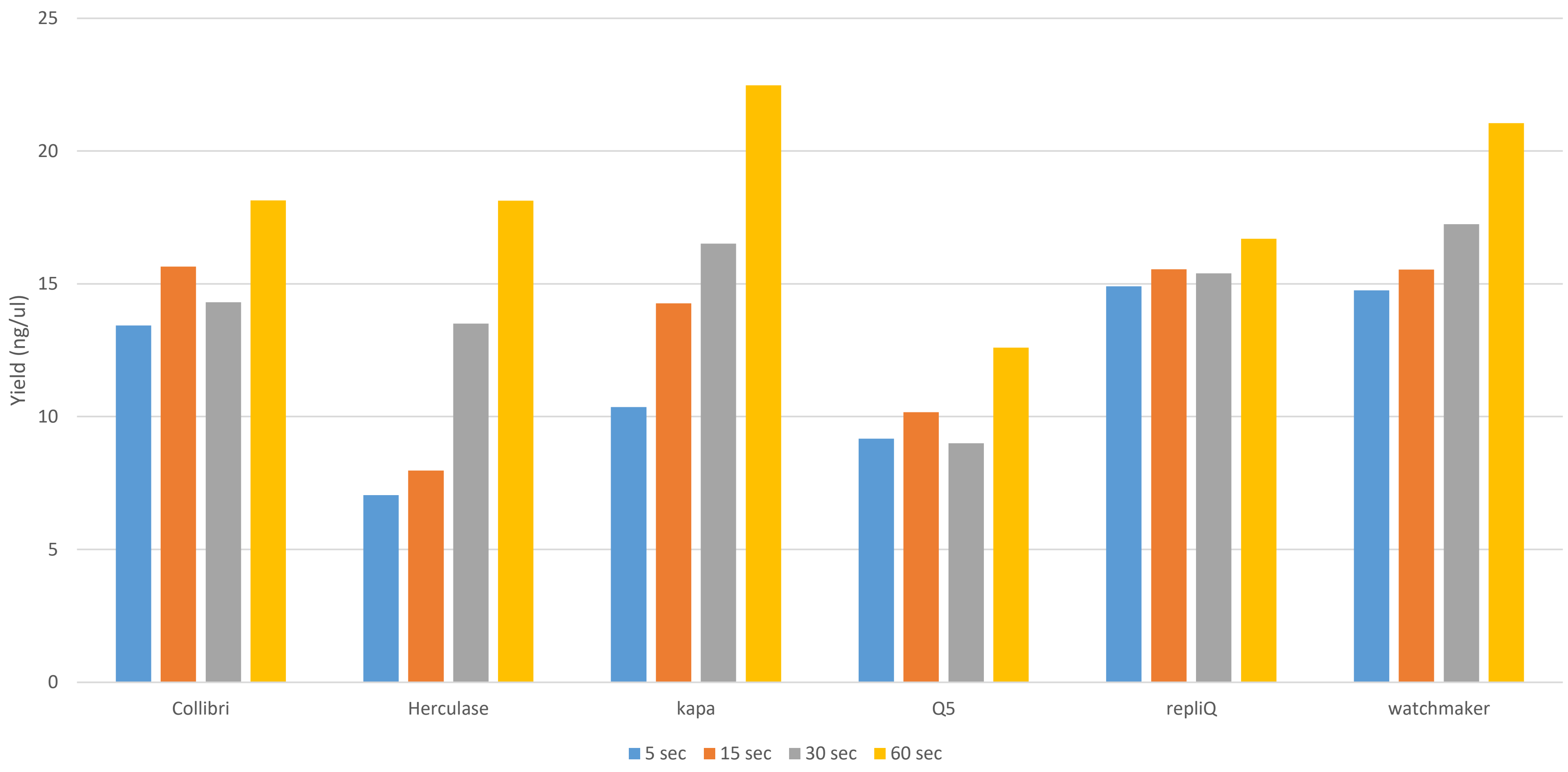

Supplementary Figure 7. Femtopulse electropherograms of size fractionated yeast DNA from a) ELF and b) Blue pippin.

Sample: mq1_yeast_RAD_ELF1
Well Location: F3
Created: 2/11/2022 9:55 AM

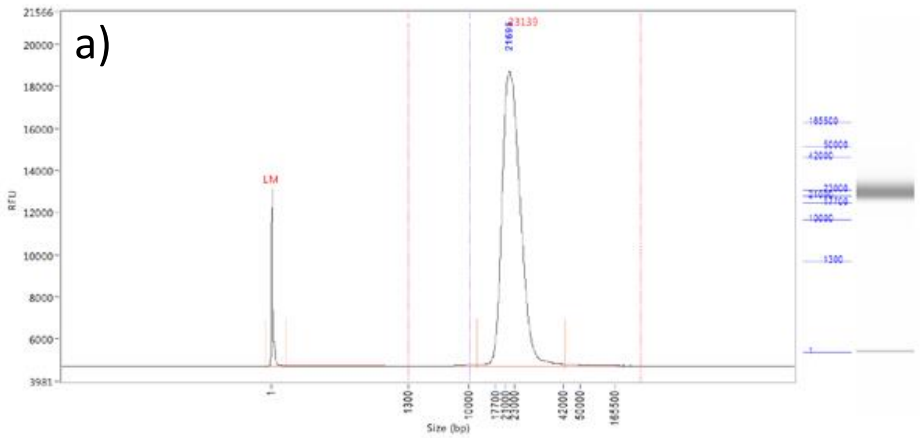

Peak	Size (bp)	Conc. (pg/uL)	From (bp)	To (bp)	Avg. Size (bp)	CV%	RFU	Corr. Peak Area
1	1 (LM)	2.0831	0	141	4	298.54	8399	64.374
2	21695	214.1000	12275	42826	22749	13.65	14046	661.652

TIC:	214.1000	pg/uL
TIM:	16.2475	pmole/L
Total Conc.:	216.5718	pg/uL

Sample: Yeast #2_12Kb_RAD_PrLIBP
Well location: A2
Created: 2/15/2022 2:21 PM

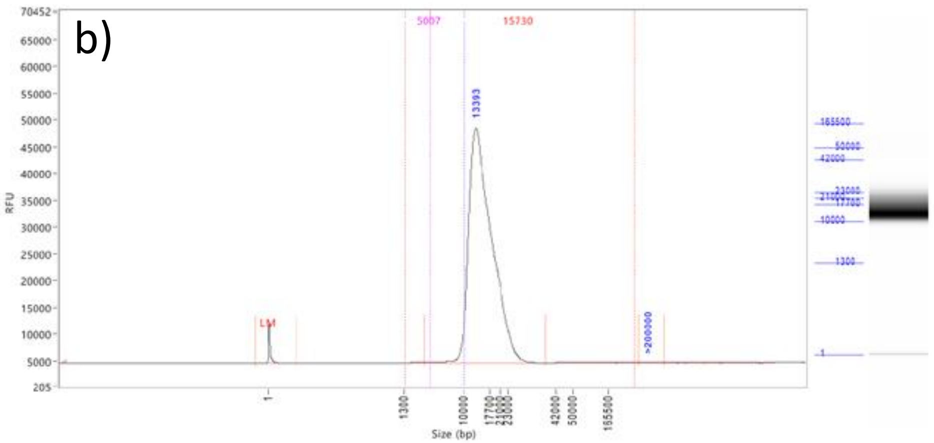

Peak	Size (bp)	Concentration (ng/uL)	Relative concentration Percent	Molarity (nmole/L)	From (bp)	To (bp)	CV%	RFU
1	1 (LM)	0.0025		0.7748	0	267	360.97	7627
2	13393	1.1130	99.9	0.1183	4158	37888	23.46	43880
3	>200000	0.0010	0.1	0.0000	>200000	>200000	5.63	185

Supplementary Figure 8. Agilent Tapestation genomic kit electropherograms from long range PCR with different enzymes after 15 cycles of PCR from Sage Sciences ELF fractionation (A) and Bluepippin (B).
Note. Electropherogram images have been cropped to diaply informative areas

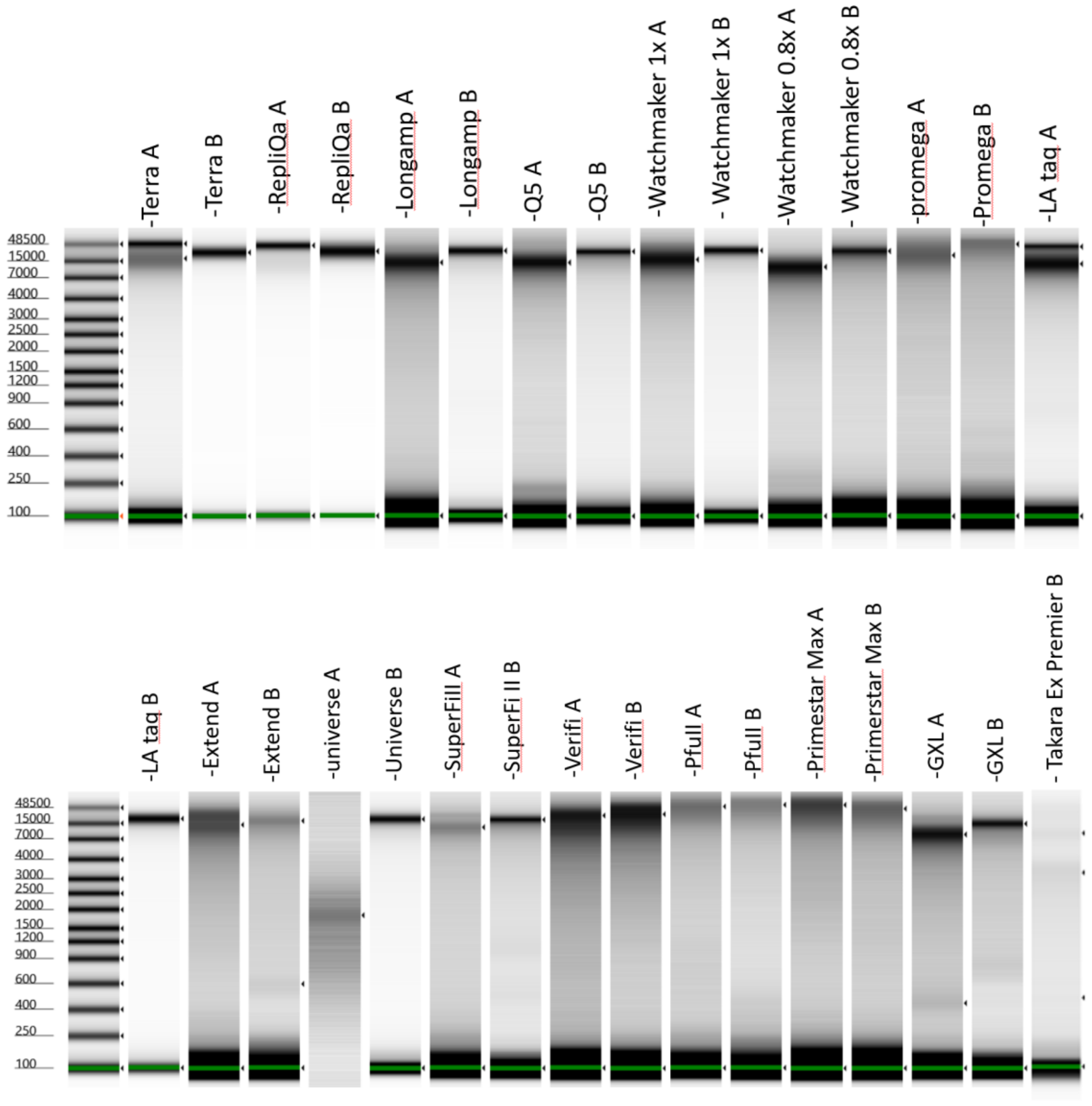

Supplementary Figure 9. PacBio HiFi Sequel IIe sequence yields and coverage obtained from barcoded long template PCR libraries.

Enzyme	Template	PCR cycles	Polymerase Reads	Bases	Estimated Coverage	Sample number	Sample name
Terra	ELF	15	132,515	851,869,210	70.98910083	1	terraELF15
Terra	Bluepippin	15	69,375	811,670,287	67.63919058	2	terrabluepippin15
repliQa	ELF	15	95,403	874,550,481	72.87920675	3	repliqELF15
repliQa	Bluepippin	15	67,874	851,073,366	70.9227805	4	repliqbluepippin15
LongAmp	ELF	15	52,592	256,665,751	21.38881258	5	longampELF15
LongAmp	Bluepippin	15	69,634	626,495,361	52.20794675	6	longamp bluepippin15
Q5	ELF	15	56,635	248,826,686	20.73555717	7	Q5ELF15
Q5	Bluepippin	15	76,603	523,297,797	43.60814975	8	Q5 bluepippin15
Watchmaker Equinox	ELF	15	89,058	419,979,074	34.99825617	9	WmELF15
Watchmaker Equinox	Bluepippin	15	132,438	1,120,679,413	93.38995108	10	Wmbluepippin15
Watchmaker Equinox	ELF	15	63,812	298,235,529	24.85296075	11	WmELF15
Watchmaker Equinox	Bluepippin	15	15,249	119,965,903	9.997158583	12	Wmbluepippin15
Promega Go Taq Long	ELF	15	7,132	35,752,833	2.97940275	13	promegaELF15
Promega Go Taq Long	Bluepippin	15	11	25,388	0.002115667	14	promegabluepippin15
SuperFi II	ELF	15	96,073	536,211,718	44.68430983	15	superfiELF15
SuperFi II	Bluepippin	15	85,578	903,200,782	75.26673183	16	superfibluepippin15
Kapa HiFi	ELF	15	26,974	94,586,892	7.882241	17	kapaELF15
Kapa HiFi	Bluepippin	15	11,265	42,909,095	3.575757917	18	kapabluepippin15
Universe	ELF	15	119,121	599,671,617	49.97263475	19	universeELF15
Universe	Bluepippin	15	51,436	470,686,450	39.22387083	20	universebluepippin15
LaTaq	ELF	15	10,408	53,704,734	4.4753945	21	LaTaqELF15
LaTaq	Bluepippin	15	17,896	163,824,105	13.65200875	22	LaTaq bluepippin15
Expand	ELF	15	12,591	43,700,917	3.641743083	23	ExpandELF15
Expand	Bluepippin	15	10,245	41,390,920	3.449243333	24	Expandbluepippin15
Verifi	ELF	15	20	116,561	0.009713417	25	verifiELF15
Verifi	Bluepippin	15	38	361,609	0.030134083	26	verifibluepippin15
Pfu Ultra II	ELF	15	4	16,767	0.00139725	27	PfuultraIIELF15
Pfu Ultra II	Bluepippin	15	5	18,351	0.00152925	28	PfuultraIIbluepippin15
Primestar Max	ELF	15	28,367	117,920,076	9.826673	29	PrimestarMAXELF15
Primestar Max	Bluepippin	15	28,306	173,936,027	14.49466892	30	PrimestarMAXbluepippin15
Primestar GXL	ELF	15	13	45,198	0.0037665	31	PrimestarGXLELF15
Primestar GXL	Bluepippin	15	20	70,261	0.005855083	32	PrimestarGXLbluepippin15
Terra	ELF	12	20,612	135,188,167	11.26568058	33	TerraF1_12
Terra	Bluepippin	12	24,485	277,871,931	23.15599425	34	TerraB_12
repliQa	ELF	12	32,557	317,548,565	26.46238042	35	repliQaF1_12
repliQa	Bluepippin	12	39,211	508,550,579	42.37921492	36	repliQaB_12
LongAmp	ELF	12	7,294	38,968,104	3.247342	37	LongAmpF1_12
LongAmp	Bluepippin	12	20,402	196,885,031	16.40708592	38	LongAmpB_12
Promega Go Taq Long	ELF	12	11,082	71,125,825	5.927152083	39	promegaF2_12
Promega Go Taq Long	Bluepippin	12	52,834	591,119,208	49.259934	40	promegaB_12

Supplementary Figure 10. Low coverage index for sequencing *S. cerevisiae* 20kb fragments at 30x depth on PacBio Sequel IIe.

Bars are labelled according to enzyme used, method of size selection (A denotes ELF fractionation, B Bluepippin) and PCR cycles used (X12 samples had 12 cycles PCR, x15 had 15 cycles).

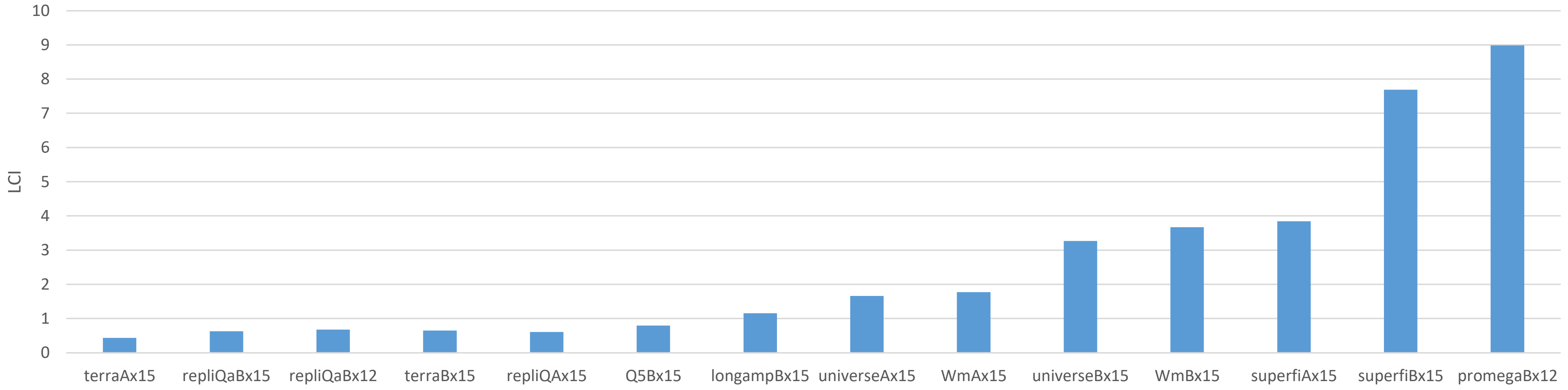

Supplementary Figure 11. Average subread lengths, ranked by size, after PacBio HiFi sequencing for amplicon libraries that gave >30x genome coverage.

Libraries have fragments with either ELF fractionation or Bluepippin as indicated. “12” samples had 12 cycles PCR, “15” had 15 cycles.

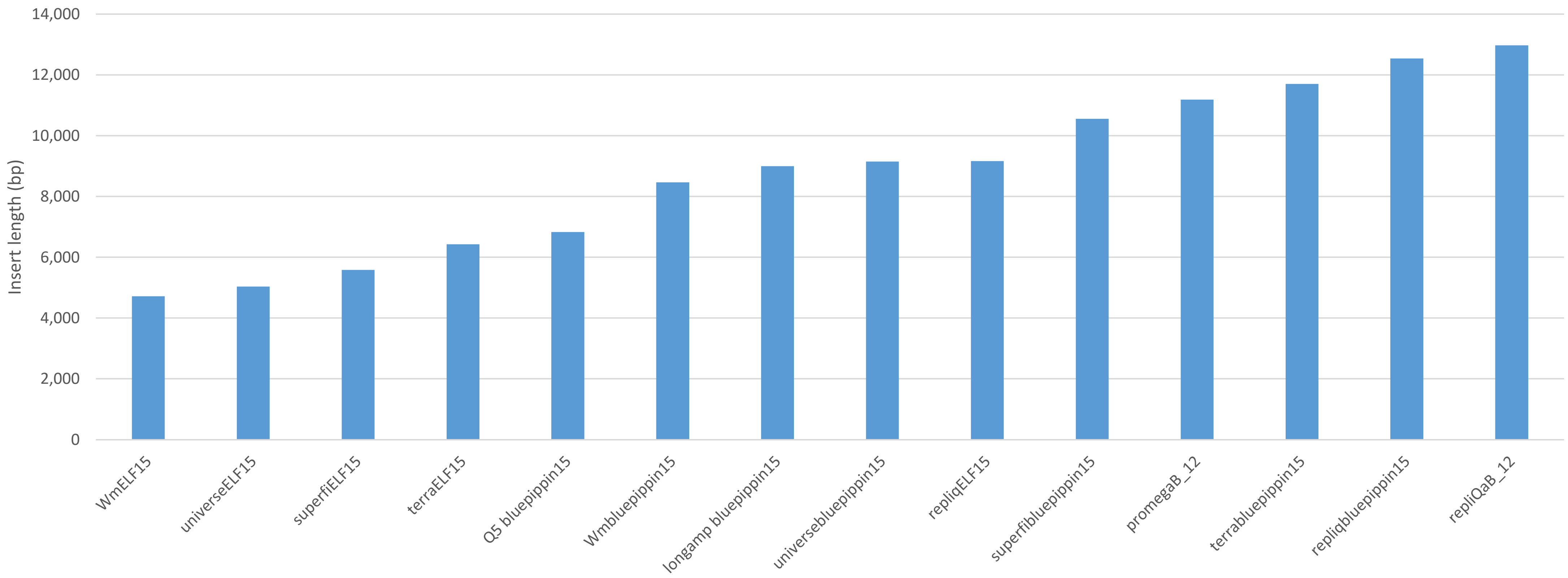

Supplementary Figure 12. PacBio HiFi read length heatmap plot from SMRTlink interface for each library sequenced.

Bars are labelled according to enzyme used, method of size selection (A denotes ELF fractionation, B Bluepippin) and PCR cycles used (X12 samples had 12 cycles PCR, x15 had 15 cycles).

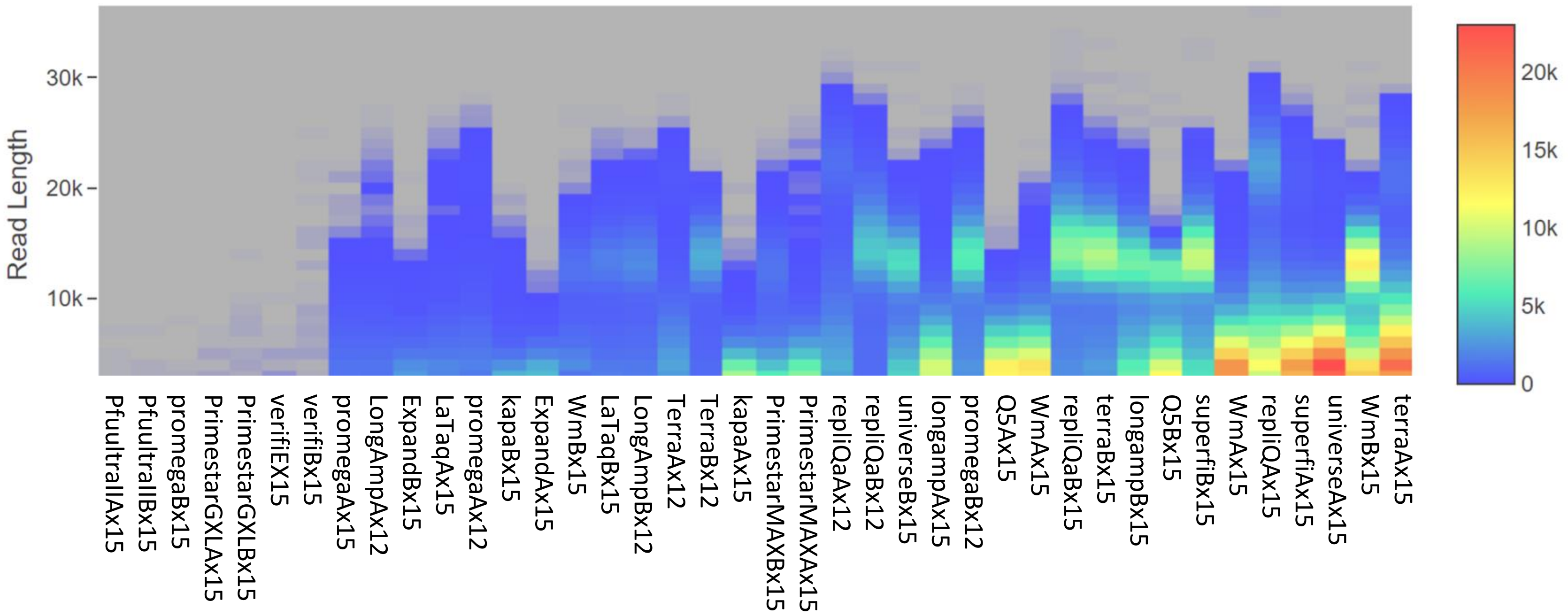

Supplementary Figure 13. HiFi subread length distributions for ELF size fractionation of a) Terra polymerase and b) repliQa amplified fragments and Bluepippin fractionation of c) Terra polymerase and d) repliQa amplified fragments, after 15 cycles PCR

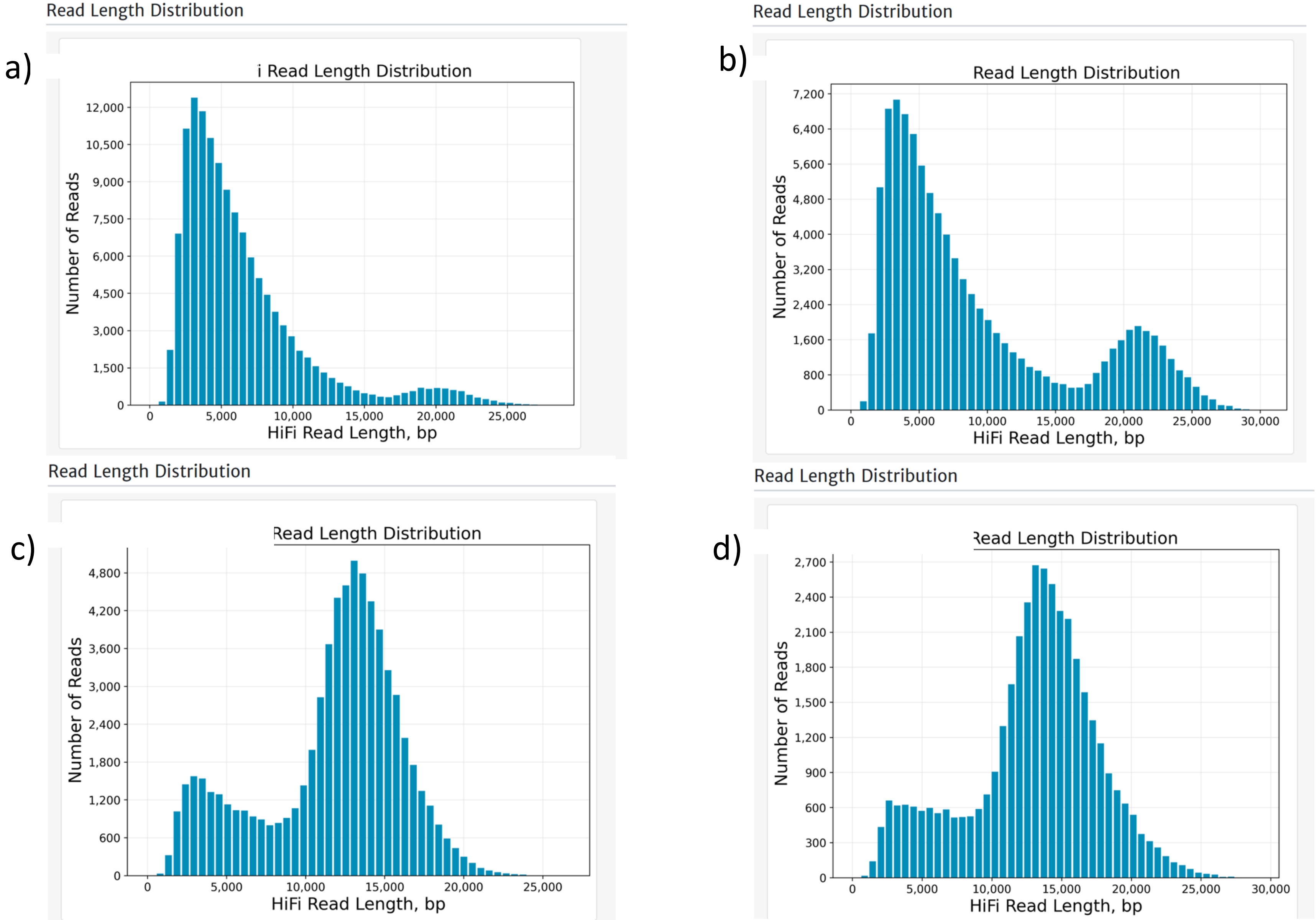

Supplementary Figure 14. Percentage mismatch, insertion and deletion errors in PacBio HiFi reads with datasets that gave >30x genome coverage ranked from lowest to highest rate of mismatch error.
A denotes ELF fractionation, B Bluepippin. “**12**” samples had 12 cycles PCR, **“15”** had 15 cycles.

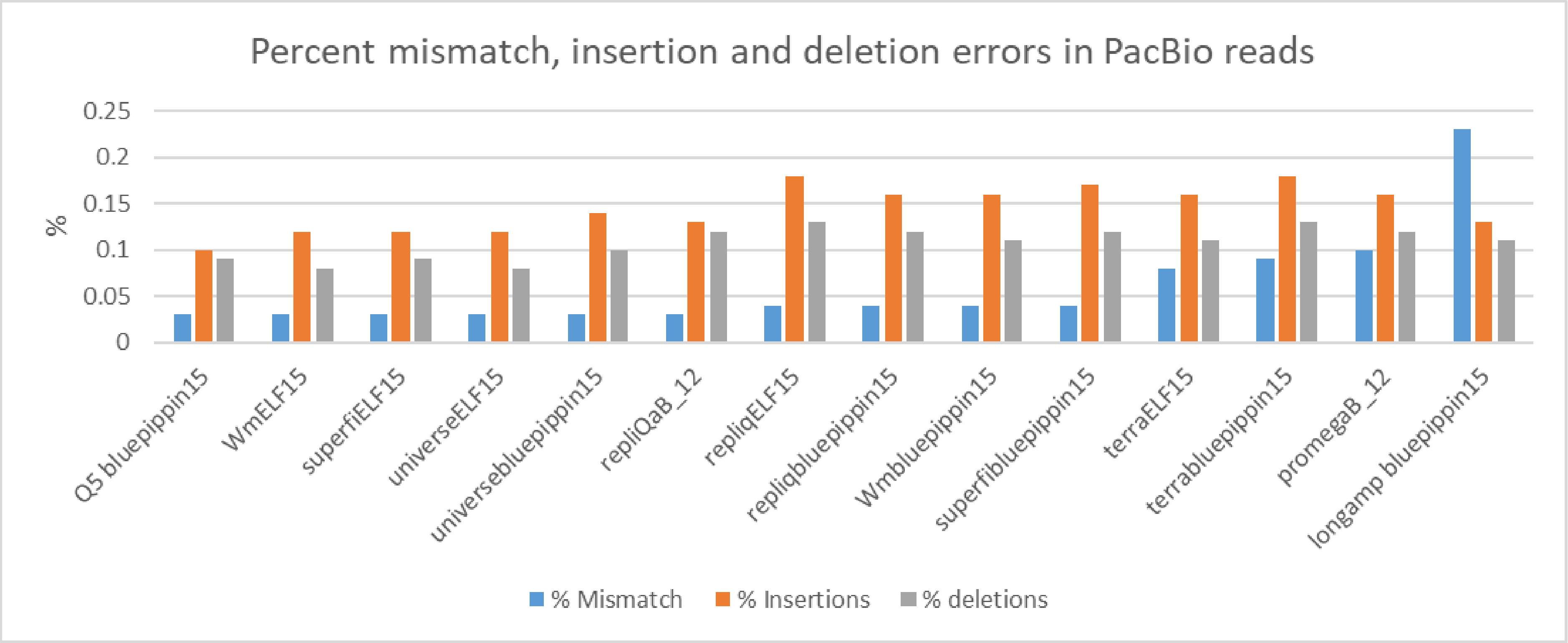

Supplementary Table 1:

Sequences of unique dual indexing oligonucleotide primers and Illumina sequencing adapter used in this study

TSQ Adapter - order hplc pure

Top_ TSQ_strand
A*CACTCTTTCCCTACACGACGCTCTTCCGATC*T

Bottom_TSQ_strand
/5PHOS/G*ATCGGAAGAGCACACGTCTGAACTCCAGTCA*C

*=phosphophioate to protect against nucleases.

i7 and i5 UDI Indexing PCR primers

i5 Illumina PCR primer is
AATGATACGGCGACCACCGAGATCTACAC[barcode from below]ACACTCTTTCCCTACACGACGCTCTTCCGATC*T

i7 Illumina PCR primer is
CAAGCAGAAGACGGCATACGAGAT[barcode]GTGACTGGAGTTCAGACGTGTGCTCTTCCGATC*T

Ordered as Truegrade desalted oligos.

SetA

i5 barcodes | i7 barcodes

	Barcode sequence in oligo				Sequence Obtained	Barcode sequence in oligo		i5 oligo to order		i7 oligo to order
D5_063	ATGCGACT	1		D7_063	CGGAGACA	TGTCTCCG		AATGATACGGCGACCACCGAGATCTACACATGCGACTACACTCTTTCCCTACACGACGCTCTTCCGATC*T		CAAGCAGAAGACGGCATACGAGATTGTCTCCGGTGACTGGAGTTCAGACGTGTGCTCTTCCGATC*T
D5_095	TCACAAAC	2		D7_095	GTTAACGT	ACGTTAAC		AATGATACGGCGACCACCGAGATCTACACTCACAAACACACTCTTTCCCTACACGACGCTCTTCCGATC*T		CAAGCAGAAGACGGCATACGAGATACGTTAACGTGACTGGAGTTCAGACGTGTGCTCTTCCGATC*T
D5_387	AGGAGAAA	3		D7_387	CATTTATT	AATAAATG		AATGATACGGCGACCACCGAGATCTACACAGGAGAAAACACTCTTTCCCTACACGACGCTCTTCCGATC*T		CAAGCAGAAGACGGCATACGAGATAATAAATGGTGACTGGAGTTCAGACGTGTGCTCTTCCGATC*T
D5_388	TCGGCAAA	4		D7_388	TTAGCGCA	TGCGCTAA		AATGATACGGCGACCACCGAGATCTACACTCGGCAAAACACTCTTTCCCTACACGACGCTCTTCCGATC*T		CAAGCAGAAGACGGCATACGAGATTGCGCTAAGTGACTGGAGTTCAGACGTGTGCTCTTCCGATC*T
D5_389	AGAAGCCC	5		D7_389	TTGATTCC	GGAATCAA		AATGATACGGCGACCACCGAGATCTACACAGAAGCCCACACTCTTTCCCTACACGACGCTCTTCCGATC*T		CAAGCAGAAGACGGCATACGAGATGGAATCAAGTGACTGGAGTTCAGACGTGTGCTCTTCCGATC*T
D5_390	CTGAAAGA	6		D7_390	TATTCGTA	TACGAATA		AATGATACGGCGACCACCGAGATCTACACCTGAAAGAACACTCTTTCCCTACACGACGCTCTTCCGATC*T		CAAGCAGAAGACGGCATACGAGATTACGAATAGTGACTGGAGTTCAGACGTGTGCTCTTCCGATC*T
D5_391	AGGCCCGG	7		D7_391	TTACGCCG	CGGCGTAA		AATGATACGGCGACCACCGAGATCTACACAGGCCCGGACACTCTTTCCCTACACGACGCTCTTCCGATC*T		CAAGCAGAAGACGGCATACGAGATCGGCGTAAGTGACTGGAGTTCAGACGTGTGCTCTTCCGATC*T
D5_392	AGTTTCTC	8		D7_392	GTGATCAG	CTGATCAC		AATGATACGGCGACCACCGAGATCTACACAGTTTCTCACACTCTTTCCCTACACGACGCTCTTCCGATC*T		CAAGCAGAAGACGGCATACGAGATCTGATCACGTGACTGGAGTTCAGACGTGTGCTCTTCCGATC*T
D5_393	AAGTGTCG	9		D7_393	TATATACG	CGTATATA		AATGATACGGCGACCACCGAGATCTACACAAGTGTCGACACTCTTTCCCTACACGACGCTCTTCCGATC*T		CAAGCAGAAGACGGCATACGAGATCGTATATAGTGACTGGAGTTCAGACGTGTGCTCTTCCGATC*T
D5_394	GAACAAGG	10		D7_394	TTATCCGT	ACGGATAA		AATGATACGGCGACCACCGAGATCTACACGAACAAGGACACTCTTTCCCTACACGACGCTCTTCCGATC*T		CAAGCAGAAGACGGCATACGAGATACGGATAAGTGACTGGAGTTCAGACGTGTGCTCTTCCGATC*T
D5_395	ACAATGTT	11		D7_395	GTACCAAA	TTTGGTAC		AATGATACGGCGACCACCGAGATCTACACACAATGTTACACTCTTTCCCTACACGACGCTCTTCCGATC*T		CAAGCAGAAGACGGCATACGAGATTTTGGTACGTGACTGGAGTTCAGACGTGTGCTCTTCCGATC*T
D5_396	CGCGCCGG	12		D7_396	CAGAGGGA	TCCCTCTG		AATGATACGGCGACCACCGAGATCTACACCGCGCCGGACACTCTTTCCCTACACGACGCTCTTCCGATC*T		CAAGCAGAAGACGGCATACGAGATTCCCTCTGGTGACTGGAGTTCAGACGTGTGCTCTTCCGATC*T
D5_397	ACCTAACT	13		D7_397	CATCATTT	AAATGATG		AATGATACGGCGACCACCGAGATCTACACACCTAACTACACTCTTTCCCTACACGACGCTCTTCCGATC*T		CAAGCAGAAGACGGCATACGAGATAAATGATGGTGACTGGAGTTCAGACGTGTGCTCTTCCGATC*T
D5_398	CATAAGTC	14		D7_398	GTTCCGAT	ATCGGAAC		AATGATACGGCGACCACCGAGATCTACACCATAAGTCACACTCTTTCCCTACACGACGCTCTTCCGATC*T		CAAGCAGAAGACGGCATACGAGATATCGGAACGTGACTGGAGTTCAGACGTGTGCTCTTCCGATC*T
D5_399	CACAACAC	15		D7_399	TAGGCGCG	CGCGCCTA		AATGATACGGCGACCACCGAGATCTACACCACAACACACACTCTTTCCCTACACGACGCTCTTCCGATC*T		CAAGCAGAAGACGGCATACGAGATCGCGCCTAGTGACTGGAGTTCAGACGTGTGCTCTTCCGATC*T
D5_400	ATAATACA	16		D7_400	GACTTATA	TATAAGTC		AATGATACGGCGACCACCGAGATCTACACATAATACAACACTCTTTCCCTACACGACGCTCTTCCGATC*T		CAAGCAGAAGACGGCATACGAGATTATAAGTCGTGACTGGAGTTCAGACGTGTGCTCTTCCGATC*T
D5_502	GTTGCTAT	17		D7_1001	GCATCGAC	GTCGATGC		AATGATACGGCGACCACCGAGATCTACACGTTGCTATACACTCTTTCCCTACACGACGCTCTTCCGATC*T		CAAGCAGAAGACGGCATACGAGATGTCGATGCGTGACTGGAGTTCAGACGTGTGCTCTTCCGATC*T
D5_402	TCCAACGA	18		D7_402	ACTTTGTA	TACAAAGT		AATGATACGGCGACCACCGAGATCTACACTCCAACGAACACTCTTTCCCTACACGACGCTCTTCCGATC*T		CAAGCAGAAGACGGCATACGAGATTACAAAGTGTGACTGGAGTTCAGACGTGTGCTCTTCCGATC*T
D5_403	GGTCGAAA	19		D7_403	CCTAAACT	AGTTTAGG		AATGATACGGCGACCACCGAGATCTACACGGTCGAAAACACTCTTTCCCTACACGACGCTCTTCCGATC*T		CAAGCAGAAGACGGCATACGAGATAGTTTAGGGTGACTGGAGTTCAGACGTGTGCTCTTCCGATC*T
D5_404	TATCGTAA	20		D7_404	GGCTCGTA	TACGAGCC		AATGATACGGCGACCACCGAGATCTACACTATCGTAAACACTCTTTCCCTACACGACGCTCTTCCGATC*T		CAAGCAGAAGACGGCATACGAGATTACGAGCCGTGACTGGAGTTCAGACGTGTGCTCTTCCGATC*T

D5_405	AGATCCCG	21		D7_405	CTCCGACC	GGTCGGAG		AATGATACGGCGACCACCGAGATCTACACAGATCCCGACACTCTTTCCCTACACGACGCTCTTCCGATC*T	CAAGCAGAAGACGGCATACGAGATGGTCGGAGGTGACTGGAGTTCAGACGTGTGCTCTTCCGATC*T
D5_406	TCCAGGAT	22		D7_406	CCGCTAAA	TTTAGCGG		AATGATACGGCGACCACCGAGATCTACACTCCAGGATACACTCTTTCCCTACACGACGCTCTTCCGATC*T	CAAGCAGAAGACGGCATACGAGATTTTAGCGGGTGACTGGAGTTCAGACGTGTGCTCTTCCGATC*T
D5_407	TAACACAA	23		D7_407	TCCACCTT	AAGGTGGA		AATGATACGGCGACCACCGAGATCTACACTAACACAAACACTCTTTCCCTACACGACGCTCTTCCGATC*T	CAAGCAGAAGACGGCATACGAGATAAGGTGGAGTGACTGGAGTTCAGACGTGTGCTCTTCCGATC*T
D5_408	CTGGTAAC	24		D7_408	AACTTCAA	TTGAAGTT		AATGATACGGCGACCACCGAGATCTACACCTGGTAACACACTCTTTCCCTACACGACGCTCTTCCGATC*T	CAAGCAGAAGACGGCATACGAGATTTGAAGTTGTGACTGGAGTTCAGACGTGTGCTCTTCCGATC*T
D5_409	GAAAGGTA	25		D7_409	CTAACTAG	CTAGTTAG		AATGATACGGCGACCACCGAGATCTACACGAAAGGTAACACTCTTTCCCTACACGACGCTCTTCCGATC*T	CAAGCAGAAGACGGCATACGAGATCTAGTTAGGTGACTGGAGTTCAGACGTGTGCTCTTCCGATC*T
D5_410	ACACATAT	26		D7_410	TTTGCAAA	TTTGCAAA		AATGATACGGCGACCACCGAGATCTACACACACATATACACTCTTTCCCTACACGACGCTCTTCCGATC*T	CAAGCAGAAGACGGCATACGAGATTTTGCAAAGTGACTGGAGTTCAGACGTGTGCTCTTCCGATC*T
D5_411	GAACCCTG	27		D7_411	CTTAGAGT	ACTCTAAG		AATGATACGGCGACCACCGAGATCTACACGAACCCTGACACTCTTTCCCTACACGACGCTCTTCCGATC*T	CAAGCAGAAGACGGCATACGAGATACTCTAAGGTGACTGGAGTTCAGACGTGTGCTCTTCCGATC*T
D5_412	TAAACCCA	28		D7_412	GGTGGGAA	TTCCCACC		AATGATACGGCGACCACCGAGATCTACACTAAACCCAACACTCTTTCCCTACACGACGCTCTTCCGATC*T	CAAGCAGAAGACGGCATACGAGATTTCCCACCGTGACTGGAGTTCAGACGTGTGCTCTTCCGATC*T
D5_413	GCTAATTG	29		D7_413	ATATGCGC	GCGCATAT		AATGATACGGCGACCACCGAGATCTACACGCTAATTGACACTCTTTCCCTACACGACGCTCTTCCGATC*T	CAAGCAGAAGACGGCATACGAGATGCGCATATGTGACTGGAGTTCAGACGTGTGCTCTTCCGATC*T
D5_414	CCGTCCCG	30		D7_414	CCACGTAT	ATACGTGG		AATGATACGGCGACCACCGAGATCTACACCCGTCCCGACACTCTTTCCCTACACGACGCTCTTCCGATC*T	CAAGCAGAAGACGGCATACGAGATATACGTGGGTGACTGGAGTTCAGACGTGTGCTCTTCCGATC*T
D5_415	TGGAGTGT	31		D7_415	AGCCTGCA	TGCAGGCT		AATGATACGGCGACCACCGAGATCTACACTGGAGTGTACACTCTTTCCCTACACGACGCTCTTCCGATC*T	CAAGCAGAAGACGGCATACGAGATTGCAGGCTGTGACTGGAGTTCAGACGTGTGCTCTTCCGATC*T
D5_416	GTCGCTCG	32		D7_416	AGATAGAA	TTCTATCT		AATGATACGGCGACCACCGAGATCTACACGTCGCTCGACACTCTTTCCCTACACGACGCTCTTCCGATC*T	CAAGCAGAAGACGGCATACGAGATTTCTATCTGTGACTGGAGTTCAGACGTGTGCTCTTCCGATC*T
D5_417	ACGTCGTA	33		D7_417	CTCGGTAC	GTACCGAG		AATGATACGGCGACCACCGAGATCTACACACGTCGTAACACTCTTTCCCTACACGACGCTCTTCCGATC*T	CAAGCAGAAGACGGCATACGAGATGTACCGAGGTGACTGGAGTTCAGACGTGTGCTCTTCCGATC*T
D5_418	ATTTCATC	34		D7_418	GAGGTTGT	ACAACCTC		AATGATACGGCGACCACCGAGATCTACACATTTCATCACACTCTTTCCCTACACGACGCTCTTCCGATC*T	CAAGCAGAAGACGGCATACGAGATACAACCTCGTGACTGGAGTTCAGACGTGTGCTCTTCCGATC*T
D5_419	GAGTAGCA	35		D7_419	GCATGCCG	CGGCATGC		AATGATACGGCGACCACCGAGATCTACACGAGTAGCAACACTCTTTCCCTACACGACGCTCTTCCGATC*T	CAAGCAGAAGACGGCATACGAGATCGGCATGCGTGACTGGAGTTCAGACGTGTGCTCTTCCGATC*T
D5_420	AGCTGTCC	36		D7_420	CGGTACAA	TTGTACCG		AATGATACGGCGACCACCGAGATCTACACAGCTGTCCACACTCTTTCCCTACACGACGCTCTTCCGATC*T	CAAGCAGAAGACGGCATACGAGATTTGTACCGGTGACTGGAGTTCAGACGTGTGCTCTTCCGATC*T
D5_421	GGTAGGTG	37		D7_421	GTAAATGC	GCATTTAC		AATGATACGGCGACCACCGAGATCTACACGGTAGGTGACACTCTTTCCCTACACGACGCTCTTCCGATC*T	CAAGCAGAAGACGGCATACGAGATGCATTTACGTGACTGGAGTTCAGACGTGTGCTCTTCCGATC*T
D5_422	ACGGAGAC	38		D7_422	GGAGGTAT	ATACCTCC		AATGATACGGCGACCACCGAGATCTACACACGGAGACACACTCTTTCCCTACACGACGCTCTTCCGATC*T	CAAGCAGAAGACGGCATACGAGATATACCTCCGTGACTGGAGTTCAGACGTGTGCTCTTCCGATC*T
D5_423	AGGTAGCG	39		D7_423	GACGTGCT	AGCACGTC		AATGATACGGCGACCACCGAGATCTACACAGGTAGCGACACTCTTTCCCTACACGACGCTCTTCCGATC*T	CAAGCAGAAGACGGCATACGAGATAGCACGTCGTGACTGGAGTTCAGACGTGTGCTCTTCCGATC*T
D5_424	TCGTTAGT	40		D7_424	GTGCTATG	CATAGCAC		AATGATACGGCGACCACCGAGATCTACACTCGTTAGTACACTCTTTCCCTACACGACGCTCTTCCGATC*T	CAAGCAGAAGACGGCATACGAGATCATAGCACGTGACTGGAGTTCAGACGTGTGCTCTTCCGATC*T
D5_425	ATGCACTG	41		D7_425	CCTCCTAA	TTAGGAGG		AATGATACGGCGACCACCGAGATCTACACATGCACTGACACTCTTTCCCTACACGACGCTCTTCCGATC*T	CAAGCAGAAGACGGCATACGAGATTTAGGAGGGTGACTGGAGTTCAGACGTGTGCTCTTCCGATC*T
D5_426	ATGCCAAA	42		D7_426	TTTCCAGT	ACTGGAAA		AATGATACGGCGACCACCGAGATCTACACATGCCAAAACACTCTTTCCCTACACGACGCTCTTCCGATC*T	CAAGCAGAAGACGGCATACGAGATACTGGAAAGTGACTGGAGTTCAGACGTGTGCTCTTCCGATC*T
D5_427	CCTACTAA	43		D7_427	GATATGTG	CACATATC		AATGATACGGCGACCACCGAGATCTACACCCTACTAAACACTCTTTCCCTACACGACGCTCTTCCGATC*T	CAAGCAGAAGACGGCATACGAGATCACATATCGTGACTGGAGTTCAGACGTGTGCTCTTCCGATC*T
D5_428	ATAAACTT	44		D7_428	CATGAATC	GATTCATG		AATGATACGGCGACCACCGAGATCTACACATAAACTTACACTCTTTCCCTACACGACGCTCTTCCGATC*T	CAAGCAGAAGACGGCATACGAGATGATTCATGGTGACTGGAGTTCAGACGTGTGCTCTTCCGATC*T
D5_429	AAATTCAC	45		D7_429	TTAGTAGA	TCTACTAA		AATGATACGGCGACCACCGAGATCTACACAAATTCACACACTCTTTCCCTACACGACGCTCTTCCGATC*T	CAAGCAGAAGACGGCATACGAGATTCTACTAAGTGACTGGAGTTCAGACGTGTGCTCTTCCGATC*T
D5_430	ACGGACGA	46		D7_430	CCTCTGCC	GGCAGAGG		AATGATACGGCGACCACCGAGATCTACACACGGACGAACACTCTTTCCCTACACGACGCTCTTCCGATC*T	CAAGCAGAAGACGGCATACGAGATGGCAGAGGGTGACTGGAGTTCAGACGTGTGCTCTTCCGATC*T
D5_431	TTCGTACG	47		D7_431	CCGGCTGG	CCAGCCGG		AATGATACGGCGACCACCGAGATCTACACTTCGTACGACACTCTTTCCCTACACGACGCTCTTCCGATC*T	CAAGCAGAAGACGGCATACGAGATCCAGCCGGGTGACTGGAGTTCAGACGTGTGCTCTTCCGATC*T
D5_432	CAATGATG	48		D7_432	CACTGCGA	TCGCAGTG		AATGATACGGCGACCACCGAGATCTACACCAATGATGACACTCTTTCCCTACACGACGCTCTTCCGATC*T	CAAGCAGAAGACGGCATACGAGATTCGCAGTGGTGACTGGAGTTCAGACGTGTGCTCTTCCGATC*T
D5_433	AGCCCAAT	49		D7_433	TTTACCGG	CCGGTAAA		AATGATACGGCGACCACCGAGATCTACACAGCCCAATACACTCTTTCCCTACACGACGCTCTTCCGATC*T	CAAGCAGAAGACGGCATACGAGATCCGGTAAAGTGACTGGAGTTCAGACGTGTGCTCTTCCGATC*T
D5_434	TGAAACCG	50		D7_434	TACGGAGC	GCTCCGTA		AATGATACGGCGACCACCGAGATCTACACTGAAACCGACACTCTTTCCCTACACGACGCTCTTCCGATC*T	CAAGCAGAAGACGGCATACGAGATGCTCCGTAGTGACTGGAGTTCAGACGTGTGCTCTTCCGATC*T
D5_435	TCAAAGCA	51		D7_435	CTTCGGTC	GACCGAAG		AATGATACGGCGACCACCGAGATCTACACTCAAAGCAACACTCTTTCCCTACACGACGCTCTTCCGATC*T	CAAGCAGAAGACGGCATACGAGATGACCGAAGGTGACTGGAGTTCAGACGTGTGCTCTTCCGATC*T
D5_436	TCAGGCCC	52		D7_436	GCACTATA	TATAGTGC		AATGATACGGCGACCACCGAGATCTACACTCAGGCCCACACTCTTTCCCTACACGACGCTCTTCCGATC*T	CAAGCAGAAGACGGCATACGAGATTATAGTGCGTGACTGGAGTTCAGACGTGTGCTCTTCCGATC*T
D5_437	CTGGATTT	53		D7_437	CTGATGCT	AGCATCAG		AATGATACGGCGACCACCGAGATCTACACCTGGATTTACACTCTTTCCCTACACGACGCTCTTCCGATC*T	CAAGCAGAAGACGGCATACGAGATAGCATCAGGTGACTGGAGTTCAGACGTGTGCTCTTCCGATC*T
D5_438	TGCTCCTT	54		D7_438	GGGACAAA	TTTGTCCC		AATGATACGGCGACCACCGAGATCTACACTGCTCCTTACACTCTTTCCCTACACGACGCTCTTCCGATC*T	CAAGCAGAAGACGGCATACGAGATTTTGTCCCGTGACTGGAGTTCAGACGTGTGCTCTTCCGATC*T
D5_439	GATACTCA	55		D7_439	TAGTATTT	AAATACTA		AATGATACGGCGACCACCGAGATCTACACGATACTCAACACTCTTTCCCTACACGACGCTCTTCCGATC*T	CAAGCAGAAGACGGCATACGAGATAAATACTAGTGACTGGAGTTCAGACGTGTGCTCTTCCGATC*T
D5_440	GTTTCCTA	56		D7_440	AGACACTA	TAGTGTCT		AATGATACGGCGACCACCGAGATCTACACGTTTCCTAACACTCTTTCCCTACACGACGCTCTTCCGATC*T	CAAGCAGAAGACGGCATACGAGATTAGTGTCTGTGACTGGAGTTCAGACGTGTGCTCTTCCGATC*T
D5_441	AGGCCGTT	57		D7_441	AGTAGTAG	CTACTACT		AATGATACGGCGACCACCGAGATCTACACAGGCCGTTACACTCTTTCCCTACACGACGCTCTTCCGATC*T	CAAGCAGAAGACGGCATACGAGATCTACTACTGTGACTGGAGTTCAGACGTGTGCTCTTCCGATC*T
D5_442	ACCCTCCA	58		D7_442	GAGGGCCG	CGGCCCTC		AATGATACGGCGACCACCGAGATCTACACACCCTCCAACACTCTTTCCCTACACGACGCTCTTCCGATC*T	CAAGCAGAAGACGGCATACGAGATCGGCCCTCGTGACTGGAGTTCAGACGTGTGCTCTTCCGATC*T
D5_443	CGGATCCC	59		D7_443	TTGTCCAA	TTGGACAA		AATGATACGGCGACCACCGAGATCTACACCGGATCCCACACTCTTTCCCTACACGACGCTCTTCCGATC*T	CAAGCAGAAGACGGCATACGAGATTTGGACAAGTGACTGGAGTTCAGACGTGTGCTCTTCCGATC*T
D5_444	TGCCATCC	60		D7_444	CGCAACTG	CAGTTGCG		AATGATACGGCGACCACCGAGATCTACACTGCCATCCACACTCTTTCCCTACACGACGCTCTTCCGATC*T	CAAGCAGAAGACGGCATACGAGATCAGTTGCGGTGACTGGAGTTCAGACGTGTGCTCTTCCGATC*T
D5_445	TACTGCCG	61		D7_445	CTTGCGTG	CACGCAAG		AATGATACGGCGACCACCGAGATCTACACTACTGCCGACACTCTTTCCCTACACGACGCTCTTCCGATC*T	CAAGCAGAAGACGGCATACGAGATCACGCAAGGTGACTGGAGTTCAGACGTGTGCTCTTCCGATC*T
D5_446	CGGACAAG	62		D7_446	CATATTCT	AGAATATG		AATGATACGGCGACCACCGAGATCTACACCGGACAAGACACTCTTTCCCTACACGACGCTCTTCCGATC*T	CAAGCAGAAGACGGCATACGAGATAGAATATGGTGACTGGAGTTCAGACGTGTGCTCTTCCGATC*T
D5_447	TACCTCCC	63		D7_447	TTCCTATA	TATAGGAA		AATGATACGGCGACCACCGAGATCTACACTACCTCCCACACTCTTTCCCTACACGACGCTCTTCCGATC*T	CAAGCAGAAGACGGCATACGAGATTATAGGAAGTGACTGGAGTTCAGACGTGTGCTCTTCCGATC*T
D5_448	GCGCGAAG	64		D7_448	GACTCCAC	GTGGAGTC		AATGATACGGCGACCACCGAGATCTACACGCGCGAAGACACTCTTTCCCTACACGACGCTCTTCCGATC*T	CAAGCAGAAGACGGCATACGAGATGTGGAGTCGTGACTGGAGTTCAGACGTGTGCTCTTCCGATC*T
D5_449	GATAGTGT	65		D7_449	CCACGCGC	GCGCGTGG		AATGATACGGCGACCACCGAGATCTACACGATAGTGTACACTCTTTCCCTACACGACGCTCTTCCGATC*T	CAAGCAGAAGACGGCATACGAGATGCGCGTGGGTGACTGGAGTTCAGACGTGTGCTCTTCCGATC*T
D5_450	ACAGCCCA	66		D7_450	TCTGGGCG	CGCCCAGA		AATGATACGGCGACCACCGAGATCTACACACAGCCCAACACTCTTTCCCTACACGACGCTCTTCCGATC*T	CAAGCAGAAGACGGCATACGAGATCGCCCAGAGTGACTGGAGTTCAGACGTGTGCTCTTCCGATC*T
D5_451	CCAATCGA	67		D7_451	CCAGTCTA	TAGACTGG		AATGATACGGCGACCACCGAGATCTACACCCAATCGAACACTCTTTCCCTACACGACGCTCTTCCGATC*T	CAAGCAGAAGACGGCATACGAGATTAGACTGGGTGACTGGAGTTCAGACGTGTGCTCTTCCGATC*T
D5_452	CCCACCCT	68		D7_452	CATCGTGC	GCACGATG		AATGATACGGCGACCACCGAGATCTACACCCCACCCTACACTCTTTCCCTACACGACGCTCTTCCGATC*T	CAAGCAGAAGACGGCATACGAGATGCACGATGGTGACTGGAGTTCAGACGTGTGCTCTTCCGATC*T
D5_453	CATTTATC	69		D7_453	CTGTCACA	TGTGACAG		AATGATACGGCGACCACCGAGATCTACACCATTTATCACACTCTTTCCCTACACGACGCTCTTCCGATC*T	CAAGCAGAAGACGGCATACGAGATTGTGACAGGTGACTGGAGTTCAGACGTGTGCTCTTCCGATC*T
D5_454	AGGTCTAC	70		D7_454	TATCGCGG	CCGCGATA		AATGATACGGCGACCACCGAGATCTACACAGGTCTACACACTCTTTCCCTACACGACGCTCTTCCGATC*T	CAAGCAGAAGACGGCATACGAGATCCGCGATAGTGACTGGAGTTCAGACGTGTGCTCTTCCGATC*T
D5_455	GTACCACG	71		D7_455	CACTGTCT	AGACAGTG		AATGATACGGCGACCACCGAGATCTACACGTACCACGACACTCTTTCCCTACACGACGCTCTTCCGATC*T	CAAGCAGAAGACGGCATACGAGATAGACAGTGGTGACTGGAGTTCAGACGTGTGCTCTTCCGATC*T
D5_456	TCTTGTCT	72		D7_456	CGAGTAAT	ATTACTCG		AATGATACGGCGACCACCGAGATCTACACTCTTGTCTACACTCTTTCCCTACACGACGCTCTTCCGATC*T	CAAGCAGAAGACGGCATACGAGATATTACTCGGTGACTGGAGTTCAGACGTGTGCTCTTCCGATC*T
D5_457	CATTGCAC	73		D7_457	GCGGAGAC	GTCTCCGC		AATGATACGGCGACCACCGAGATCTACACCATTGCACACACTCTTTCCCTACACGACGCTCTTCCGATC*T	CAAGCAGAAGACGGCATACGAGATGTCTCCGCGTGACTGGAGTTCAGACGTGTGCTCTTCCGATC*T
D5_458	ATAAAGCG	74		D7_458	TTGGGTGA	TCACCCAA		AATGATACGGCGACCACCGAGATCTACACATAAAGCGACACTCTTTCCCTACACGACGCTCTTCCGATC*T	CAAGCAGAAGACGGCATACGAGATTCACCCAAGTGACTGGAGTTCAGACGTGTGCTCTTCCGATC*T
D5_459	TGCATAAA	75		D7_459	GTTCAAAG	CTTTGAAC		AATGATACGGCGACCACCGAGATCTACACTGCATAAAACACTCTTTCCCTACACGACGCTCTTCCGATC*T	CAAGCAGAAGACGGCATACGAGATCTTTGAACGTGACTGGAGTTCAGACGTGTGCTCTTCCGATC*T
D5_460	GTCATCCT	76		D7_460	TTAACTTA	TAAGTTAA		AATGATACGGCGACCACCGAGATCTACACGTCATCCTACACTCTTTCCCTACACGACGCTCTTCCGATC*T	CAAGCAGAAGACGGCATACGAGATTAAGTTAAGTGACTGGAGTTCAGACGTGTGCTCTTCCGATC*T
D5_461	AGTTGGAG	77		D7_461	GCCTAGGG	CCCTAGGC		AATGATACGGCGACCACCGAGATCTACACAGTTGGAGACACTCTTTCCCTACACGACGCTCTTCCGATC*T	CAAGCAGAAGACGGCATACGAGATCCCTAGGCGTGACTGGAGTTCAGACGTGTGCTCTTCCGATC*T
D5_462	TGCGCTCA	78		D7_462	CGAGGGTT	AACCCTCG		AATGATACGGCGACCACCGAGATCTACACTGCGCTCAACACTCTTTCCCTACACGACGCTCTTCCGATC*T	CAAGCAGAAGACGGCATACGAGATAACCCTCGGTGACTGGAGTTCAGACGTGTGCTCTTCCGATC*T
D5_463	TGGTAAAG	79		D7_463	GAGCGAAC	GTTCGCTC		AATGATACGGCGACCACCGAGATCTACACTGGTAAAGACACTCTTTCCCTACACGACGCTCTTCCGATC*T	CAAGCAGAAGACGGCATACGAGATGTTCGCTCGTGACTGGAGTTCAGACGTGTGCTCTTCCGATC*T
D5_464	ACACCTTA	80		D7_464	GTCGCGAC	GTCGCGAC		AATGATACGGCGACCACCGAGATCTACACACACCTTAACACTCTTTCCCTACACGACGCTCTTCCGATC*T	CAAGCAGAAGACGGCATACGAGATGTCGCGACGTGACTGGAGTTCAGACGTGTGCTCTTCCGATC*T
D5_465	TCTGCCCT	81		D7_465	GTCTTGCC	GGCAAGAC		AATGATACGGCGACCACCGAGATCTACACTCTGCCCTACACTCTTTCCCTACACGACGCTCTTCCGATC*T	CAAGCAGAAGACGGCATACGAGATGGCAAGACGTGACTGGAGTTCAGACGTGTGCTCTTCCGATC*T
D5_466	TCACTGGA	82		D7_466	CCAGGTCG	CGACCTGG		AATGATACGGCGACCACCGAGATCTACACTCACTGGAACACTCTTTCCCTACACGACGCTCTTCCGATC*T	CAAGCAGAAGACGGCATACGAGATCGACCTGGGTGACTGGAGTTCAGACGTGTGCTCTTCCGATC*T

D5_467	ACCGATTA	83		D7_467	CGTGTGGA	TCCACACG		AATGATACGGCGACCACCGAGATCTACACACCGATTAACACTCTTTCCCTACACGACGCTCTTCCGATC*T	CAAGCAGAAGACGGCATACGAGATTCCACACGGTGACTGGAGTTCAGACGTGTGCTCTTCCGATC*T
D5_468	TTTAGACA	84		D7_468	AAGGCCGC	GCGGCCTT		AATGATACGGCGACCACCGAGATCTACACTTTAGACAACACTCTTTCCCTACACGACGCTCTTCCGATC*T	CAAGCAGAAGACGGCATACGAGATGCGGCCTTGTGACTGGAGTTCAGACGTGTGCTCTTCCGATC*T
D5_503	TCTCGACC	85		D7_1002	CTTACTCA	TGAGTAAG		AATGATACGGCGACCACCGAGATCTACACTCTCGACCACACTCTTTCCCTACACGACGCTCTTCCGATC*T	CAAGCAGAAGACGGCATACGAGATTGAGTAAGGTGACTGGAGTTCAGACGTGTGCTCTTCCGATC*T
D5_470	GTCTACAT	86		D7_470	GCGAATTG	CAATTCGC		AATGATACGGCGACCACCGAGATCTACACGTCTACATACACTCTTTCCCTACACGACGCTCTTCCGATC*T	CAAGCAGAAGACGGCATACGAGATCAATTCGCGTGACTGGAGTTCAGACGTGTGCTCTTCCGATC*T
D5_471	TCCCACAG	87		D7_471	CGTTTACG	CGTAAACG		AATGATACGGCGACCACCGAGATCTACACTCCCACAGACACTCTTTCCCTACACGACGCTCTTCCGATC*T	CAAGCAGAAGACGGCATACGAGATCGTAAACGGTGACTGGAGTTCAGACGTGTGCTCTTCCGATC*T
D5_472	ATGGTCCC	88		D7_472	CCAACGCT	AGCGTTGG		AATGATACGGCGACCACCGAGATCTACACATGGTCCCACACTCTTTCCCTACACGACGCTCTTCCGATC*T	CAAGCAGAAGACGGCATACGAGATAGCGTTGGGTGACTGGAGTTCAGACGTGTGCTCTTCCGATC*T
D5_473	CCCGAGAT	89		D7_473	GTGAAGCC	GGCTTCAC		AATGATACGGCGACCACCGAGATCTACACCCCGAGATACACTCTTTCCCTACACGACGCTCTTCCGATC*T	CAAGCAGAAGACGGCATACGAGATGGCTTCACGTGACTGGAGTTCAGACGTGTGCTCTTCCGATC*T
D5_474	TGCTATGA	90		D7_474	TTTGGCAC	GTGCCAAA		AATGATACGGCGACCACCGAGATCTACACTGCTATGAACACTCTTTCCCTACACGACGCTCTTCCGATC*T	CAAGCAGAAGACGGCATACGAGATGTGCCAAAGTGACTGGAGTTCAGACGTGTGCTCTTCCGATC*T
D5_475	CAGTGAAA	91		D7_475	AACAGAAT	ATTCTGTT		AATGATACGGCGACCACCGAGATCTACACCAGTGAAAACACTCTTTCCCTACACGACGCTCTTCCGATC*T	CAAGCAGAAGACGGCATACGAGATATTCTGTTGTGACTGGAGTTCAGACGTGTGCTCTTCCGATC*T
D5_525	ATGGCCAG	92		D7_476	AATTTGCC	GGCAAATT		AATGATACGGCGACCACCGAGATCTACACATGGCCAGACACTCTTTCCCTACACGACGCTCTTCCGATC*T	CAAGCAGAAGACGGCATACGAGATGGCAAATTGTGACTGGAGTTCAGACGTGTGCTCTTCCGATC*T
D5_477	ATTTGCCC	93		D7_477	CGCATGTA	TACATGCG		AATGATACGGCGACCACCGAGATCTACACATTTGCCCACACTCTTTCCCTACACGACGCTCTTCCGATC*T	CAAGCAGAAGACGGCATACGAGATTACATGCGGTGACTGGAGTTCAGACGTGTGCTCTTCCGATC*T
D5_478	CAACAGCA	94		D7_478	TGTTCTTG	CAAGAACA		AATGATACGGCGACCACCGAGATCTACACCAACAGCAACACTCTTTCCCTACACGACGCTCTTCCGATC*T	CAAGCAGAAGACGGCATACGAGATCAAGAACAGTGACTGGAGTTCAGACGTGTGCTCTTCCGATC*T
D5_479	ACCGTCGT	95		D7_479	CGGGAGTG	CACTCCCG		AATGATACGGCGACCACCGAGATCTACACACCGTCGTACACTCTTTCCCTACACGACGCTCTTCCGATC*T	CAAGCAGAAGACGGCATACGAGATCACTCCCGGTGACTGGAGTTCAGACGTGTGCTCTTCCGATC*T
D5_480	ATCACCCA	96		D7_480	GGTGATGT	ACATCACC		AATGATACGGCGACCACCGAGATCTACACATCACCCAACACTCTTTCCCTACACGACGCTCTTCCGATC*T	CAAGCAGAAGACGGCATACGAGATACATCACCGTGACTGGAGTTCAGACGTGTGCTCTTCCGATC*T

SetB

i5 barcodes i7 barcodes

	**Sequence For Oligo**				**Sequence Obtained**	**Sequence for oligo**		i5 oligo to order	i7 oligo to order
D5_097	ATCACGAT			D7_097	CCGTTAGT	ACTAACGG		AATGATACGGCGACCACCGAGATCTACACATCACGATACACTCTTTCCCTACACGACGCTCTTCCGATC*T	CAAGCAGAAGACGGCATACGAGATACTAACGGGTGACTGGAGTTCAGACGTGTGCTCTTCCGATC*T
D5_098	TGATCAAA			D7_098	TCGACTCG	CGAGTCGA		AATGATACGGCGACCACCGAGATCTACACTGATCAAAACACTCTTTCCCTACACGACGCTCTTCCGATC*T	CAAGCAGAAGACGGCATACGAGATCGAGTCGAGTGACTGGAGTTCAGACGTGTGCTCTTCCGATC*T
D5_099	CGACTTCC			D7_099	CGTAAAGA	TCTTTACG		AATGATACGGCGACCACCGAGATCTACACCGACTTCCACACTCTTTCCCTACACGACGCTCTTCCGATC*T	CAAGCAGAAGACGGCATACGAGATTCTTTACGGTGACTGGAGTTCAGACGTGTGCTCTTCCGATC*T
D5_100	CTCAGAAA			D7_100	CGTCGTCG	CGACGACG		AATGATACGGCGACCACCGAGATCTACACCTCAGAAAACACTCTTTCCCTACACGACGCTCTTCCGATC*T	CAAGCAGAAGACGGCATACGAGATCGACGACGGTGACTGGAGTTCAGACGTGTGCTCTTCCGATC*T
D5_101	TCAGCCTG			D7_101	TTCTCTTT	AAAGAGAA		AATGATACGGCGACCACCGAGATCTACACTCAGCCTGACACTCTTTCCCTACACGACGCTCTTCCGATC*T	CAAGCAGAAGACGGCATACGAGATAAAGAGAAGTGACTGGAGTTCAGACGTGTGCTCTTCCGATC*T
D5_102	TTTGCACC			D7_102	TCTCATAT	ATATGAGA		AATGATACGGCGACCACCGAGATCTACACTTTGCACCACACTCTTTCCCTACACGACGCTCTTCCGATC*T	CAAGCAGAAGACGGCATACGAGATATATGAGAGTGACTGGAGTTCAGACGTGTGCTCTTCCGATC*T
D5_103	CGATCTGG			D7_103	TCGGGCTG	CAGCCCGA		AATGATACGGCGACCACCGAGATCTACACCGATCTGGACACTCTTTCCCTACACGACGCTCTTCCGATC*T	CAAGCAGAAGACGGCATACGAGATCAGCCCGAGTGACTGGAGTTCAGACGTGTGCTCTTCCGATC*T
D5_104	GGCGTCCC			D7_104	GGAACGAT	ATCGTTCC		AATGATACGGCGACCACCGAGATCTACACGGCGTCCCACACTCTTTCCCTACACGACGCTCTTCCGATC*T	CAAGCAGAAGACGGCATACGAGATATCGTTCCGTGACTGGAGTTCAGACGTGTGCTCTTCCGATC*T
D5_105	GAGCGCGC			D7_105	TGTTACAG	CTGTAACA		AATGATACGGCGACCACCGAGATCTACACGAGCGCGCACACTCTTTCCCTACACGACGCTCTTCCGATC*T	CAAGCAGAAGACGGCATACGAGATCTGTAACAGTGACTGGAGTTCAGACGTGTGCTCTTCCGATC*T
D5_106	TGGTGCAC			D7_106	TCCACAAA	TTTGTGGA		AATGATACGGCGACCACCGAGATCTACACTGGTGCACACACTCTTTCCCTACACGACGCTCTTCCGATC*T	CAAGCAGAAGACGGCATACGAGATTTTGTGGAGTGACTGGAGTTCAGACGTGTGCTCTTCCGATC*T
D5_107	TGGTGACT			D7_107	CGCCTTGA	TCAAGGCG		AATGATACGGCGACCACCGAGATCTACACTGGTGACTACACTCTTTCCCTACACGACGCTCTTCCGATC*T	CAAGCAGAAGACGGCATACGAGATTCAAGGCGGTGACTGGAGTTCAGACGTGTGCTCTTCCGATC*T
D5_108	CGTCAGAG			D7_108	CTCGCAAG	CTTGCGAG		AATGATACGGCGACCACCGAGATCTACACCGTCAGAGACACTCTTTCCCTACACGACGCTCTTCCGATC*T	CAAGCAGAAGACGGCATACGAGATCTTGCGAGGTGACTGGAGTTCAGACGTGTGCTCTTCCGATC*T
D5_109	CTCCTACA			D7_109	AGACGGAG	CTCCGTCT		AATGATACGGCGACCACCGAGATCTACACCTCCTACAACACTCTTTCCCTACACGACGCTCTTCCGATC*T	CAAGCAGAAGACGGCATACGAGATCTCCGTCTGTGACTGGAGTTCAGACGTGTGCTCTTCCGATC*T
D5_110	AAAGCAGT			D7_110	AGTGAGTA	TACTCACT		AATGATACGGCGACCACCGAGATCTACACAAAGCAGTACACTCTTTCCCTACACGACGCTCTTCCGATC*T	CAAGCAGAAGACGGCATACGAGATTACTCACTGTGACTGGAGTTCAGACGTGTGCTCTTCCGATC*T
D5_111	CGGTTATA			D7_111	CTTCAATA	TATTGAAG		AATGATACGGCGACCACCGAGATCTACACCGGTTATAACACTCTTTCCCTACACGACGCTCTTCCGATC*T	CAAGCAGAAGACGGCATACGAGATTATTGAAGGTGACTGGAGTTCAGACGTGTGCTCTTCCGATC*T
D5_112	CACGTTTA			D7_112	CACTCGTT	AACGAGTG		AATGATACGGCGACCACCGAGATCTACACCACGTTTAACACTCTTTCCCTACACGACGCTCTTCCGATC*T	CAAGCAGAAGACGGCATACGAGATAACGAGTGGTGACTGGAGTTCAGACGTGTGCTCTTCCGATC*T
D5_113	TTGGTAGA			D7_113	CCATGGCC	GGCCATGG		AATGATACGGCGACCACCGAGATCTACACTTGGTAGAACACTCTTTCCCTACACGACGCTCTTCCGATC*T	CAAGCAGAAGACGGCATACGAGATGGCCATGGGTGACTGGAGTTCAGACGTGTGCTCTTCCGATC*T
D5_114	TCCTGCGT			D7_114	GCTTGCAT	ATGCAAGC		AATGATACGGCGACCACCGAGATCTACACTCCTGCGTACACTCTTTCCCTACACGACGCTCTTCCGATC*T	CAAGCAGAAGACGGCATACGAGATATGCAAGCGTGACTGGAGTTCAGACGTGTGCTCTTCCGATC*T
D5_115	TTATCTTG			D7_115	GGGCGTGC	GCACGCCC		AATGATACGGCGACCACCGAGATCTACACTTATCTTGACACTCTTTCCCTACACGACGCTCTTCCGATC*T	CAAGCAGAAGACGGCATACGAGATGCACGCCCGTGACTGGAGTTCAGACGTGTGCTCTTCCGATC*T
D5_116	GCAGTCCG			D7_116	CGTTCTGA	TCAGAACG		AATGATACGGCGACCACCGAGATCTACACGCAGTCCGACACTCTTTCCCTACACGACGCTCTTCCGATC*T	CAAGCAGAAGACGGCATACGAGATTCAGAACGGTGACTGGAGTTCAGACGTGTGCTCTTCCGATC*T
D5_117	TCTAGGTA			D7_117	TTATATCA	TGATATAA		AATGATACGGCGACCACCGAGATCTACACTCTAGGTAACACTCTTTCCCTACACGACGCTCTTCCGATC*T	CAAGCAGAAGACGGCATACGAGATTGATATAAGTGACTGGAGTTCAGACGTGTGCTCTTCCGATC*T
D5_118	TCTGATCA			D7_118	GCTAAGTT	AACTTAGC		AATGATACGGCGACCACCGAGATCTACACTCTGATCAACACTCTTTCCCTACACGACGCTCTTCCGATC*T	CAAGCAGAAGACGGCATACGAGATAACTTAGCGTGACTGGAGTTCAGACGTGTGCTCTTCCGATC*T
D5_119	ACCTCGAG			D7_119	GCGCCAAG	CTTGGCGC		AATGATACGGCGACCACCGAGATCTACACACCTCGAGACACTCTTTCCCTACACGACGCTCTTCCGATC*T	CAAGCAGAAGACGGCATACGAGATCTTGGCGCGTGACTGGAGTTCAGACGTGTGCTCTTCCGATC*T
D5_120	AGTAAGAC			D7_120	GGCTTCCA	TGGAAGCC		AATGATACGGCGACCACCGAGATCTACACAGTAAGACACACTCTTTCCCTACACGACGCTCTTCCGATC*T	CAAGCAGAAGACGGCATACGAGATTGGAAGCCGTGACTGGAGTTCAGACGTGTGCTCTTCCGATC*T
D5_121	GATTGACC			D7_121	CACCTATC	GATAGGTG		AATGATACGGCGACCACCGAGATCTACACGATTGACCACACTCTTTCCCTACACGACGCTCTTCCGATC*T	CAAGCAGAAGACGGCATACGAGATGATAGGTGGTGACTGGAGTTCAGACGTGTGCTCTTCCGATC*T
D5_122	TCGTGATC			D7_122	GGCTGAAT	ATTCAGCC		AATGATACGGCGACCACCGAGATCTACACTCGTGATCACACTCTTTCCCTACACGACGCTCTTCCGATC*T	CAAGCAGAAGACGGCATACGAGATATTCAGCCGTGACTGGAGTTCAGACGTGTGCTCTTCCGATC*T
D5_123	ACGTCTGT			D7_123	ACGTTGCT	AGCAACGT		AATGATACGGCGACCACCGAGATCTACACACGTCTGTACACTCTTTCCCTACACGACGCTCTTCCGATC*T	CAAGCAGAAGACGGCATACGAGATAGCAACGTGTGACTGGAGTTCAGACGTGTGCTCTTCCGATC*T
D5_124	TACAAACA			D7_124	CGCTTGAG	CTCAAGCG		AATGATACGGCGACCACCGAGATCTACACTACAAACAACACTCTTTCCCTACACGACGCTCTTCCGATC*T	CAAGCAGAAGACGGCATACGAGATCTCAAGCGGTGACTGGAGTTCAGACGTGTGCTCTTCCGATC*T
D5_125	TCTACAGA			D7_125	CGACGAGT	ACTCGTCG		AATGATACGGCGACCACCGAGATCTACACTCTACAGAACACTCTTTCCCTACACGACGCTCTTCCGATC*T	CAAGCAGAAGACGGCATACGAGATACTCGTCGGTGACTGGAGTTCAGACGTGTGCTCTTCCGATC*T
D5_126	ACCCATCG			D7_126	GTGCATTT	AAATGCAC		AATGATACGGCGACCACCGAGATCTACACACCCATCGACACTCTTTCCCTACACGACGCTCTTCCGATC*T	CAAGCAGAAGACGGCATACGAGATAAATGCACGTGACTGGAGTTCAGACGTGTGCTCTTCCGATC*T
D5_127	TCTCCGCA			D7_127	AGCATCGG	CCGATGCT		AATGATACGGCGACCACCGAGATCTACACTCTCCGCAACACTCTTTCCCTACACGACGCTCTTCCGATC*T	CAAGCAGAAGACGGCATACGAGATCCGATGCTGTGACTGGAGTTCAGACGTGTGCTCTTCCGATC*T
D5_128	ATGTACCT			D7_128	CACGGGTA	TACCCGTG		AATGATACGGCGACCACCGAGATCTACACATGTACCTACACTCTTTCCCTACACGACGCTCTTCCGATC*T	CAAGCAGAAGACGGCATACGAGATTACCCGTGGTGACTGGAGTTCAGACGTGTGCTCTTCCGATC*T
D5_129	TTGACCGG			D7_129	GGAGCAAG	CTTGCTCC		AATGATACGGCGACCACCGAGATCTACACTTGACCGGACACTCTTTCCCTACACGACGCTCTTCCGATC*T	CAAGCAGAAGACGGCATACGAGATCTTGCTCCGTGACTGGAGTTCAGACGTGTGCTCTTCCGATC*T
D5_130	ACACGACG			D7_130	GGGTACTT	AAGTACCC		AATGATACGGCGACCACCGAGATCTACACACACGACGACACTCTTTCCCTACACGACGCTCTTCCGATC*T	CAAGCAGAAGACGGCATACGAGATAAGTACCCGTGACTGGAGTTCAGACGTGTGCTCTTCCGATC*T
D5_131	TTCGTTGT			D7_131	TTAATCTC	GAGATTAA		AATGATACGGCGACCACCGAGATCTACACTTCGTTGTACACTCTTTCCCTACACGACGCTCTTCCGATC*T	CAAGCAGAAGACGGCATACGAGATGAGATTAAGTGACTGGAGTTCAGACGTGTGCTCTTCCGATC*T
D5_132	CTTGGCCG			D7_132	TTTATGGT	ACCATAAA		AATGATACGGCGACCACCGAGATCTACACCTTGGCCGACACTCTTTCCCTACACGACGCTCTTCCGATC*T	CAAGCAGAAGACGGCATACGAGATACCATAAAGTGACTGGAGTTCAGACGTGTGCTCTTCCGATC*T
D5_133	TTATACAC			D7_133	GTATGAGT	ACTCATAC		AATGATACGGCGACCACCGAGATCTACACTTATACACACACTCTTTCCCTACACGACGCTCTTCCGATC*T	CAAGCAGAAGACGGCATACGAGATACTCATACGTGACTGGAGTTCAGACGTGTGCTCTTCCGATC*T
D5_134	TCCATAGG			D7_134	CTGTTCGC	GCGAACAG		AATGATACGGCGACCACCGAGATCTACACTCCATAGGACACTCTTTCCCTACACGACGCTCTTCCGATC*T	CAAGCAGAAGACGGCATACGAGATGCGAACAGGTGACTGGAGTTCAGACGTGTGCTCTTCCGATC*T
D5_135	GCTAACCT			D7_135	TATATTTA	TAAATATA		AATGATACGGCGACCACCGAGATCTACACGCTAACCTACACTCTTTCCCTACACGACGCTCTTCCGATC*T	CAAGCAGAAGACGGCATACGAGATTAAATATAGTGACTGGAGTTCAGACGTGTGCTCTTCCGATC*T
D5_136	AGCGTTGG			D7_136	AGACCATG	CATGGTCT		AATGATACGGCGACCACCGAGATCTACACAGCGTTGGACACTCTTTCCCTACACGACGCTCTTCCGATC*T	CAAGCAGAAGACGGCATACGAGATCATGGTCTGTGACTGGAGTTCAGACGTGTGCTCTTCCGATC*T

D5_137	TTTGCTGA			D7_137	GGTATGCA	TGCATACC		AATGATACGGCGACCACCGAGATCTACACTTTGCTGAACACTCTTTCCCTACACGACGCTCTTCCGATC*T	CAAGCAGAAGACGGCATACGAGATTGCATACCGTGACTGGAGTTCAGACGTGTGCTCTTCCGATC*T
D5_138	CTAAGATC			D7_138	TTTGGACT	AGTCCAAA		AATGATACGGCGACCACCGAGATCTACACCTAAGATCACACTCTTTCCCTACACGACGCTCTTCCGATC*T	CAAGCAGAAGACGGCATACGAGATAGTCCAAAGTGACTGGAGTTCAGACGTGTGCTCTTCCGATC*T
D5_139	AGCTCCAA			D7_139	CCGAATAA	TTATTCGG		AATGATACGGCGACCACCGAGATCTACACAGCTCCAAACACTCTTTCCCTACACGACGCTCTTCCGATC*T	CAAGCAGAAGACGGCATACGAGATTTATTCGGGTGACTGGAGTTCAGACGTGTGCTCTTCCGATC*T
D5_140	CGCCCTCG			D7_140	GGTCGCTC	GAGCGACC		AATGATACGGCGACCACCGAGATCTACACCGCCCTCGACACTCTTTCCCTACACGACGCTCTTCCGATC*T	CAAGCAGAAGACGGCATACGAGATGAGCGACCGTGACTGGAGTTCAGACGTGTGCTCTTCCGATC*T
D5_141	ATATGGTG			D7_141	ATCAATCC	GGATTGAT		AATGATACGGCGACCACCGAGATCTACACATATGGTGACACTCTTTCCCTACACGACGCTCTTCCGATC*T	CAAGCAGAAGACGGCATACGAGATGGATTGATGTGACTGGAGTTCAGACGTGTGCTCTTCCGATC*T
D5_142	ACTCCTAC			D7_142	TGGGCCTA	TAGGCCCA		AATGATACGGCGACCACCGAGATCTACACACTCCTACACACTCTTTCCCTACACGACGCTCTTCCGATC*T	CAAGCAGAAGACGGCATACGAGATTAGGCCCAGTGACTGGAGTTCAGACGTGTGCTCTTCCGATC*T
D5_143	TCATGTAC			D7_143	CGGCACTC	GAGTGCCG		AATGATACGGCGACCACCGAGATCTACACTCATGTACACACTCTTTCCCTACACGACGCTCTTCCGATC*T	CAAGCAGAAGACGGCATACGAGATGAGTGCCGGTGACTGGAGTTCAGACGTGTGCTCTTCCGATC*T
D5_144	ATTGTAAT			D7_144	CAAATAGC	GCTATTTG		AATGATACGGCGACCACCGAGATCTACACATTGTAATACACTCTTTCCCTACACGACGCTCTTCCGATC*T	CAAGCAGAAGACGGCATACGAGATGCTATTTGGTGACTGGAGTTCAGACGTGTGCTCTTCCGATC*T
D5_145	CCGCAGTA			D7_145	CCTTCGGG	CCCGAAGG		AATGATACGGCGACCACCGAGATCTACACCCGCAGTAACACTCTTTCCCTACACGACGCTCTTCCGATC*T	CAAGCAGAAGACGGCATACGAGATCCCGAAGGGTGACTGGAGTTCAGACGTGTGCTCTTCCGATC*T
D5_146	TCATTCAG			D7_146	TTTAGCTT	AAGCTAAA		AATGATACGGCGACCACCGAGATCTACACTCATTCAGACACTCTTTCCCTACACGACGCTCTTCCGATC*T	CAAGCAGAAGACGGCATACGAGATAAGCTAAAGTGACTGGAGTTCAGACGTGTGCTCTTCCGATC*T
D5_147	ACACAAGA			D7_147	AATAATGG	CCATTATT		AATGATACGGCGACCACCGAGATCTACACACACAAGAACACTCTTTCCCTACACGACGCTCTTCCGATC*T	CAAGCAGAAGACGGCATACGAGATCCATTATTGTGACTGGAGTTCAGACGTGTGCTCTTCCGATC*T
D5_148	GGCCAAAC			D7_148	GGTTCGCC	GGCGAACC		AATGATACGGCGACCACCGAGATCTACACGGCCAAACACACTCTTTCCCTACACGACGCTCTTCCGATC*T	CAAGCAGAAGACGGCATACGAGATGGCGAACCGTGACTGGAGTTCAGACGTGTGCTCTTCCGATC*T
D5_149	CCGTAAAT			D7_149	GCGTGAGG	CCTCACGC		AATGATACGGCGACCACCGAGATCTACACCCGTAAATACACTCTTTCCCTACACGACGCTCTTCCGATC*T	CAAGCAGAAGACGGCATACGAGATCCTCACGCGTGACTGGAGTTCAGACGTGTGCTCTTCCGATC*T
D5_150	AGAATAAT			D7_150	GGGACTTT	AAAGTCCC		AATGATACGGCGACCACCGAGATCTACACAGAATAATACACTCTTTCCCTACACGACGCTCTTCCGATC*T	CAAGCAGAAGACGGCATACGAGATAAAGTCCCGTGACTGGAGTTCAGACGTGTGCTCTTCCGATC*T
D5_151	ACTCAGTT			D7_151	CTGGGTCT	AGACCCAG		AATGATACGGCGACCACCGAGATCTACACACTCAGTTACACTCTTTCCCTACACGACGCTCTTCCGATC*T	CAAGCAGAAGACGGCATACGAGATAGACCCAGGTGACTGGAGTTCAGACGTGTGCTCTTCCGATC*T
D5_152	CACCACGG			D7_152	GATATAAC	GTTATATC		AATGATACGGCGACCACCGAGATCTACACCACCACGGACACTCTTTCCCTACACGACGCTCTTCCGATC*T	CAAGCAGAAGACGGCATACGAGATGTTATATCGTGACTGGAGTTCAGACGTGTGCTCTTCCGATC*T
D5_153	AGGTATTT			D7_153	AATGCGCA	TGCGCATT		AATGATACGGCGACCACCGAGATCTACACAGGTATTTACACTCTTTCCCTACACGACGCTCTTCCGATC*T	CAAGCAGAAGACGGCATACGAGATTGCGCATTGTGACTGGAGTTCAGACGTGTGCTCTTCCGATC*T
D5_154	AGAACGAG			D7_154	GCAATAGG	CCTATTGC		AATGATACGGCGACCACCGAGATCTACACAGAACGAGACACTCTTTCCCTACACGACGCTCTTCCGATC*T	CAAGCAGAAGACGGCATACGAGATCCTATTGCGTGACTGGAGTTCAGACGTGTGCTCTTCCGATC*T
D5_155	CGGAGCTG			D7_155	GGAGTGGT	ACCACTCC		AATGATACGGCGACCACCGAGATCTACACCGGAGCTGACACTCTTTCCCTACACGACGCTCTTCCGATC*T	CAAGCAGAAGACGGCATACGAGATACCACTCCGTGACTGGAGTTCAGACGTGTGCTCTTCCGATC*T
D5_156	GCCCTCTG			D7_156	TCGTAAAC	GTTTACGA		AATGATACGGCGACCACCGAGATCTACACGCCCTCTGACACTCTTTCCCTACACGACGCTCTTCCGATC*T	CAAGCAGAAGACGGCATACGAGATGTTTACGAGTGACTGGAGTTCAGACGTGTGCTCTTCCGATC*T
D5_157	TCTACTTT			D7_157	GCCGCCTG	CAGGCGGC		AATGATACGGCGACCACCGAGATCTACACTCTACTTTACACTCTTTCCCTACACGACGCTCTTCCGATC*T	CAAGCAGAAGACGGCATACGAGATCAGGCGGCGTGACTGGAGTTCAGACGTGTGCTCTTCCGATC*T
D5_158	AGCATCGA			D7_158	GCAAACTA	TAGTTTGC		AATGATACGGCGACCACCGAGATCTACACAGCATCGAACACTCTTTCCCTACACGACGCTCTTCCGATC*T	CAAGCAGAAGACGGCATACGAGATTAGTTTGCGTGACTGGAGTTCAGACGTGTGCTCTTCCGATC*T
D5_159	CGTGCTAC			D7_159	GCCGATGC	GCATCGGC		AATGATACGGCGACCACCGAGATCTACACCGTGCTACACACTCTTTCCCTACACGACGCTCTTCCGATC*T	CAAGCAGAAGACGGCATACGAGATGCATCGGCGTGACTGGAGTTCAGACGTGTGCTCTTCCGATC*T
D5_160	TGTCTCAC			D7_160	CACGCACC	GGTGCGTG		AATGATACGGCGACCACCGAGATCTACACTGTCTCACACACTCTTTCCCTACACGACGCTCTTCCGATC*T	CAAGCAGAAGACGGCATACGAGATGGTGCGTGGTGACTGGAGTTCAGACGTGTGCTCTTCCGATC*T
D5_161	ACTATCCC			D7_161	CGTAGCCT	AGGCTACG		AATGATACGGCGACCACCGAGATCTACACACTATCCCACACTCTTTCCCTACACGACGCTCTTCCGATC*T	CAAGCAGAAGACGGCATACGAGATAGGCTACGGTGACTGGAGTTCAGACGTGTGCTCTTCCGATC*T
D5_162	GTAACAGA			D7_162	TCTGAATA	TATTCAGA		AATGATACGGCGACCACCGAGATCTACACGTAACAGAACACTCTTTCCCTACACGACGCTCTTCCGATC*T	CAAGCAGAAGACGGCATACGAGATTATTCAGAGTGACTGGAGTTCAGACGTGTGCTCTTCCGATC*T
D5_163	AGGTTCAT			D7_163	GGGAAAGG	CCTTTCCC		AATGATACGGCGACCACCGAGATCTACACAGGTTCATACACTCTTTCCCTACACGACGCTCTTCCGATC*T	CAAGCAGAAGACGGCATACGAGATCCTTTCCCGTGACTGGAGTTCAGACGTGTGCTCTTCCGATC*T
D5_164	CCTCCCAT			D7_164	CTGGTATA	TATACCAG		AATGATACGGCGACCACCGAGATCTACACCCTCCCATACACTCTTTCCCTACACGACGCTCTTCCGATC*T	CAAGCAGAAGACGGCATACGAGATTATACCAGGTGACTGGAGTTCAGACGTGTGCTCTTCCGATC*T
D5_165	ATGTTATT			D7_165	TTTGGGTA	TACCCAAA		AATGATACGGCGACCACCGAGATCTACACATGTTATTACACTCTTTCCCTACACGACGCTCTTCCGATC*T	CAAGCAGAAGACGGCATACGAGATTACCCAAAGTGACTGGAGTTCAGACGTGTGCTCTTCCGATC*T
D5_166	AAGACCCT			D7_166	CCAGTGAG	CTCACTGG		AATGATACGGCGACCACCGAGATCTACACAAGACCCTACACTCTTTCCCTACACGACGCTCTTCCGATC*T	CAAGCAGAAGACGGCATACGAGATCTCACTGGGTGACTGGAGTTCAGACGTGTGCTCTTCCGATC*T
D5_167	CGGACTTC			D7_167	GTGGGAAA	TTTCCCAC		AATGATACGGCGACCACCGAGATCTACACCGGACTTCACACTCTTTCCCTACACGACGCTCTTCCGATC*T	CAAGCAGAAGACGGCATACGAGATTTTCCCACGTGACTGGAGTTCAGACGTGTGCTCTTCCGATC*T
D5_168	GTCCCTAC			D7_168	CGGACCGT	ACGGTCCG		AATGATACGGCGACCACCGAGATCTACACGTCCCTACACACTCTTTCCCTACACGACGCTCTTCCGATC*T	CAAGCAGAAGACGGCATACGAGATACGGTCCGGTGACTGGAGTTCAGACGTGTGCTCTTCCGATC*T
D5_169	TGTTAACA			D7_169	GTTGACTG	CAGTCAAC		AATGATACGGCGACCACCGAGATCTACACTGTTAACAACACTCTTTCCCTACACGACGCTCTTCCGATC*T	CAAGCAGAAGACGGCATACGAGATCAGTCAACGTGACTGGAGTTCAGACGTGTGCTCTTCCGATC*T
D5_170	ATTCGAAC			D7_170	CGGGCGGC	GCCGCCCG		AATGATACGGCGACCACCGAGATCTACACATTCGAACACACTCTTTCCCTACACGACGCTCTTCCGATC*T	CAAGCAGAAGACGGCATACGAGATGCCGCCCGGTGACTGGAGTTCAGACGTGTGCTCTTCCGATC*T
D5_171	TTTATGAC			D7_171	GAGACGCT	AGCGTCTC		AATGATACGGCGACCACCGAGATCTACACTTTATGACACACTCTTTCCCTACACGACGCTCTTCCGATC*T	CAAGCAGAAGACGGCATACGAGATAGCGTCTCGTGACTGGAGTTCAGACGTGTGCTCTTCCGATC*T
D5_172	TAATAGGC			D7_172	GCCAGTGG	CCACTGGC		AATGATACGGCGACCACCGAGATCTACACTAATAGGCACACTCTTTCCCTACACGACGCTCTTCCGATC*T	CAAGCAGAAGACGGCATACGAGATCCACTGGCGTGACTGGAGTTCAGACGTGTGCTCTTCCGATC*T
D5_173	ATACAGTA			D7_173	GGCGTTGG	CCAACGCC		AATGATACGGCGACCACCGAGATCTACACATACAGTAACACTCTTTCCCTACACGACGCTCTTCCGATC*T	CAAGCAGAAGACGGCATACGAGATCCAACGCCGTGACTGGAGTTCAGACGTGTGCTCTTCCGATC*T
D5_174	GCTAGAAC			D7_174	GGTAGTGA	TCACTACC		AATGATACGGCGACCACCGAGATCTACACGCTAGAACACACTCTTTCCCTACACGACGCTCTTCCGATC*T	CAAGCAGAAGACGGCATACGAGATTCACTACCGTGACTGGAGTTCAGACGTGTGCTCTTCCGATC*T
D5_175	TTAGAAAG			D7_175	ACGCGTCA	TGACGCGT		AATGATACGGCGACCACCGAGATCTACACTTAGAAAGACACTCTTTCCCTACACGACGCTCTTCCGATC*T	CAAGCAGAAGACGGCATACGAGATTGACGCGTGTGACTGGAGTTCAGACGTGTGCTCTTCCGATC*T
D5_176	TATATACG			D7_176	GTGACTAC	GTAGTCAC		AATGATACGGCGACCACCGAGATCTACACTATATACGACACTCTTTCCCTACACGACGCTCTTCCGATC*T	CAAGCAGAAGACGGCATACGAGATGTAGTCACGTGACTGGAGTTCAGACGTGTGCTCTTCCGATC*T
D5_177	ACGAAGTG			D7_177	GTTTGGGA	TCCCAAAC		AATGATACGGCGACCACCGAGATCTACACACGAAGTGACACTCTTTCCCTACACGACGCTCTTCCGATC*T	CAAGCAGAAGACGGCATACGAGATTCCCAAACGTGACTGGAGTTCAGACGTGTGCTCTTCCGATC*T
D5_178	TTCTCCAG			D7_178	GCACACAC	GTGTGTGC		AATGATACGGCGACCACCGAGATCTACACTTCTCCAGACACTCTTTCCCTACACGACGCTCTTCCGATC*T	CAAGCAGAAGACGGCATACGAGATGTGTGTGCGTGACTGGAGTTCAGACGTGTGCTCTTCCGATC*T
D5_179	GGCGTAAG			D7_179	CTTTAACC	GGTTAAAG		AATGATACGGCGACCACCGAGATCTACACGGCGTAAGACACTCTTTCCCTACACGACGCTCTTCCGATC*T	CAAGCAGAAGACGGCATACGAGATGGTTAAAGGTGACTGGAGTTCAGACGTGTGCTCTTCCGATC*T
D5_180	TCTCACTA			D7_180	TGTAGGTG	CACCTACA		AATGATACGGCGACCACCGAGATCTACACTCTCACTAACACTCTTTCCCTACACGACGCTCTTCCGATC*T	CAAGCAGAAGACGGCATACGAGATCACCTACAGTGACTGGAGTTCAGACGTGTGCTCTTCCGATC*T
D5_181	ATGAATAA			D7_181	TTCACGTC	GACGTGAA		AATGATACGGCGACCACCGAGATCTACACATGAATAAACACTCTTTCCCTACACGACGCTCTTCCGATC*T	CAAGCAGAAGACGGCATACGAGATGACGTGAAGTGACTGGAGTTCAGACGTGTGCTCTTCCGATC*T
D5_182	ATAACAAC			D7_182	GATGAGCG	CGCTCATC		AATGATACGGCGACCACCGAGATCTACACATAACAACACACTCTTTCCCTACACGACGCTCTTCCGATC*T	CAAGCAGAAGACGGCATACGAGATCGCTCATCGTGACTGGAGTTCAGACGTGTGCTCTTCCGATC*T
D5_183	CTATAGCC			D7_183	GGCGCCGC	GCGGCGCC		AATGATACGGCGACCACCGAGATCTACACCTATAGCCACACTCTTTCCCTACACGACGCTCTTCCGATC*T	CAAGCAGAAGACGGCATACGAGATGCGGCGCCGTGACTGGAGTTCAGACGTGTGCTCTTCCGATC*T
D5_184	TAAGTCGG			D7_184	AACCTGTG	CACAGGTT		AATGATACGGCGACCACCGAGATCTACACTAAGTCGGACACTCTTTCCCTACACGACGCTCTTCCGATC*T	CAAGCAGAAGACGGCATACGAGATCACAGGTTGTGACTGGAGTTCAGACGTGTGCTCTTCCGATC*T
D5_185	TGAACTGA			D7_185	GGTTAAGC	GCTTAACC		AATGATACGGCGACCACCGAGATCTACACTGAACTGAACACTCTTTCCCTACACGACGCTCTTCCGATC*T	CAAGCAGAAGACGGCATACGAGATGCTTAACCGTGACTGGAGTTCAGACGTGTGCTCTTCCGATC*T
D5_186	ATTTCCAT			D7_186	CGGGTCTT	AAGACCCG		AATGATACGGCGACCACCGAGATCTACACATTTCCATACACTCTTTCCCTACACGACGCTCTTCCGATC*T	CAAGCAGAAGACGGCATACGAGATAAGACCCGGTGACTGGAGTTCAGACGTGTGCTCTTCCGATC*T
D5_187	TCTTTCGC			D7_187	CTTGTCCT	AGGACAAG		AATGATACGGCGACCACCGAGATCTACACTCTTTCGCACACTCTTTCCCTACACGACGCTCTTCCGATC*T	CAAGCAGAAGACGGCATACGAGATAGGACAAGGTGACTGGAGTTCAGACGTGTGCTCTTCCGATC*T
D5_188	TTCAGCCC			D7_188	AAAGACAC	GTGTCTTT		AATGATACGGCGACCACCGAGATCTACACTTCAGCCCACACTCTTTCCCTACACGACGCTCTTCCGATC*T	CAAGCAGAAGACGGCATACGAGATGTGTCTTTGTGACTGGAGTTCAGACGTGTGCTCTTCCGATC*T
D5_189	CGTATCAG			D7_189	TCAGTTTC	GAAACTGA		AATGATACGGCGACCACCGAGATCTACACCGTATCAGACACTCTTTCCCTACACGACGCTCTTCCGATC*T	CAAGCAGAAGACGGCATACGAGATGAAACTGAGTGACTGGAGTTCAGACGTGTGCTCTTCCGATC*T
D5_190	ATACTTAC			D7_190	CGGTCCTG	CAGGACCG		AATGATACGGCGACCACCGAGATCTACACATACTTACACACTCTTTCCCTACACGACGCTCTTCCGATC*T	CAAGCAGAAGACGGCATACGAGATCAGGACCGGTGACTGGAGTTCAGACGTGTGCTCTTCCGATC*T
D5_191	CACCCATG			D7_191	CCAGCATC	GATGCTGG		AATGATACGGCGACCACCGAGATCTACACCACCCATGACACTCTTTCCCTACACGACGCTCTTCCGATC*T	CAAGCAGAAGACGGCATACGAGATGATGCTGGGTGACTGGAGTTCAGACGTGTGCTCTTCCGATC*T
D5_192	TCATAAGG			D7_192	CTGCCGAG	CTCGGCAG		AATGATACGGCGACCACCGAGATCTACACTCATAAGGACACTCTTTCCCTACACGACGCTCTTCCGATC*T	CAAGCAGAAGACGGCATACGAGATCTCGGCAGGTGACTGGAGTTCAGACGTGTGCTCTTCCGATC*T

SetC

i5 barcodes / i7 barcodes

	Sequence For Oligo				Sequence Obtained	Sequence for oligo		i5 oligo to order		i7 oligo to order
D5_193	CCATCCAA			D7_193	GGGATCCT	AGGATCCC		AATGATACGGCGACCACCGAGATCTACACCCATCCAAACACTCTTTCCCTACACGACGCTCTTCCGATC*T		CAAGCAGAAGACGGCATACGAGATAGGATCCCGTGACTGGAGTTCAGACGTGTGCTCTTCCGATC*T
D5_194	ACACCCAG			D7_194	GATACTCC	GGAGTATC		AATGATACGGCGACCACCGAGATCTACACACACCCAGACACTCTTTCCCTACACGACGCTCTTCCGATC*T		CAAGCAGAAGACGGCATACGAGATGGAGTATCGTGACTGGAGTTCAGACGTGTGCTCTTCCGATC*T
D5_195	CTCTCCTC			D7_195	GCCAAGCA	TGCTTGGC		AATGATACGGCGACCACCGAGATCTACACCTCTCCTCACACTCTTTCCCTACACGACGCTCTTCCGATC*T		CAAGCAGAAGACGGCATACGAGATTGCTTGGCGTGACTGGAGTTCAGACGTGTGCTCTTCCGATC*T
D5_196	CTTTGGCT			D7_196	GGCGGTCC	GGACCGCC		AATGATACGGCGACCACCGAGATCTACACCTTTGGCTACACTCTTTCCCTACACGACGCTCTTCCGATC*T		CAAGCAGAAGACGGCATACGAGATGGACCGCCGTGACTGGAGTTCAGACGTGTGCTCTTCCGATC*T
D5_197	ATCAAATA			D7_197	GCCTTGAA	TTCAAGGC		AATGATACGGCGACCACCGAGATCTACACATCAAATAACACTCTTTCCCTACACGACGCTCTTCCGATC*T		CAAGCAGAAGACGGCATACGAGATTTCAAGGCGTGACTGGAGTTCAGACGTGTGCTCTTCCGATC*T
D5_198	TGGTTTGC			D7_198	GCACTTGC	GCAAGTGC		AATGATACGGCGACCACCGAGATCTACACTGGTTTGCACACTCTTTCCCTACACGACGCTCTTCCGATC*T		CAAGCAGAAGACGGCATACGAGATGCAAGTGCGTGACTGGAGTTCAGACGTGTGCTCTTCCGATC*T
D5_199	TAGCAGGA			D7_199	CGCACTAC	GTAGTGCG		AATGATACGGCGACCACCGAGATCTACACTAGCAGGAACACTCTTTCCCTACACGACGCTCTTCCGATC*T		CAAGCAGAAGACGGCATACGAGATGTAGTGCGGTGACTGGAGTTCAGACGTGTGCTCTTCCGATC*T
D5_200	CGGATGGA			D7_200	CGCGGCCG	CGGCCGCG		AATGATACGGCGACCACCGAGATCTACACCGGATGGAACACTCTTTCCCTACACGACGCTCTTCCGATC*T		CAAGCAGAAGACGGCATACGAGATCGGCCGCGGTGACTGGAGTTCAGACGTGTGCTCTTCCGATC*T
D5_201	TAGGTCTA			D7_201	CGGCTTCT	AGAAGCCG		AATGATACGGCGACCACCGAGATCTACACTAGGTCTAACACTCTTTCCCTACACGACGCTCTTCCGATC*T		CAAGCAGAAGACGGCATACGAGATAGAAGCCGGTGACTGGAGTTCAGACGTGTGCTCTTCCGATC*T
D5_202	TGCATGGC			D7_202	GAGTGCGT	ACGCACTC		AATGATACGGCGACCACCGAGATCTACACTGCATGGCACACTCTTTCCCTACACGACGCTCTTCCGATC*T		CAAGCAGAAGACGGCATACGAGATACGCACTCGTGACTGGAGTTCAGACGTGTGCTCTTCCGATC*T
D5_203	ATCAGAGT			D7_203	CACAAACA	TGTTTGTG		AATGATACGGCGACCACCGAGATCTACACATCAGAGTACACTCTTTCCCTACACGACGCTCTTCCGATC*T		CAAGCAGAAGACGGCATACGAGATTGTTTGTGGTGACTGGAGTTCAGACGTGTGCTCTTCCGATC*T
D5_204	TCCATTTA			D7_204	TTAACACT	AGTGTTAA		AATGATACGGCGACCACCGAGATCTACACTCCATTTAACACTCTTTCCCTACACGACGCTCTTCCGATC*T		CAAGCAGAAGACGGCATACGAGATAGTGTTAAGTGACTGGAGTTCAGACGTGTGCTCTTCCGATC*T
D5_205	TCCTCTAA			D7_205	AGCGACCA	TGGTCGCT		AATGATACGGCGACCACCGAGATCTACACTCCTCTAAACACTCTTTCCCTACACGACGCTCTTCCGATC*T		CAAGCAGAAGACGGCATACGAGATTGGTCGCTGTGACTGGAGTTCAGACGTGTGCTCTTCCGATC*T
D5_206	CAAATCTT			D7_206	GTCACGGG	CCCGTGAC		AATGATACGGCGACCACCGAGATCTACACCAAATCTTACACTCTTTCCCTACACGACGCTCTTCCGATC*T		CAAGCAGAAGACGGCATACGAGATCCCGTGACGTGACTGGAGTTCAGACGTGTGCTCTTCCGATC*T
D5_207	ATAGCTGG			D7_207	CAACCTGT	ACAGGTTG		AATGATACGGCGACCACCGAGATCTACACATAGCTGGACACTCTTTCCCTACACGACGCTCTTCCGATC*T		CAAGCAGAAGACGGCATACGAGATACAGGTTGGTGACTGGAGTTCAGACGTGTGCTCTTCCGATC*T
D5_208	ACCGCGGA			D7_208	CATGGGCT	AGCCCATG		AATGATACGGCGACCACCGAGATCTACACACCGCGGAACACTCTTTCCCTACACGACGCTCTTCCGATC*T		CAAGCAGAAGACGGCATACGAGATAGCCCATGGTGACTGGAGTTCAGACGTGTGCTCTTCCGATC*T
D5_209	TGACATGG			D7_209	GGATTCGC	GCGAATCC		AATGATACGGCGACCACCGAGATCTACACTGACATGGACACTCTTTCCCTACACGACGCTCTTCCGATC*T		CAAGCAGAAGACGGCATACGAGATGCGAATCCGTGACTGGAGTTCAGACGTGTGCTCTTCCGATC*T
D5_210	CGTATAGC			D7_210	AACTTTCG	CGAAAGTT		AATGATACGGCGACCACCGAGATCTACACCGTATAGCACACTCTTTCCCTACACGACGCTCTTCCGATC*T		CAAGCAGAAGACGGCATACGAGATCGAAAGTTGTGACTGGAGTTCAGACGTGTGCTCTTCCGATC*T
D5_211	CGACCAGA			D7_211	AAACGCGA	TCGCGTTT		AATGATACGGCGACCACCGAGATCTACACCGACCAGAACACTCTTTCCCTACACGACGCTCTTCCGATC*T		CAAGCAGAAGACGGCATACGAGATTCGCGTTTGTGACTGGAGTTCAGACGTGTGCTCTTCCGATC*T
D5_212	TGAGGCAG			D7_212	GCTCGATT	AATCGAGC		AATGATACGGCGACCACCGAGATCTACACTGAGGCAGACACTCTTTCCCTACACGACGCTCTTCCGATC*T		CAAGCAGAAGACGGCATACGAGATAATCGAGCGTGACTGGAGTTCAGACGTGTGCTCTTCCGATC*T
D5_213	TTACCTAA			D7_213	TGTCGTTT	AAACGACA		AATGATACGGCGACCACCGAGATCTACACTTACCTAAACACTCTTTCCCTACACGACGCTCTTCCGATC*T		CAAGCAGAAGACGGCATACGAGATAAACGACAGTGACTGGAGTTCAGACGTGTGCTCTTCCGATC*T
D5_214	TACAATGG			D7_214	GCACGGTC	GACCGTGC		AATGATACGGCGACCACCGAGATCTACACTACAATGGACACTCTTTCCCTACACGACGCTCTTCCGATC*T		CAAGCAGAAGACGGCATACGAGATGACCGTGCGTGACTGGAGTTCAGACGTGTGCTCTTCCGATC*T
D5_215	GAATATTA			D7_215	CGGGCTCA	TGAGCCCG		AATGATACGGCGACCACCGAGATCTACACGAATATTAACACTCTTTCCCTACACGACGCTCTTCCGATC*T		CAAGCAGAAGACGGCATACGAGATTGAGCCCGGTGACTGGAGTTCAGACGTGTGCTCTTCCGATC*T
D5_216	GAGAGTAG			D7_216	GCTGTTCA	TGAACAGC		AATGATACGGCGACCACCGAGATCTACACGAGAGTAGACACTCTTTCCCTACACGACGCTCTTCCGATC*T		CAAGCAGAAGACGGCATACGAGATTGAACAGCGTGACTGGAGTTCAGACGTGTGCTCTTCCGATC*T
D5_217	TGAAGAGC			D7_217	GTGCGTCG	CGACGCAC		AATGATACGGCGACCACCGAGATCTACACTGAAGAGCACACTCTTTCCCTACACGACGCTCTTCCGATC*T		CAAGCAGAAGACGGCATACGAGATCGACGCACGTGACTGGAGTTCAGACGTGTGCTCTTCCGATC*T
D5_218	CCACCATT			D7_218	CGCTAAAC	GTTTAGCG		AATGATACGGCGACCACCGAGATCTACACCCACCATTACACTCTTTCCCTACACGACGCTCTTCCGATC*T		CAAGCAGAAGACGGCATACGAGATGTTTAGCGGTGACTGGAGTTCAGACGTGTGCTCTTCCGATC*T
D5_219	TTGTCAAT			D7_219	CAGAATGC	GCATTCTG		AATGATACGGCGACCACCGAGATCTACACTTGTCAATACACTCTTTCCCTACACGACGCTCTTCCGATC*T		CAAGCAGAAGACGGCATACGAGATGCATTCTGGTGACTGGAGTTCAGACGTGTGCTCTTCCGATC*T
D5_220	TGAGAGAA			D7_220	GAAACCTG	CAGGTTTC		AATGATACGGCGACCACCGAGATCTACACTGAGAGAAACACTCTTTCCCTACACGACGCTCTTCCGATC*T		CAAGCAGAAGACGGCATACGAGATCAGGTTTCGTGACTGGAGTTCAGACGTGTGCTCTTCCGATC*T
D5_221	GTCGCAGT			D7_221	GGAGACCT	AGGTCTCC		AATGATACGGCGACCACCGAGATCTACACGTCGCAGTACACTCTTTCCCTACACGACGCTCTTCCGATC*T		CAAGCAGAAGACGGCATACGAGATAGGTCTCCGTGACTGGAGTTCAGACGTGTGCTCTTCCGATC*T
D5_222	TGAATGTA			D7_222	CGCAAGCC	GGCTTGCG		AATGATACGGCGACCACCGAGATCTACACTGAATGTAACACTCTTTCCCTACACGACGCTCTTCCGATC*T		CAAGCAGAAGACGGCATACGAGATGGCTTGCGGTGACTGGAGTTCAGACGTGTGCTCTTCCGATC*T
D5_223	TTGATAAG			D7_223	TCCGGTCA	TGACCGGA		AATGATACGGCGACCACCGAGATCTACACTTGATAAGACACTCTTTCCCTACACGACGCTCTTCCGATC*T		CAAGCAGAAGACGGCATACGAGATTGACCGGAGTGACTGGAGTTCAGACGTGTGCTCTTCCGATC*T
D5_224	AAACCGAC			D7_224	GTTTAGTC	GACTAAAC		AATGATACGGCGACCACCGAGATCTACACAAACCGACACACTCTTTCCCTACACGACGCTCTTCCGATC*T		CAAGCAGAAGACGGCATACGAGATGACTAAACGTGACTGGAGTTCAGACGTGTGCTCTTCCGATC*T
D5_225	GCACGCCT			D7_225	CTCTTCTA	TAGAAGAG		AATGATACGGCGACCACCGAGATCTACACGCACGCCTACACTCTTTCCCTACACGACGCTCTTCCGATC*T		CAAGCAGAAGACGGCATACGAGATTAGAAGAGGTGACTGGAGTTCAGACGTGTGCTCTTCCGATC*T
D5_226	ATGAACGC			D7_226	GAGAGATT	AATCTCTC		AATGATACGGCGACCACCGAGATCTACACATGAACGCACACTCTTTCCCTACACGACGCTCTTCCGATC*T		CAAGCAGAAGACGGCATACGAGATAATCTCTCGTGACTGGAGTTCAGACGTGTGCTCTTCCGATC*T
D5_227	GTATCTGC			D7_227	GGAAACAG	CTGTTTCC		AATGATACGGCGACCACCGAGATCTACACGTATCTGCACACTCTTTCCCTACACGACGCTCTTCCGATC*T		CAAGCAGAAGACGGCATACGAGATCTGTTTCCGTGACTGGAGTTCAGACGTGTGCTCTTCCGATC*T
D5_228	CCATAATC			D7_228	TCCATTTG	CAAATGGA		AATGATACGGCGACCACCGAGATCTACACCCATAATCACACTCTTTCCCTACACGACGCTCTTCCGATC*T		CAAGCAGAAGACGGCATACGAGATCAAATGGAGTGACTGGAGTTCAGACGTGTGCTCTTCCGATC*T
D5_229	AGATAATA			D7_229	CCTAGGGC	GCCCTAGG		AATGATACGGCGACCACCGAGATCTACACAGATAATAACACTCTTTCCCTACACGACGCTCTTCCGATC*T		CAAGCAGAAGACGGCATACGAGATGCCCTAGGGTGACTGGAGTTCAGACGTGTGCTCTTCCGATC*T
D5_230	TAGATTAA			D7_230	TTCCAAAT	ATTTGGAA		AATGATACGGCGACCACCGAGATCTACACTAGATTAAACACTCTTTCCCTACACGACGCTCTTCCGATC*T		CAAGCAGAAGACGGCATACGAGATATTTGGAAGTGACTGGAGTTCAGACGTGTGCTCTTCCGATC*T
D5_231	CTGGTTCG			D7_231	ACTGCGGC	GCCGCAGT		AATGATACGGCGACCACCGAGATCTACACCTGGTTCGACACTCTTTCCCTACACGACGCTCTTCCGATC*T		CAAGCAGAAGACGGCATACGAGATGCCGCAGTGTGACTGGAGTTCAGACGTGTGCTCTTCCGATC*T
D5_232	CCCGGTGG			D7_232	GGAGGAGA	TCTCCTCC		AATGATACGGCGACCACCGAGATCTACACCCCGGTGGACACTCTTTCCCTACACGACGCTCTTCCGATC*T		CAAGCAGAAGACGGCATACGAGATTCTCCTCCGTGACTGGAGTTCAGACGTGTGCTCTTCCGATC*T
D5_233	ATGCTCGA			D7_233	TTTAGTAA	TTACTAAA		AATGATACGGCGACCACCGAGATCTACACATGCTCGAACACTCTTTCCCTACACGACGCTCTTCCGATC*T		CAAGCAGAAGACGGCATACGAGATTTACTAAAGTGACTGGAGTTCAGACGTGTGCTCTTCCGATC*T
D5_234	TAACTGTC			D7_234	CAAAGCGG	CCGCTTTG		AATGATACGGCGACCACCGAGATCTACACTAACTGTCACACTCTTTCCCTACACGACGCTCTTCCGATC*T		CAAGCAGAAGACGGCATACGAGATCCGCTTTGGTGACTGGAGTTCAGACGTGTGCTCTTCCGATC*T
D5_235	AGCCGTAA			D7_235	AGACGTCT	AGACGTCT		AATGATACGGCGACCACCGAGATCTACACAGCCGTAAACACTCTTTCCCTACACGACGCTCTTCCGATC*T		CAAGCAGAAGACGGCATACGAGATAGACGTCTGTGACTGGAGTTCAGACGTGTGCTCTTCCGATC*T
D5_236	AAGAAACG			D7_236	TGAAATGA	TCATTTCA		AATGATACGGCGACCACCGAGATCTACACAAGAAACGACACTCTTTCCCTACACGACGCTCTTCCGATC*T		CAAGCAGAAGACGGCATACGAGATTCATTTCAGTGACTGGAGTTCAGACGTGTGCTCTTCCGATC*T
D5_237	CATACGAT			D7_237	TTCCGCGC	GCGCGGAA		AATGATACGGCGACCACCGAGATCTACACCATACGATACACTCTTTCCCTACACGACGCTCTTCCGATC*T		CAAGCAGAAGACGGCATACGAGATGCGCGGAAGTGACTGGAGTTCAGACGTGTGCTCTTCCGATC*T
D5_238	GGCCCATA			D7_238	GGGTTGCG	CGCAACCC		AATGATACGGCGACCACCGAGATCTACACGGCCCATAACACTCTTTCCCTACACGACGCTCTTCCGATC*T		CAAGCAGAAGACGGCATACGAGATCGCAACCCGTGACTGGAGTTCAGACGTGTGCTCTTCCGATC*T
D5_239	CCGTGGAG			D7_239	TCCTTAAT	ATTAAGGA		AATGATACGGCGACCACCGAGATCTACACCCGTGGAGACACTCTTTCCCTACACGACGCTCTTCCGATC*T		CAAGCAGAAGACGGCATACGAGATATTAAGGAGTGACTGGAGTTCAGACGTGTGCTCTTCCGATC*T
D5_240	AAACCCGA			D7_240	CAGAAATG	CATTTCTG		AATGATACGGCGACCACCGAGATCTACACAAACCCGAACACTCTTTCCCTACACGACGCTCTTCCGATC*T		CAAGCAGAAGACGGCATACGAGATCATTTCTGGTGACTGGAGTTCAGACGTGTGCTCTTCCGATC*T
D5_241	GCGACACG			D7_241	CTGAACCA	TGGTTCAG		AATGATACGGCGACCACCGAGATCTACACGCGACACGACACTCTTTCCCTACACGACGCTCTTCCGATC*T		CAAGCAGAAGACGGCATACGAGATTGGTTCAGGTGACTGGAGTTCAGACGTGTGCTCTTCCGATC*T
D5_242	ACTCACGG			D7_242	GGTTGCGG	CCGCAACC		AATGATACGGCGACCACCGAGATCTACACACTCACGGACACTCTTTCCCTACACGACGCTCTTCCGATC*T		CAAGCAGAAGACGGCATACGAGATCCGCAACCGTGACTGGAGTTCAGACGTGTGCTCTTCCGATC*T
D5_243	GAACTCCA			D7_243	TGGGCTAT	ATAGCCCA		AATGATACGGCGACCACCGAGATCTACACGAACTCCAACACTCTTTCCCTACACGACGCTCTTCCGATC*T		CAAGCAGAAGACGGCATACGAGATATAGCCCAGTGACTGGAGTTCAGACGTGTGCTCTTCCGATC*T
D5_244	ATCGAAAC			D7_244	GACGCTAA	TTAGCGTC		AATGATACGGCGACCACCGAGATCTACACATCGAAACACACTCTTTCCCTACACGACGCTCTTCCGATC*T		CAAGCAGAAGACGGCATACGAGATTTAGCGTCGTGACTGGAGTTCAGACGTGTGCTCTTCCGATC*T
D5_245	TTGCTTCA			D7_245	GGCTTTAC	GTAAAGCC		AATGATACGGCGACCACCGAGATCTACACTTGCTTCAACACTCTTTCCCTACACGACGCTCTTCCGATC*T		CAAGCAGAAGACGGCATACGAGATGTAAAGCCGTGACTGGAGTTCAGACGTGTGCTCTTCCGATC*T
D5_246	CTCGATGC			D7_246	TGTTCGAT	ATCGAACA		AATGATACGGCGACCACCGAGATCTACACCTCGATGCACACTCTTTCCCTACACGACGCTCTTCCGATC*T		CAAGCAGAAGACGGCATACGAGATATCGAACAGTGACTGGAGTTCAGACGTGTGCTCTTCCGATC*T
D5_247	TAGCGGAG			D7_247	GAGCACGC	GCGTGCTC		AATGATACGGCGACCACCGAGATCTACACTAGCGGAGACACTCTTTCCCTACACGACGCTCTTCCGATC*T		CAAGCAGAAGACGGCATACGAGATGCGTGCTCGTGACTGGAGTTCAGACGTGTGCTCTTCCGATC*T
D5_248	TAACAACT			D7_248	CTTTGAAA	TTTCAAAG		AATGATACGGCGACCACCGAGATCTACACTAACAACTACACTCTTTCCCTACACGACGCTCTTCCGATC*T		CAAGCAGAAGACGGCATACGAGATTTTCAAAGGTGACTGGAGTTCAGACGTGTGCTCTTCCGATC*T
D5_249	ACTAAACA			D7_249	GAGGTCAC	GTGACCTC		AATGATACGGCGACCACCGAGATCTACACACTAAACAACACTCTTTCCCTACACGACGCTCTTCCGATC*T		CAAGCAGAAGACGGCATACGAGATGTGACCTCGTGACTGGAGTTCAGACGTGTGCTCTTCCGATC*T

D5_250	TAGAACTG			D7_250	CCTCACGT	ACGTGAGG		AATGATACGGCGACCACCGAGATCTACACTAGAACTGACACTCTTTCCCTACACGACGCTCTTCCGATC*T		CAAGCAGAAGACGGCATACGAGATACGTGAGGGTGACTGGAGTTCAGACGTGTGCTCTTCCGATC*T
D5_251	GGTACCCG			D7_251	TGGCTGGT	ACCAGCCA		AATGATACGGCGACCACCGAGATCTACACGGTACCCGACACTCTTTCCCTACACGACGCTCTTCCGATC*T		CAAGCAGAAGACGGCATACGAGATACCAGCCAGTGACTGGAGTTCAGACGTGTGCTCTTCCGATC*T
D5_252	TTATAATT			D7_252	GCTCTGAG	CTCAGAGC		AATGATACGGCGACCACCGAGATCTACACTTATAATTACACTCTTTCCCTACACGACGCTCTTCCGATC*T		CAAGCAGAAGACGGCATACGAGATCTCAGAGCGTGACTGGAGTTCAGACGTGTGCTCTTCCGATC*T
D5_253	ACATGCAT			D7_253	CGGCGCGG	CCGCGCCG		AATGATACGGCGACCACCGAGATCTACACACATGCATACACTCTTTCCCTACACGACGCTCTTCCGATC*T		CAAGCAGAAGACGGCATACGAGATCCGCGCCGGTGACTGGAGTTCAGACGTGTGCTCTTCCGATC*T
D5_254	TAGTCACG			D7_254	TCGCATCC	GGATGCGA		AATGATACGGCGACCACCGAGATCTACACTAGTCACGACACTCTTTCCCTACACGACGCTCTTCCGATC*T		CAAGCAGAAGACGGCATACGAGATGGATGCGAGTGACTGGAGTTCAGACGTGTGCTCTTCCGATC*T
D5_255	GTCCACTC			D7_255	GGGCCAGT	ACTGGCCC		AATGATACGGCGACCACCGAGATCTACACGTCCACTCACACTCTTTCCCTACACGACGCTCTTCCGATC*T		CAAGCAGAAGACGGCATACGAGATACTGGCCCGTGACTGGAGTTCAGACGTGTGCTCTTCCGATC*T
D5_256	ACGACCAC			D7_256	CGTCTCGC	GCGAGACG		AATGATACGGCGACCACCGAGATCTACACACGACCACACACTCTTTCCCTACACGACGCTCTTCCGATC*T		CAAGCAGAAGACGGCATACGAGATGCGAGACGGTGACTGGAGTTCAGACGTGTGCTCTTCCGATC*T
D5_257	TTCCGTCT			D7_257	GTGGCACT	AGTGCCAC		AATGATACGGCGACCACCGAGATCTACACTTCCGTCTACACTCTTTCCCTACACGACGCTCTTCCGATC*T		CAAGCAGAAGACGGCATACGAGATAGTGCCACGTGACTGGAGTTCAGACGTGTGCTCTTCCGATC*T
D5_258	AGGATATC			D7_258	TCGCTTGG	CCAAGCGA		AATGATACGGCGACCACCGAGATCTACACAGGATATCACACTCTTTCCCTACACGACGCTCTTCCGATC*T		CAAGCAGAAGACGGCATACGAGATCCAAGCGAGTGACTGGAGTTCAGACGTGTGCTCTTCCGATC*T
D5_259	TCCCGAGA			D7_259	TTGCGACA	TGTCGCAA		AATGATACGGCGACCACCGAGATCTACACTCCCGAGAACACTCTTTCCCTACACGACGCTCTTCCGATC*T		CAAGCAGAAGACGGCATACGAGATTGTCGCAAGTGACTGGAGTTCAGACGTGTGCTCTTCCGATC*T
D5_260	AAAGATCA			D7_260	GGCCGCGT	ACGCGGCC		AATGATACGGCGACCACCGAGATCTACACAAAGATCAACACTCTTTCCCTACACGACGCTCTTCCGATC*T		CAAGCAGAAGACGGCATACGAGATACGCGGCCGTGACTGGAGTTCAGACGTGTGCTCTTCCGATC*T
D5_261	GGCTGCCA			D7_261	AACCGGGT	ACCCGGTT		AATGATACGGCGACCACCGAGATCTACACGGCTGCCAACACTCTTTCCCTACACGACGCTCTTCCGATC*T		CAAGCAGAAGACGGCATACGAGATACCCGGTTGTGACTGGAGTTCAGACGTGTGCTCTTCCGATC*T
D5_262	TCCTATTG			D7_262	TGTTTCTA	TAGAAACA		AATGATACGGCGACCACCGAGATCTACACTCCTATTGACACTCTTTCCCTACACGACGCTCTTCCGATC*T		CAAGCAGAAGACGGCATACGAGATTAGAAACAGTGACTGGAGTTCAGACGTGTGCTCTTCCGATC*T
D5_263	AGGAAAGT			D7_263	GCTAGAAG	CTTCTAGC		AATGATACGGCGACCACCGAGATCTACACAGGAAAGTACACTCTTTCCCTACACGACGCTCTTCCGATC*T		CAAGCAGAAGACGGCATACGAGATCTTCTAGCGTGACTGGAGTTCAGACGTGTGCTCTTCCGATC*T
D5_264	CAGAGGAC			D7_264	TTATTGCT	AGCAATAA		AATGATACGGCGACCACCGAGATCTACACCAGAGGACACACTCTTTCCCTACACGACGCTCTTCCGATC*T		CAAGCAGAAGACGGCATACGAGATAGCAATAAGTGACTGGAGTTCAGACGTGTGCTCTTCCGATC*T
D5_265	TGCCTATC			D7_265	GTGTGGAC	GTCCACAC		AATGATACGGCGACCACCGAGATCTACACTGCCTATCACACTCTTTCCCTACACGACGCTCTTCCGATC*T		CAAGCAGAAGACGGCATACGAGATGTCCACACGTGACTGGAGTTCAGACGTGTGCTCTTCCGATC*T
D5_266	CTGCTTTC			D7_266	GGGTGATC	GATCACCC		AATGATACGGCGACCACCGAGATCTACACCTGCTTTCACACTCTTTCCCTACACGACGCTCTTCCGATC*T		CAAGCAGAAGACGGCATACGAGATGATCACCCGTGACTGGAGTTCAGACGTGTGCTCTTCCGATC*T
D5_267	CTCTTCGT			D7_267	CCAAATGG	CCATTTGG		AATGATACGGCGACCACCGAGATCTACACCTCTTCGTACACTCTTTCCCTACACGACGCTCTTCCGATC*T		CAAGCAGAAGACGGCATACGAGATCCATTTGGGTGACTGGAGTTCAGACGTGTGCTCTTCCGATC*T
D5_268	TAGCGCCA			D7_268	TCTATATT	AATATAGA		AATGATACGGCGACCACCGAGATCTACACTAGCGCCAACACTCTTTCCCTACACGACGCTCTTCCGATC*T		CAAGCAGAAGACGGCATACGAGATAATATAGAGTGACTGGAGTTCAGACGTGTGCTCTTCCGATC*T
D5_269	TATGAGAC			D7_269	CCTGCCAG	CTGGCAGG		AATGATACGGCGACCACCGAGATCTACACTATGAGACACACTCTTTCCCTACACGACGCTCTTCCGATC*T		CAAGCAGAAGACGGCATACGAGATCTGGCAGGGTGACTGGAGTTCAGACGTGTGCTCTTCCGATC*T
D5_270	ATTTATTA			D7_270	CTGTATGT	ACATACAG		AATGATACGGCGACCACCGAGATCTACACATTTATTAACACTCTTTCCCTACACGACGCTCTTCCGATC*T		CAAGCAGAAGACGGCATACGAGATACATACAGGTGACTGGAGTTCAGACGTGTGCTCTTCCGATC*T
D5_271	GCGGAATT			D7_271	CATAAGTA	TACTTATG		AATGATACGGCGACCACCGAGATCTACACGCGGAATTACACTCTTTCCCTACACGACGCTCTTCCGATC*T		CAAGCAGAAGACGGCATACGAGATTACTTATGGTGACTGGAGTTCAGACGTGTGCTCTTCCGATC*T
D5_272	TCGCGTGG			D7_272	GGCATACG	CGTATGCC		AATGATACGGCGACCACCGAGATCTACACTCGCGTGGACACTCTTTCCCTACACGACGCTCTTCCGATC*T		CAAGCAGAAGACGGCATACGAGATCGTATGCCGTGACTGGAGTTCAGACGTGTGCTCTTCCGATC*T
D5_273	ATTAATCT			D7_273	GTTTCTAA	TTAGAAAC		AATGATACGGCGACCACCGAGATCTACACATTAATCTACACTCTTTCCCTACACGACGCTCTTCCGATC*T		CAAGCAGAAGACGGCATACGAGATTTAGAAACGTGACTGGAGTTCAGACGTGTGCTCTTCCGATC*T
D5_274	GGCGAGGA			D7_274	CATCGGAA	TTCCGATG		AATGATACGGCGACCACCGAGATCTACACGGCGAGGAACACTCTTTCCCTACACGACGCTCTTCCGATC*T		CAAGCAGAAGACGGCATACGAGATTTCCGATGGTGACTGGAGTTCAGACGTGTGCTCTTCCGATC*T
D5_275	AGCGTGAC			D7_275	AGACTTGG	CCAAGTCT		AATGATACGGCGACCACCGAGATCTACACAGCGTGACACACTCTTTCCCTACACGACGCTCTTCCGATC*T		CAAGCAGAAGACGGCATACGAGATCCAAGTCTGTGACTGGAGTTCAGACGTGTGCTCTTCCGATC*T
D5_276	TACTCTCC			D7_276	GACGGTTT	AAACCGTC		AATGATACGGCGACCACCGAGATCTACACTACTCTCCACACTCTTTCCCTACACGACGCTCTTCCGATC*T		CAAGCAGAAGACGGCATACGAGATAAACCGTCGTGACTGGAGTTCAGACGTGTGCTCTTCCGATC*T
D5_277	ATCAGTTG			D7_277	TAGGGATA	TATCCCTA		AATGATACGGCGACCACCGAGATCTACACATCAGTTGACACTCTTTCCCTACACGACGCTCTTCCGATC*T		CAAGCAGAAGACGGCATACGAGATTATCCCTAGTGACTGGAGTTCAGACGTGTGCTCTTCCGATC*T
D5_278	TACAGAAG			D7_278	TTTCGTCC	GGACGAAA		AATGATACGGCGACCACCGAGATCTACACTACAGAAGACACTCTTTCCCTACACGACGCTCTTCCGATC*T		CAAGCAGAAGACGGCATACGAGATGGACGAAAGTGACTGGAGTTCAGACGTGTGCTCTTCCGATC*T
D5_279	AAACGTTC			D7_279	CTAGTTGT	ACAACTAG		AATGATACGGCGACCACCGAGATCTACACAAACGTTCACACTCTTTCCCTACACGACGCTCTTCCGATC*T		CAAGCAGAAGACGGCATACGAGATACAACTAGGTGACTGGAGTTCAGACGTGTGCTCTTCCGATC*T
D5_280	TAAGTACC			D7_280	GGGAGCTG	CAGCTCCC		AATGATACGGCGACCACCGAGATCTACACTAAGTACCACACTCTTTCCCTACACGACGCTCTTCCGATC*T		CAAGCAGAAGACGGCATACGAGATCAGCTCCCGTGACTGGAGTTCAGACGTGTGCTCTTCCGATC*T
D5_281	GTAATTTA			D7_281	TGCACCAG	CTGGTGCA		AATGATACGGCGACCACCGAGATCTACACGTAATTTAACACTCTTTCCCTACACGACGCTCTTCCGATC*T		CAAGCAGAAGACGGCATACGAGATCTGGTGCAGTGACTGGAGTTCAGACGTGTGCTCTTCCGATC*T
D5_282	TCGCCTTC			D7_282	CAAAGACT	AGTCTTTG		AATGATACGGCGACCACCGAGATCTACACTCGCCTTCACACTCTTTCCCTACACGACGCTCTTCCGATC*T		CAAGCAGAAGACGGCATACGAGATAGTCTTTGGTGACTGGAGTTCAGACGTGTGCTCTTCCGATC*T
D5_283	AGCACTTA			D7_283	GTAGTCAA	TTGACTAC		AATGATACGGCGACCACCGAGATCTACACAGCACTTAACACTCTTTCCCTACACGACGCTCTTCCGATC*T		CAAGCAGAAGACGGCATACGAGATTTGACTACGTGACTGGAGTTCAGACGTGTGCTCTTCCGATC*T
D5_284	CCTCGCCA			D7_284	GCCTCTCG	CGAGAGGC		AATGATACGGCGACCACCGAGATCTACACCCTCGCCAACACTCTTTCCCTACACGACGCTCTTCCGATC*T		CAAGCAGAAGACGGCATACGAGATCGAGAGGCGTGACTGGAGTTCAGACGTGTGCTCTTCCGATC*T
D5_285	CCCAAATG			D7_285	TGGTCGGA	TCCGACCA		AATGATACGGCGACCACCGAGATCTACACCCCAAATGACACTCTTTCCCTACACGACGCTCTTCCGATC*T		CAAGCAGAAGACGGCATACGAGATTCCGACCAGTGACTGGAGTTCAGACGTGTGCTCTTCCGATC*T
D5_286	GATCTGTG			D7_286	CGTTGTAT	ATACAACG		AATGATACGGCGACCACCGAGATCTACACGATCTGTGACACTCTTTCCCTACACGACGCTCTTCCGATC*T		CAAGCAGAAGACGGCATACGAGATATACAACGGTGACTGGAGTTCAGACGTGTGCTCTTCCGATC*T
D5_287	AGCAGCAG			D7_287	TTGTCGCC	GGCGACAA		AATGATACGGCGACCACCGAGATCTACACAGCAGCAGACACTCTTTCCCTACACGACGCTCTTCCGATC*T		CAAGCAGAAGACGGCATACGAGATGGCGACAAGTGACTGGAGTTCAGACGTGTGCTCTTCCGATC*T
D5_288	TCTTGCAA			D7_288	CTGCCTGC	GCAGGCAG		AATGATACGGCGACCACCGAGATCTACACTCTTGCAAACACTCTTTCCCTACACGACGCTCTTCCGATC*T		CAAGCAGAAGACGGCATACGAGATGCAGGCAGGTGACTGGAGTTCAGACGTGTGCTCTTCCGATC*T

SetD

i5 barcodes

i7 barcodes

	**Sequence For Oligo**				**Sequence Obtained**	**Sequence for oligo**		i5 oligo to order		i7 oligo to order
D5_289	TGAGCCGC			D7_289	CAGTTCCT	AGGAACTG		AATGATACGGCGACCACCGAGATCTACACTGAGCCGCACACTCTTTCCCTACACGACGCTCTTCCGATC*T		CAAGCAGAAGACGGCATACGAGATAGGAACTGGTGACTGGAGTTCAGACGTGTGCTCTTCCGATC*T
D5_290	ACCGTTCC			D7_290	AGATGAAC	GTTCATCT		AATGATACGGCGACCACCGAGATCTACACACCGTTCCACACTCTTTCCCTACACGACGCTCTTCCGATC*T		CAAGCAGAAGACGGCATACGAGATGTTCATCTGTGACTGGAGTTCAGACGTGTGCTCTTCCGATC*T
D5_291	ATTATGTA			D7_291	GTTCGCAA	TTGCGAAC		AATGATACGGCGACCACCGAGATCTACACATTATGTAACACTCTTTCCCTACACGACGCTCTTCCGATC*T		CAAGCAGAAGACGGCATACGAGATTTGCGAACGTGACTGGAGTTCAGACGTGTGCTCTTCCGATC*T
D5_292	TAAAGGCT			D7_292	CTCATATG	CATATGAG		AATGATACGGCGACCACCGAGATCTACACTAAAGGCTACACTCTTTCCCTACACGACGCTCTTCCGATC*T		CAAGCAGAAGACGGCATACGAGATCATATGAGGTGACTGGAGTTCAGACGTGTGCTCTTCCGATC*T
D5_293	ATCATCAC			D7_293	GGTTTGTT	AACAAACC		AATGATACGGCGACCACCGAGATCTACACATCATCACACACTCTTTCCCTACACGACGCTCTTCCGATC*T		CAAGCAGAAGACGGCATACGAGATAACAAACCGTGACTGGAGTTCAGACGTGTGCTCTTCCGATC*T
D5_294	ACCACAAA			D7_294	GTCGATCG	CGATCGAC		AATGATACGGCGACCACCGAGATCTACACACCACAAAACACTCTTTCCCTACACGACGCTCTTCCGATC*T		CAAGCAGAAGACGGCATACGAGATCGATCGACGTGACTGGAGTTCAGACGTGTGCTCTTCCGATC*T
D5_295	TACGATAA			D7_295	GAGAACAA	TTGTTCTC		AATGATACGGCGACCACCGAGATCTACACTACGATAAACACTCTTTCCCTACACGACGCTCTTCCGATC*T		CAAGCAGAAGACGGCATACGAGATTTGTTCTCGTGACTGGAGTTCAGACGTGTGCTCTTCCGATC*T
D5_296	ACTCTTCT			D7_296	GGGTGTAG	CTACACCC		AATGATACGGCGACCACCGAGATCTACACACTCTTCTACACTCTTTCCCTACACGACGCTCTTCCGATC*T		CAAGCAGAAGACGGCATACGAGATCTACACCCGTGACTGGAGTTCAGACGTGTGCTCTTCCGATC*T
D5_297	CAGCCCTC			D7_297	GACTATGA	TCATAGTC		AATGATACGGCGACCACCGAGATCTACACCAGCCCTCACACTCTTTCCCTACACGACGCTCTTCCGATC*T		CAAGCAGAAGACGGCATACGAGATTCATAGTCGTGACTGGAGTTCAGACGTGTGCTCTTCCGATC*T
D5_298	GAAGGCGT			D7_298	CGTCTGAT	ATCAGACG		AATGATACGGCGACCACCGAGATCTACACGAAGGCGTACACTCTTTCCCTACACGACGCTCTTCCGATC*T		CAAGCAGAAGACGGCATACGAGATATCAGACGGTGACTGGAGTTCAGACGTGTGCTCTTCCGATC*T
D5_299	CTAAATGG			D7_299	GTCAATAA	TTATTGAC		AATGATACGGCGACCACCGAGATCTACACCTAAATGGACACTCTTTCCCTACACGACGCTCTTCCGATC*T		CAAGCAGAAGACGGCATACGAGATTTATTGACGTGACTGGAGTTCAGACGTGTGCTCTTCCGATC*T
D5_300	TACGTGAG			D7_300	TCGGCGAA	TTCGCCGA		AATGATACGGCGACCACCGAGATCTACACTACGTGAGACACTCTTTCCCTACACGACGCTCTTCCGATC*T		CAAGCAGAAGACGGCATACGAGATTTCGCCGAGTGACTGGAGTTCAGACGTGTGCTCTTCCGATC*T
D5_301	CATGTACA			D7_301	TTCCTGGG	CCCAGGAA		AATGATACGGCGACCACCGAGATCTACACCATGTACAACACTCTTTCCCTACACGACGCTCTTCCGATC*T		CAAGCAGAAGACGGCATACGAGATCCCAGGAAGTGACTGGAGTTCAGACGTGTGCTCTTCCGATC*T
D5_302	TCGCAACA			D7_302	GGCTAGCT	AGCTAGCC		AATGATACGGCGACCACCGAGATCTACACTCGCAACAACACTCTTTCCCTACACGACGCTCTTCCGATC*T		CAAGCAGAAGACGGCATACGAGATAGCTAGCCGTGACTGGAGTTCAGACGTGTGCTCTTCCGATC*T
D5_303	GCATGATA			D7_303	CTCCAGGC	GCCTGGAG		AATGATACGGCGACCACCGAGATCTACACGCATGATAACACTCTTTCCCTACACGACGCTCTTCCGATC*T		CAAGCAGAAGACGGCATACGAGATGCCTGGAGGTGACTGGAGTTCAGACGTGTGCTCTTCCGATC*T
D5_304	ACAAGGAC			D7_304	GATTAGGT	ACCTAATC		AATGATACGGCGACCACCGAGATCTACACACAAGGACACACTCTTTCCCTACACGACGCTCTTCCGATC*T		CAAGCAGAAGACGGCATACGAGATACCTAATCGTGACTGGAGTTCAGACGTGTGCTCTTCCGATC*T

D5_305	GATGCCGG			D7_305	ACGCCGGG	CCCGGCGT		AATGATACGGCGACCACCGAGATCTACACGATGCCGGACACTCTTTCCCTACACGACGCTCTTCCGATC*T	CAAGCAGAAGACGGCATACGAGATCCCGGCGTGTGACTGGAGTTCAGACGTGTGCTCTTCCGATC*T
D5_306	TCCGACTT			D7_306	TCCTATCT	AGATAGGA		AATGATACGGCGACCACCGAGATCTACACTCCGACTTACACTCTTTCCCTACACGACGCTCTTCCGATC*T	CAAGCAGAAGACGGCATACGAGATAGATAGGAGTGACTGGAGTTCAGACGTGTGCTCTTCCGATC*T
D5_307	CCCATGAC			D7_307	TTTGCTCG	CGAGCAAA		AATGATACGGCGACCACCGAGATCTACACCCCATGACACACTCTTTCCCTACACGACGCTCTTCCGATC*T	CAAGCAGAAGACGGCATACGAGATCGAGCAAAGTGACTGGAGTTCAGACGTGTGCTCTTCCGATC*T
D5_308	TCGATCCA			D7_308	TACGGTAG	CTACCGTA		AATGATACGGCGACCACCGAGATCTACACTCGATCCAACACTCTTTCCCTACACGACGCTCTTCCGATC*T	CAAGCAGAAGACGGCATACGAGATCTACCGTAGTGACTGGAGTTCAGACGTGTGCTCTTCCGATC*T
D5_309	CTCCCAGC			D7_309	GCCATTCC	GGAATGGC		AATGATACGGCGACCACCGAGATCTACACCTCCCAGCACACTCTTTCCCTACACGACGCTCTTCCGATC*T	CAAGCAGAAGACGGCATACGAGATGGAATGGCGTGACTGGAGTTCAGACGTGTGCTCTTCCGATC*T
D5_310	ATGGTTTA			D7_310	TGGGTTTG	CAAACCCA		AATGATACGGCGACCACCGAGATCTACACATGGTTTAACACTCTTTCCCTACACGACGCTCTTCCGATC*T	CAAGCAGAAGACGGCATACGAGATCAAACCCAGTGACTGGAGTTCAGACGTGTGCTCTTCCGATC*T
D5_311	CAGGTAGG			D7_311	AGATCGTC	GACGATCT		AATGATACGGCGACCACCGAGATCTACACCAGGTAGGACACTCTTTCCCTACACGACGCTCTTCCGATC*T	CAAGCAGAAGACGGCATACGAGATGACGATCTGTGACTGGAGTTCAGACGTGTGCTCTTCCGATC*T
D5_312	TGACCCTA			D7_312	GCGTACGA	TCGTACGC		AATGATACGGCGACCACCGAGATCTACACTGACCCTAACACTCTTTCCCTACACGACGCTCTTCCGATC*T	CAAGCAGAAGACGGCATACGAGATTCGTACGCGTGACTGGAGTTCAGACGTGTGCTCTTCCGATC*T
D5_313	TGAAATAC			D7_313	CCATCTGC	GCAGATGG		AATGATACGGCGACCACCGAGATCTACACTGAAATACACACTCTTTCCCTACACGACGCTCTTCCGATC*T	CAAGCAGAAGACGGCATACGAGATGCAGATGGGTGACTGGAGTTCAGACGTGTGCTCTTCCGATC*T
D5_314	AGAGGACT			D7_314	GTCGGATC	GATCCGAC		AATGATACGGCGACCACCGAGATCTACACAGAGGACTACACTCTTTCCCTACACGACGCTCTTCCGATC*T	CAAGCAGAAGACGGCATACGAGATGATCCGACGTGACTGGAGTTCAGACGTGTGCTCTTCCGATC*T
D5_315	TCACCTGT			D7_315	TGCAAAGT	ACTTTGCA		AATGATACGGCGACCACCGAGATCTACACTCACCTGTACACTCTTTCCCTACACGACGCTCTTCCGATC*T	CAAGCAGAAGACGGCATACGAGATACTTTGCAGTGACTGGAGTTCAGACGTGTGCTCTTCCGATC*T
D5_316	TCCTTACA			D7_316	TTCAAGAG	CTCTTGAA		AATGATACGGCGACCACCGAGATCTACACTCCTTACAACACTCTTTCCCTACACGACGCTCTTCCGATC*T	CAAGCAGAAGACGGCATACGAGATCTCTTGAAGTGACTGGAGTTCAGACGTGTGCTCTTCCGATC*T
D5_317	ATAGGTCC			D7_317	CGCGATAG	CTATCGCG		AATGATACGGCGACCACCGAGATCTACACATAGGTCCACACTCTTTCCCTACACGACGCTCTTCCGATC*T	CAAGCAGAAGACGGCATACGAGATCTATCGCGGTGACTGGAGTTCAGACGTGTGCTCTTCCGATC*T
D5_318	GATGAAGT			D7_318	GGCGGCTA	TAGCCGCC		AATGATACGGCGACCACCGAGATCTACACGATGAAGTACACTCTTTCCCTACACGACGCTCTTCCGATC*T	CAAGCAGAAGACGGCATACGAGATTAGCCGCCGTGACTGGAGTTCAGACGTGTGCTCTTCCGATC*T
D5_319	TTCCACCA			D7_319	GTTTCCGC	GCGGAAAC		AATGATACGGCGACCACCGAGATCTACACTTCCACCAACACTCTTTCCCTACACGACGCTCTTCCGATC*T	CAAGCAGAAGACGGCATACGAGATGCGGAAACGTGACTGGAGTTCAGACGTGTGCTCTTCCGATC*T
D5_320	ACAGAGCT			D7_320	CTAGATAA	TTATCTAG		AATGATACGGCGACCACCGAGATCTACACACAGAGCTACACTCTTTCCCTACACGACGCTCTTCCGATC*T	CAAGCAGAAGACGGCATACGAGATTTATCTAGGTGACTGGAGTTCAGACGTGTGCTCTTCCGATC*T
D5_321	CGCCCGTC			D7_321	CCGGGAAT	ATTCCCGG		AATGATACGGCGACCACCGAGATCTACACCGCCCGTCACACTCTTTCCCTACACGACGCTCTTCCGATC*T	CAAGCAGAAGACGGCATACGAGATATTCCCGGGTGACTGGAGTTCAGACGTGTGCTCTTCCGATC*T
D5_322	ATCTGAAG			D7_322	ACGTTTGC	GCAAACGT		AATGATACGGCGACCACCGAGATCTACACATCTGAAGACACTCTTTCCCTACACGACGCTCTTCCGATC*T	CAAGCAGAAGACGGCATACGAGATGCAAACGTGTGACTGGAGTTCAGACGTGTGCTCTTCCGATC*T
D5_323	CTAGGTGT			D7_323	CGGTATCC	GGATACCG		AATGATACGGCGACCACCGAGATCTACACCTAGGTGTACACTCTTTCCCTACACGACGCTCTTCCGATC*T	CAAGCAGAAGACGGCATACGAGATGGATACCGGTGACTGGAGTTCAGACGTGTGCTCTTCCGATC*T
D5_324	CGATTGAA			D7_324	GTCGTTTA	TAAACGAC		AATGATACGGCGACCACCGAGATCTACACCGATTGAAACACTCTTTCCCTACACGACGCTCTTCCGATC*T	CAAGCAGAAGACGGCATACGAGATTAAACGACGTGACTGGAGTTCAGACGTGTGCTCTTCCGATC*T
D5_325	TATGTTTC			D7_325	TGCTCACC	GGTGAGCA		AATGATACGGCGACCACCGAGATCTACACTATGTTTCACACTCTTTCCCTACACGACGCTCTTCCGATC*T	CAAGCAGAAGACGGCATACGAGATGGTGAGCAGTGACTGGAGTTCAGACGTGTGCTCTTCCGATC*T
D5_326	AGAAAGGA			D7_326	TGTCGGGA	TCCCGACA		AATGATACGGCGACCACCGAGATCTACACAGAAAGGAACACTCTTTCCCTACACGACGCTCTTCCGATC*T	CAAGCAGAAGACGGCATACGAGATTCCCGACAGTGACTGGAGTTCAGACGTGTGCTCTTCCGATC*T
D5_327	TGACTTAT			D7_327	CTTTACTT	AAGTAAAG		AATGATACGGCGACCACCGAGATCTACACTGACTTATACACTCTTTCCCTACACGACGCTCTTCCGATC*T	CAAGCAGAAGACGGCATACGAGATAAGTAAAGGTGACTGGAGTTCAGACGTGTGCTCTTCCGATC*T
D5_328	TCGTCGCC			D7_328	AACCAACT	AGTTGGTT		AATGATACGGCGACCACCGAGATCTACACTCGTCGCCACACTCTTTCCCTACACGACGCTCTTCCGATC*T	CAAGCAGAAGACGGCATACGAGATAGTTGGTTGTGACTGGAGTTCAGACGTGTGCTCTTCCGATC*T
D5_329	AGTGATAA			D7_329	GTTGTTGC	GCAACAAC		AATGATACGGCGACCACCGAGATCTACACAGTGATAAACACTCTTTCCCTACACGACGCTCTTCCGATC*T	CAAGCAGAAGACGGCATACGAGATGCAACAACGTGACTGGAGTTCAGACGTGTGCTCTTCCGATC*T
D5_330	CGGTCCGC			D7_330	CCAAAGTC	GACTTTGG		AATGATACGGCGACCACCGAGATCTACACCGGTCCGCACACTCTTTCCCTACACGACGCTCTTCCGATC*T	CAAGCAGAAGACGGCATACGAGATGACTTTGGGTGACTGGAGTTCAGACGTGTGCTCTTCCGATC*T
D5_331	TACTAAAC			D7_331	GCCTTTGT	ACAAAGGC		AATGATACGGCGACCACCGAGATCTACACTACTAAACACACTCTTTCCCTACACGACGCTCTTCCGATC*T	CAAGCAGAAGACGGCATACGAGATACAAAGGCGTGACTGGAGTTCAGACGTGTGCTCTTCCGATC*T
D5_332	GAATGCAA			D7_332	CAGGCTTT	AAAGCCTG		AATGATACGGCGACCACCGAGATCTACACGAATGCAAACACTCTTTCCCTACACGACGCTCTTCCGATC*T	CAAGCAGAAGACGGCATACGAGATAAAGCCTGGTGACTGGAGTTCAGACGTGTGCTCTTCCGATC*T
D5_333	TCGCCCGA			D7_333	GCGGGTTA	TAACCCGC		AATGATACGGCGACCACCGAGATCTACACTCGCCCGAACACTCTTTCCCTACACGACGCTCTTCCGATC*T	CAAGCAGAAGACGGCATACGAGATTAACCCGCGTGACTGGAGTTCAGACGTGTGCTCTTCCGATC*T
D5_334	GACAGAGA			D7_334	CCTGCACA	TGTGCAGG		AATGATACGGCGACCACCGAGATCTACACGACAGAGAACACTCTTTCCCTACACGACGCTCTTCCGATC*T	CAAGCAGAAGACGGCATACGAGATTGTGCAGGGTGACTGGAGTTCAGACGTGTGCTCTTCCGATC*T
D5_335	TAATTCCT			D7_335	GGCCGTTG	CAACGGCC		AATGATACGGCGACCACCGAGATCTACACTAATTCCTACACTCTTTCCCTACACGACGCTCTTCCGATC*T	CAAGCAGAAGACGGCATACGAGATCAACGGCCGTGACTGGAGTTCAGACGTGTGCTCTTCCGATC*T
D5_336	AGCTTGGT			D7_336	CAAATCAA	TTGATTTG		AATGATACGGCGACCACCGAGATCTACACAGCTTGGTACACTCTTTCCCTACACGACGCTCTTCCGATC*T	CAAGCAGAAGACGGCATACGAGATTTGATTTGGTGACTGGAGTTCAGACGTGTGCTCTTCCGATC*T
D5_337	GCTCTTTA			D7_337	CAATACGT	ACGTATTG		AATGATACGGCGACCACCGAGATCTACACGCTCTTTAACACTCTTTCCCTACACGACGCTCTTCCGATC*T	CAAGCAGAAGACGGCATACGAGATACGTATTGGTGACTGGAGTTCAGACGTGTGCTCTTCCGATC*T
D5_338	CCCTATAC			D7_338	GTCCGGTA	TACCGGAC		AATGATACGGCGACCACCGAGATCTACACCCCTATACACACTCTTTCCCTACACGACGCTCTTCCGATC*T	CAAGCAGAAGACGGCATACGAGATTACCGGACGTGACTGGAGTTCAGACGTGTGCTCTTCCGATC*T
D5_339	TCTCTAAA			D7_339	CCGGATTC	GAATCCGG		AATGATACGGCGACCACCGAGATCTACACTCTCTAAAACACTCTTTCCCTACACGACGCTCTTCCGATC*T	CAAGCAGAAGACGGCATACGAGATGAATCCGGGTGACTGGAGTTCAGACGTGTGCTCTTCCGATC*T
D5_340	ATGTAAGG			D7_340	TTCGGGAT	ATCCCGAA		AATGATACGGCGACCACCGAGATCTACACATGTAAGGACACTCTTTCCCTACACGACGCTCTTCCGATC*T	CAAGCAGAAGACGGCATACGAGATATCCCGAAGTGACTGGAGTTCAGACGTGTGCTCTTCCGATC*T
D5_341	GCTTATAT			D7_341	TTTAATCT	AGATTAAA		AATGATACGGCGACCACCGAGATCTACACGCTTATATACACTCTTTCCCTACACGACGCTCTTCCGATC*T	CAAGCAGAAGACGGCATACGAGATAGATTAAAGTGACTGGAGTTCAGACGTGTGCTCTTCCGATC*T
D5_342	CGATTAGT			D7_342	AACGGTGA	TCACCGTT		AATGATACGGCGACCACCGAGATCTACACCGATTAGTACACTCTTTCCCTACACGACGCTCTTCCGATC*T	CAAGCAGAAGACGGCATACGAGATTCACCGTTGTGACTGGAGTTCAGACGTGTGCTCTTCCGATC*T
D5_343	TACCTTTG			D7_343	GGAGGGCG	CGCCCTCC		AATGATACGGCGACCACCGAGATCTACACTACCTTTGACACTCTTTCCCTACACGACGCTCTTCCGATC*T	CAAGCAGAAGACGGCATACGAGATCGCCCTCCGTGACTGGAGTTCAGACGTGTGCTCTTCCGATC*T
D5_344	ATACTCCT			D7_344	TTTGTATC	GATACAAA		AATGATACGGCGACCACCGAGATCTACACATACTCCTACACTCTTTCCCTACACGACGCTCTTCCGATC*T	CAAGCAGAAGACGGCATACGAGATGATACAAAGTGACTGGAGTTCAGACGTGTGCTCTTCCGATC*T
D5_345	TATTACGG			D7_345	TCTTTCCT	AGGAAAGA		AATGATACGGCGACCACCGAGATCTACACTATTACGGACACTCTTTCCCTACACGACGCTCTTCCGATC*T	CAAGCAGAAGACGGCATACGAGATAGGAAAGAGTGACTGGAGTTCAGACGTGTGCTCTTCCGATC*T
D5_346	GATTATCG			D7_346	TGGCCACG	CGTGGCCA		AATGATACGGCGACCACCGAGATCTACACGATTATCGACACTCTTTCCCTACACGACGCTCTTCCGATC*T	CAAGCAGAAGACGGCATACGAGATCGTGGCCAGTGACTGGAGTTCAGACGTGTGCTCTTCCGATC*T
D5_347	TCAAGAAA			D7_347	TCTGCTTT	AAAGCAGA		AATGATACGGCGACCACCGAGATCTACACTCAAGAAAACACTCTTTCCCTACACGACGCTCTTCCGATC*T	CAAGCAGAAGACGGCATACGAGATAAAGCAGAGTGACTGGAGTTCAGACGTGTGCTCTTCCGATC*T
D5_348	CGTTCTCT			D7_348	TCGGGTAC	GTACCCGA		AATGATACGGCGACCACCGAGATCTACACCGTTCTCTACACTCTTTCCCTACACGACGCTCTTCCGATC*T	CAAGCAGAAGACGGCATACGAGATGTACCCGAGTGACTGGAGTTCAGACGTGTGCTCTTCCGATC*T
D5_349	CCTGGAAA			D7_349	TGTGGAAG	CTTCCACA		AATGATACGGCGACCACCGAGATCTACACCCTGGAAAACACTCTTTCCCTACACGACGCTCTTCCGATC*T	CAAGCAGAAGACGGCATACGAGATCTTCCACAGTGACTGGAGTTCAGACGTGTGCTCTTCCGATC*T
D5_350	TATGGTCG			D7_350	CGACGCAA	TTGCGTCG		AATGATACGGCGACCACCGAGATCTACACTATGGTCGACACTCTTTCCCTACACGACGCTCTTCCGATC*T	CAAGCAGAAGACGGCATACGAGATTTGCGTCGGTGACTGGAGTTCAGACGTGTGCTCTTCCGATC*T
D5_351	CGGTATCA			D7_351	TTAAGGGC	GCCCTTAA		AATGATACGGCGACCACCGAGATCTACACCGGTATCAACACTCTTTCCCTACACGACGCTCTTCCGATC*T	CAAGCAGAAGACGGCATACGAGATGCCCTTAAGTGACTGGAGTTCAGACGTGTGCTCTTCCGATC*T
D5_352	ATTGAGAG			D7_352	CCTTCTCT	AGAGAAGG		AATGATACGGCGACCACCGAGATCTACACATTGAGAGACACTCTTTCCCTACACGACGCTCTTCCGATC*T	CAAGCAGAAGACGGCATACGAGATAGAGAAGGGTGACTGGAGTTCAGACGTGTGCTCTTCCGATC*T
D5_353	CCCAATCA			D7_353	TTTGAGGG	CCCTCAAA		AATGATACGGCGACCACCGAGATCTACACCCCAATCAACACTCTTTCCCTACACGACGCTCTTCCGATC*T	CAAGCAGAAGACGGCATACGAGATCCCTCAAAGTGACTGGAGTTCAGACGTGTGCTCTTCCGATC*T
D5_354	TTGCAATC			D7_354	GGCCTGGC	GCCAGGCC		AATGATACGGCGACCACCGAGATCTACACTTGCAATCACACTCTTTCCCTACACGACGCTCTTCCGATC*T	CAAGCAGAAGACGGCATACGAGATGCCAGGCCGTGACTGGAGTTCAGACGTGTGCTCTTCCGATC*T
D5_355	ATGGCAGC			D7_355	AAAGTGAA	TTCACTTT		AATGATACGGCGACCACCGAGATCTACACATGGCAGCACACTCTTTCCCTACACGACGCTCTTCCGATC*T	CAAGCAGAAGACGGCATACGAGATTTCACTTTGTGACTGGAGTTCAGACGTGTGCTCTTCCGATC*T
D5_356	CACGCGAC			D7_356	ACTCGAAA	TTTCGAGT		AATGATACGGCGACCACCGAGATCTACACCACGCGACACACTCTTTCCCTACACGACGCTCTTCCGATC*T	CAAGCAGAAGACGGCATACGAGATTTTCGAGTGTGACTGGAGTTCAGACGTGTGCTCTTCCGATC*T
D5_357	ACCCAGAA			D7_357	CGATTGCA	TGCAATCG		AATGATACGGCGACCACCGAGATCTACACACCCAGAAACACTCTTTCCCTACACGACGCTCTTCCGATC*T	CAAGCAGAAGACGGCATACGAGATTGCAATCGGTGACTGGAGTTCAGACGTGTGCTCTTCCGATC*T
D5_358	GTTTGGAA			D7_358	GTAAGCGA	TCGCTTAC		AATGATACGGCGACCACCGAGATCTACACGTTTGGAAACACTCTTTCCCTACACGACGCTCTTCCGATC*T	CAAGCAGAAGACGGCATACGAGATTCGCTTACGTGACTGGAGTTCAGACGTGTGCTCTTCCGATC*T
D5_359	ACCACTGG			D7_359	TTCTTCAG	CTGAAGAA		AATGATACGGCGACCACCGAGATCTACACACCACTGGACACTCTTTCCCTACACGACGCTCTTCCGATC*T	CAAGCAGAAGACGGCATACGAGATCTGAAGAAGTGACTGGAGTTCAGACGTGTGCTCTTCCGATC*T
D5_360	TTCCGGTG			D7_360	CGTGGATA	TATCCACG		AATGATACGGCGACCACCGAGATCTACACTTCCGGTGACACTCTTTCCCTACACGACGCTCTTCCGATC*T	CAAGCAGAAGACGGCATACGAGATTATCCACGGTGACTGGAGTTCAGACGTGTGCTCTTCCGATC*T
D5_361	AGTCGATG			D7_361	CACTTTGC	GCAAAGTG		AATGATACGGCGACCACCGAGATCTACACAGTCGATGACACTCTTTCCCTACACGACGCTCTTCCGATC*T	CAAGCAGAAGACGGCATACGAGATGCAAAGTGGTGACTGGAGTTCAGACGTGTGCTCTTCCGATC*T
D5_362	GATGCGTC			D7_362	GTGTCGGT	ACCGACAC		AATGATACGGCGACCACCGAGATCTACACGATGCGTCACACTCTTTCCCTACACGACGCTCTTCCGATC*T	CAAGCAGAAGACGGCATACGAGATACCGACACGTGACTGGAGTTCAGACGTGTGCTCTTCCGATC*T
D5_363	ACATTCTA			D7_363	ACGTAACA	TGTTACGT		AATGATACGGCGACCACCGAGATCTACACACATTCTAACACTCTTTCCCTACACGACGCTCTTCCGATC*T	CAAGCAGAAGACGGCATACGAGATTGTTACGTGTGACTGGAGTTCAGACGTGTGCTCTTCCGATC*T
D5_364	CAGACTGG			D7_364	CTTTCGAC	GTCGAAAG		AATGATACGGCGACCACCGAGATCTACACCAGACTGGACACTCTTTCCCTACACGACGCTCTTCCGATC*T	CAAGCAGAAGACGGCATACGAGATGTCGAAAGGTGACTGGAGTTCAGACGTGTGCTCTTCCGATC*T
D5_365	ATAGCGTT			D7_365	GTGACCTA	TAGGTCAC		AATGATACGGCGACCACCGAGATCTACACATAGCGTTACACTCTTTCCCTACACGACGCTCTTCCGATC*T	CAAGCAGAAGACGGCATACGAGATTAGGTCACGTGACTGGAGTTCAGACGTGTGCTCTTCCGATC*T
D5_366	TACGCCGA			D7_366	GTCCTAAC	GTTAGGAC		AATGATACGGCGACCACCGAGATCTACACTACGCCGAACACTCTTTCCCTACACGACGCTCTTCCGATC*T	CAAGCAGAAGACGGCATACGAGATGTTAGGACGTGACTGGAGTTCAGACGTGTGCTCTTCCGATC*T

D5_367	AGTCTGCC			D7_367	GTGCAGGG	CCCTGCAC		AATGATACGGCGACCACCGAGATCTACACAGTCTGCCACACTCTTTCCCTACACGACGCTCTTCCGATC*T	CAAGCAGAAGACGGCATACGAGATCCCTGCACGTGACTGGAGTTCAGACGTGTGCTCTTCCGATC*T
D5_368	GCAACCTA			D7_368	TGCGGTGT	ACACCGCA		AATGATACGGCGACCACCGAGATCTACACGCAACCTAACACTCTTTCCCTACACGACGCTCTTCCGATC*T	CAAGCAGAAGACGGCATACGAGATACACCGCAGTGACTGGAGTTCAGACGTGTGCTCTTCCGATC*T
D5_369	TTCCAAAT			D7_369	AAATGCCT	AGGCATTT		AATGATACGGCGACCACCGAGATCTACACTTCCAAATACACTCTTTCCCTACACGACGCTCTTCCGATC*T	CAAGCAGAAGACGGCATACGAGATAGGCATTTGTGACTGGAGTTCAGACGTGTGCTCTTCCGATC*T
D5_370	GAGCTAAA			D7_370	TCACAACA	TGTTGTGA		AATGATACGGCGACCACCGAGATCTACACGAGCTAAAACACTCTTTCCCTACACGACGCTCTTCCGATC*T	CAAGCAGAAGACGGCATACGAGATTGTTGTGAGTGACTGGAGTTCAGACGTGTGCTCTTCCGATC*T
D5_371	CGACGCGG			D7_371	AATGCTTG	CAAGCATT		AATGATACGGCGACCACCGAGATCTACACCGACGCGGACACTCTTTCCCTACACGACGCTCTTCCGATC*T	CAAGCAGAAGACGGCATACGAGATCAAGCATTGTGACTGGAGTTCAGACGTGTGCTCTTCCGATC*T
D5_372	TTTCATTT			D7_372	CGATAGGC	GCCTATCG		AATGATACGGCGACCACCGAGATCTACACTTTCATTTACACTCTTTCCCTACACGACGCTCTTCCGATC*T	CAAGCAGAAGACGGCATACGAGATGCCTATCGGTGACTGGAGTTCAGACGTGTGCTCTTCCGATC*T
D5_373	TCAGCGAC			D7_373	GAGCATAG	CTATGCTC		AATGATACGGCGACCACCGAGATCTACACTCAGCGACACACTCTTTCCCTACACGACGCTCTTCCGATC*T	CAAGCAGAAGACGGCATACGAGATCTATGCTCGTGACTGGAGTTCAGACGTGTGCTCTTCCGATC*T
D5_374	GAAGCACA			D7_374	TTCTTTGA	TCAAAGAA		AATGATACGGCGACCACCGAGATCTACACGAAGCACAACACTCTTTCCCTACACGACGCTCTTCCGATC*T	CAAGCAGAAGACGGCATACGAGATTCAAAGAAGTGACTGGAGTTCAGACGTGTGCTCTTCCGATC*T
D5_375	ACAAGCGG			D7_375	GGGCCGTG	CACGGCCC		AATGATACGGCGACCACCGAGATCTACACACAAGCGGACACTCTTTCCCTACACGACGCTCTTCCGATC*T	CAAGCAGAAGACGGCATACGAGATCACGGCCCGTGACTGGAGTTCAGACGTGTGCTCTTCCGATC*T
D5_376	ACCCGATT			D7_376	CACCTCCA	TGGAGGTG		AATGATACGGCGACCACCGAGATCTACACACCCGATTACACTCTTTCCCTACACGACGCTCTTCCGATC*T	CAAGCAGAAGACGGCATACGAGATTGGAGGTGGTGACTGGAGTTCAGACGTGTGCTCTTCCGATC*T
D5_377	CCGCTACG			D7_377	CCGATCAT	ATGATCGG		AATGATACGGCGACCACCGAGATCTACACCCGCTACGACACTCTTTCCCTACACGACGCTCTTCCGATC*T	CAAGCAGAAGACGGCATACGAGATATGATCGGGTGACTGGAGTTCAGACGTGTGCTCTTCCGATC*T
D5_378	TCACTCTT			D7_378	GGCGTCAT	ATGACGCC		AATGATACGGCGACCACCGAGATCTACACTCACTCTTACACTCTTTCCCTACACGACGCTCTTCCGATC*T	CAAGCAGAAGACGGCATACGAGATATGACGCCGTGACTGGAGTTCAGACGTGTGCTCTTCCGATC*T
D5_379	CCAGAACA			D7_379	GTCGGCGG	CCGCCGAC		AATGATACGGCGACCACCGAGATCTACACCCAGAACAACACTCTTTCCCTACACGACGCTCTTCCGATC*T	CAAGCAGAAGACGGCATACGAGATCCGCCGACGTGACTGGAGTTCAGACGTGTGCTCTTCCGATC*T
D5_380	TAGTGTGA			D7_380	CTATACCG	CGGTATAG		AATGATACGGCGACCACCGAGATCTACACTAGTGTGAACACTCTTTCCCTACACGACGCTCTTCCGATC*T	CAAGCAGAAGACGGCATACGAGATCGGTATAGGTGACTGGAGTTCAGACGTGTGCTCTTCCGATC*T
D5_381	ACTTTAGG			D7_381	TTAAACAT	ATGTTTAA		AATGATACGGCGACCACCGAGATCTACACACTTTAGGACACTCTTTCCCTACACGACGCTCTTCCGATC*T	CAAGCAGAAGACGGCATACGAGATATGTTTAAGTGACTGGAGTTCAGACGTGTGCTCTTCCGATC*T
D5_382	ATTCTACG			D7_382	CACTCTAG	CTAGAGTG		AATGATACGGCGACCACCGAGATCTACACATTCTACGACACTCTTTCCCTACACGACGCTCTTCCGATC*T	CAAGCAGAAGACGGCATACGAGATCTAGAGTGGTGACTGGAGTTCAGACGTGTGCTCTTCCGATC*T
D5_383	CCCTGCTG			D7_383	GCAGCAGT	ACTGCTGC		AATGATACGGCGACCACCGAGATCTACACCCCTGCTGACACTCTTTCCCTACACGACGCTCTTCCGATC*T	CAAGCAGAAGACGGCATACGAGATACTGCTGCGTGACTGGAGTTCAGACGTGTGCTCTTCCGATC*T
D5_384	ATACAACC			D7_384	TACCATTC	GAATGGTA		AATGATACGGCGACCACCGAGATCTACACATACAACCACACTCTTTCCCTACACGACGCTCTTCCGATC*T	CAAGCAGAAGACGGCATACGAGATGAATGGTAGTGACTGGAGTTCAGACGTGTGCTCTTCCGATC*T

SetR

i5 barcodes   i7 barcodes

	**Sequence For Oligo**				**Sequence Obtained**	**Sequence for oligo**		i5 oligo to order		i7 oligo to order
D5_001	ATAGAGGC			D7_001	TGGTTAGC	GCTAACCA		AATGATACGGCGACCACCGAGATCTACACATAGAGGCACACTCTTTCCCTACACGACGCTCTTCCGATC*T		CAAGCAGAAGACGGCATACGAGATGCTAACCAGTGACTGGAGTTCAGACGTGTGCTCTTCCGATC*T
D5_002	TTGCTGGT			D7_002	GAACTGGG	CCCAGTTC		AATGATACGGCGACCACCGAGATCTACACTTGCTGGTACACTCTTTCCCTACACGACGCTCTTCCGATC*T		CAAGCAGAAGACGGCATACGAGATCCCAGTTCGTGACTGGAGTTCAGACGTGTGCTCTTCCGATC*T
D5_003	TTATTGCG			D7_003	CGTGACAC	GTGTCACG		AATGATACGGCGACCACCGAGATCTACACTTATTGCGACACTCTTTCCCTACACGACGCTCTTCCGATC*T		CAAGCAGAAGACGGCATACGAGATGTGTCACGGTGACTGGAGTTCAGACGTGTGCTCTTCCGATC*T
D5_004	CAAATGAG			D7_004	CATTTCGG	CCGAAATG		AATGATACGGCGACCACCGAGATCTACACCAAATGAGACACTCTTTCCCTACACGACGCTCTTCCGATC*T		CAAGCAGAAGACGGCATACGAGATCCGAAATGGTGACTGGAGTTCAGACGTGTGCTCTTCCGATC*T
D5_005	AGTACCGC			D7_005	CCATAAGA	TCTTATGG		AATGATACGGCGACCACCGAGATCTACACAGTACCGCACACTCTTTCCCTACACGACGCTCTTCCGATC*T		CAAGCAGAAGACGGCATACGAGATTCTTATGGGTGACTGGAGTTCAGACGTGTGCTCTTCCGATC*T
D5_006	CTCCATAA			D7_006	ACTTAGAG	CTCTAAGT		AATGATACGGCGACCACCGAGATCTACACCTCCATAAACACTCTTTCCCTACACGACGCTCTTCCGATC*T		CAAGCAGAAGACGGCATACGAGATCTCTAAGTGTGACTGGAGTTCAGACGTGTGCTCTTCCGATC*T
D5_007	CCAAACCC			D7_007	TCAGGAAA	TTTCCTGA		AATGATACGGCGACCACCGAGATCTACACCCAAACCCACACTCTTTCCCTACACGACGCTCTTCCGATC*T		CAAGCAGAAGACGGCATACGAGATTTTCCTGAGTGACTGGAGTTCAGACGTGTGCTCTTCCGATC*T
D5_008	ACTTAAAC			D7_008	CTATTGGG	CCCAATAG		AATGATACGGCGACCACCGAGATCTACACACTTAAACACACTCTTTCCCTACACGACGCTCTTCCGATC*T		CAAGCAGAAGACGGCATACGAGATCCCAATAGGTGACTGGAGTTCAGACGTGTGCTCTTCCGATC*T
D5_009	GTGGTGTG			D7_009	AGACAGGT	ACCTGTCT		AATGATACGGCGACCACCGAGATCTACACGTGGTGTGACACTCTTTCCCTACACGACGCTCTTCCGATC*T		CAAGCAGAAGACGGCATACGAGATACCTGTCTGTGACTGGAGTTCAGACGTGTGCTCTTCCGATC*T
D5_010	CGCGCAAA			D7_010	CAGCTGGC	GCCAGCTG		AATGATACGGCGACCACCGAGATCTACACCGCGCAAAACACTCTTTCCCTACACGACGCTCTTCCGATC*T		CAAGCAGAAGACGGCATACGAGATGCCAGCTGGTGACTGGAGTTCAGACGTGTGCTCTTCCGATC*T
D5_011	AAATCAAG			D7_011	AGCGGGTC	GACCCGCT		AATGATACGGCGACCACCGAGATCTACACAAATCAAGACACTCTTTCCCTACACGACGCTCTTCCGATC*T		CAAGCAGAAGACGGCATACGAGATGACCCGCTGTGACTGGAGTTCAGACGTGTGCTCTTCCGATC*T
D5_012	GTCAGTAT			D7_012	GTCGAGTT	AACTCGAC		AATGATACGGCGACCACCGAGATCTACACGTCAGTATACACTCTTTCCCTACACGACGCTCTTCCGATC*T		CAAGCAGAAGACGGCATACGAGATAACTCGACGTGACTGGAGTTCAGACGTGTGCTCTTCCGATC*T
D5_013	CTTGTTAA			D7_013	CGTCACCA	TGGTGACG		AATGATACGGCGACCACCGAGATCTACACCTTGTTAAACACTCTTTCCCTACACGACGCTCTTCCGATC*T		CAAGCAGAAGACGGCATACGAGATTGGTGACGGTGACTGGAGTTCAGACGTGTGCTCTTCCGATC*T
D5_014	AAAGTGCG			D7_014	GTGTTTCT	AGAAACAC		AATGATACGGCGACCACCGAGATCTACACAAAGTGCGACACTCTTTCCCTACACGACGCTCTTCCGATC*T		CAAGCAGAAGACGGCATACGAGATAGAAACACGTGACTGGAGTTCAGACGTGTGCTCTTCCGATC*T
D5_015	AAGTACAG			D7_015	GGCACTCA	TGAGTGCC		AATGATACGGCGACCACCGAGATCTACACAAGTACAGACACTCTTTCCCTACACGACGCTCTTCCGATC*T		CAAGCAGAAGACGGCATACGAGATTGAGTGCCGTGACTGGAGTTCAGACGTGTGCTCTTCCGATC*T
D5_016	GCGCGCTA			D7_016	GTCATCTT	AAGATGAC		AATGATACGGCGACCACCGAGATCTACACGCGCGCTAACACTCTTTCCCTACACGACGCTCTTCCGATC*T		CAAGCAGAAGACGGCATACGAGATAAGATGACGTGACTGGAGTTCAGACGTGTGCTCTTCCGATC*T
D5_017	AAGCAAAC			D7_017	TCGCACTT	AAGTGCGA		AATGATACGGCGACCACCGAGATCTACACAAGCAAACACACTCTTTCCCTACACGACGCTCTTCCGATC*T		CAAGCAGAAGACGGCATACGAGATAAGTGCGAGTGACTGGAGTTCAGACGTGTGCTCTTCCGATC*T
D5_018	AGGCGCTC			D7_018	GCGGTGGG	CCCACCGC		AATGATACGGCGACCACCGAGATCTACACAGGCGCTCACACTCTTTCCCTACACGACGCTCTTCCGATC*T		CAAGCAGAAGACGGCATACGAGATCCCACCGCGTGACTGGAGTTCAGACGTGTGCTCTTCCGATC*T
D5_019	ACATCACC			D7_019	ATAATAAG	CTTATTAT		AATGATACGGCGACCACCGAGATCTACACACATCACCACACTCTTTCCCTACACGACGCTCTTCCGATC*T		CAAGCAGAAGACGGCATACGAGATCTTATTATGTGACTGGAGTTCAGACGTGTGCTCTTCCGATC*T
D5_020	TATAATTA			D7_020	ACAGCGCG	CGCGCTGT		AATGATACGGCGACCACCGAGATCTACACTATAATTAACACTCTTTCCCTACACGACGCTCTTCCGATC*T		CAAGCAGAAGACGGCATACGAGATCGCGCTGTGTGACTGGAGTTCAGACGTGTGCTCTTCCGATC*T
D5_021	TATAAAGC			D7_021	AGAGCTTA	TAAGCTCT		AATGATACGGCGACCACCGAGATCTACACTATAAAGCACACTCTTTCCCTACACGACGCTCTTCCGATC*T		CAAGCAGAAGACGGCATACGAGATTAAGCTCTGTGACTGGAGTTCAGACGTGTGCTCTTCCGATC*T
D5_022	TCCACACC			D7_022	GTTCGAGC	GCTCGAAC		AATGATACGGCGACCACCGAGATCTACACTCCACACCACACTCTTTCCCTACACGACGCTCTTCCGATC*T		CAAGCAGAAGACGGCATACGAGATGCTCGAACGTGACTGGAGTTCAGACGTGTGCTCTTCCGATC*T
D5_023	TATCAAAG			D7_023	CTAAGTCC	GGACTTAG		AATGATACGGCGACCACCGAGATCTACACTATCAAAGACACTCTTTCCCTACACGACGCTCTTCCGATC*T		CAAGCAGAAGACGGCATACGAGATGGACTTAGGTGACTGGAGTTCAGACGTGTGCTCTTCCGATC*T
D5_024	CGTAATCG			D7_024	GCGTCTTC	GAAGACGC		AATGATACGGCGACCACCGAGATCTACACCGTAATCGACACTCTTTCCCTACACGACGCTCTTCCGATC*T		CAAGCAGAAGACGGCATACGAGATGAAGACGCGTGACTGGAGTTCAGACGTGTGCTCTTCCGATC*T
D5_025	CACAGTTC			D7_025	GAGCTTTC	GAAAGCTC		AATGATACGGCGACCACCGAGATCTACACCACAGTTCACACTCTTTCCCTACACGACGCTCTTCCGATC*T		CAAGCAGAAGACGGCATACGAGATGAAAGCTCGTGACTGGAGTTCAGACGTGTGCTCTTCCGATC*T
D5_026	TCGAGGCG			D7_026	GTGCCGCA	TGCGGCAC		AATGATACGGCGACCACCGAGATCTACACTCGAGGCGACACTCTTTCCCTACACGACGCTCTTCCGATC*T		CAAGCAGAAGACGGCATACGAGATTGCGGCACGTGACTGGAGTTCAGACGTGTGCTCTTCCGATC*T
D5_027	TGTTCAGC			D7_027	TGTTTGGG	CCCAAACA		AATGATACGGCGACCACCGAGATCTACACTGTTCAGCACACTCTTTCCCTACACGACGCTCTTCCGATC*T		CAAGCAGAAGACGGCATACGAGATCCCAAACAGTGACTGGAGTTCAGACGTGTGCTCTTCCGATC*T
D5_028	TAAATAGA			D7_028	GGTGCATT	AATGCACC		AATGATACGGCGACCACCGAGATCTACACTAAATAGAACACTCTTTCCCTACACGACGCTCTTCCGATC*T		CAAGCAGAAGACGGCATACGAGATAATGCACCGTGACTGGAGTTCAGACGTGTGCTCTTCCGATC*T
D5_029	GTCCCGCA			D7_029	GATCTTGA	TCAAGATC		AATGATACGGCGACCACCGAGATCTACACGTCCCGCAACACTCTTTCCCTACACGACGCTCTTCCGATC*T		CAAGCAGAAGACGGCATACGAGATTCAAGATCGTGACTGGAGTTCAGACGTGTGCTCTTCCGATC*T
D5_030	AATCGGCG			D7_030	GCATAAAG	CTTTATGC		AATGATACGGCGACCACCGAGATCTACACAATCGGCGACACTCTTTCCCTACACGACGCTCTTCCGATC*T		CAAGCAGAAGACGGCATACGAGATCTTTATGCGTGACTGGAGTTCAGACGTGTGCTCTTCCGATC*T
D5_031	GAATCTCT			D7_031	ACTAACCA	TGGTTAGT		AATGATACGGCGACCACCGAGATCTACACGAATCTCTACACTCTTTCCCTACACGACGCTCTTCCGATC*T		CAAGCAGAAGACGGCATACGAGATTGGTTAGTGTGACTGGAGTTCAGACGTGTGCTCTTCCGATC*T
D5_032	AGGCAGGC			D7_032	CAGCTCAG	CTGAGCTG		AATGATACGGCGACCACCGAGATCTACACAGGCAGGCACACTCTTTCCCTACACGACGCTCTTCCGATC*T		CAAGCAGAAGACGGCATACGAGATCTGAGCTGGTGACTGGAGTTCAGACGTGTGCTCTTCCGATC*T
D5_033	TCCCGCTC			D7_033	GATCGCCT	AGGCGATC		AATGATACGGCGACCACCGAGATCTACACTCCCGCTCACACTCTTTCCCTACACGACGCTCTTCCGATC*T		CAAGCAGAAGACGGCATACGAGATAGGCGATCGTGACTGGAGTTCAGACGTGTGCTCTTCCGATC*T
D5_034	CGCTTCAC			D7_034	GCGACTGA	TCAGTCGC		AATGATACGGCGACCACCGAGATCTACACCGCTTCACACACTCTTTCCCTACACGACGCTCTTCCGATC*T		CAAGCAGAAGACGGCATACGAGATTCAGTCGCGTGACTGGAGTTCAGACGTGTGCTCTTCCGATC*T

D5_035	ATCGCATG			D7_035	TTTATCCA	TGGATAAA		AATGATACGGCGACCACCGAGATCTACACATCGCATGACACTCTTTCCCTACACGACGCTCTTCCGATC*T	CAAGCAGAAGACGGCATACGAGATTGGATAAAGTGACTGGAGTTCAGACGTGTGCTCTTCCGATC*T
D5_036	GTGATTGG			D7_036	GTGCTCGT	ACGAGCAC		AATGATACGGCGACCACCGAGATCTACACGTGATTGGACACTCTTTCCCTACACGACGCTCTTCCGATC*T	CAAGCAGAAGACGGCATACGAGATACGAGCACGTGACTGGAGTTCAGACGTGTGCTCTTCCGATC*T
D5_037	GAGGTCAC			D7_037	CGAGGACC	GGTCCTCG		AATGATACGGCGACCACCGAGATCTACACGAGGTCACACACTCTTTCCCTACACGACGCTCTTCCGATC*T	CAAGCAGAAGACGGCATACGAGATGGTCCTCGGTGACTGGAGTTCAGACGTGTGCTCTTCCGATC*T
D5_038	CACAAGGA			D7_038	TCCGGATT	AATCCGGA		AATGATACGGCGACCACCGAGATCTACACCACAAGGAACACTCTTTCCCTACACGACGCTCTTCCGATC*T	CAAGCAGAAGACGGCATACGAGATAATCCGGAGTGACTGGAGTTCAGACGTGTGCTCTTCCGATC*T
D5_039	GGAAGACA			D7_039	GCTTTGGC	GCCAAAGC		AATGATACGGCGACCACCGAGATCTACACGGAAGACAACACTCTTTCCCTACACGACGCTCTTCCGATC*T	CAAGCAGAAGACGGCATACGAGATGCCAAAGCGTGACTGGAGTTCAGACGTGTGCTCTTCCGATC*T
D5_040	GAAATTAT			D7_040	AACGACTG	CAGTCGTT		AATGATACGGCGACCACCGAGATCTACACGAAATTATACACTCTTTCCCTACACGACGCTCTTCCGATC*T	CAAGCAGAAGACGGCATACGAGATCAGTCGTTGTGACTGGAGTTCAGACGTGTGCTCTTCCGATC*T
D5_041	CTTCTTGG			D7_041	GACCGTCA	TGACGGTC		AATGATACGGCGACCACCGAGATCTACACCTTCTTGGACACTCTTTCCCTACACGACGCTCTTCCGATC*T	CAAGCAGAAGACGGCATACGAGATTGACGGTCGTGACTGGAGTTCAGACGTGTGCTCTTCCGATC*T
D5_042	ACTTCTCG			D7_042	CTCTGGGT	ACCCAGAG		AATGATACGGCGACCACCGAGATCTACACACTTCTCGACACTCTTTCCCTACACGACGCTCTTCCGATC*T	CAAGCAGAAGACGGCATACGAGATACCCAGAGGTGACTGGAGTTCAGACGTGTGCTCTTCCGATC*T
D5_043	AGCTATAG			D7_043	GTTTGTTT	AAACAAAC		AATGATACGGCGACCACCGAGATCTACACAGCTATAGACACTCTTTCCCTACACGACGCTCTTCCGATC*T	CAAGCAGAAGACGGCATACGAGATAAACAAACGTGACTGGAGTTCAGACGTGTGCTCTTCCGATC*T
D5_044	CGAAATTA			D7_044	CGGTCGCT	AGCGACCG		AATGATACGGCGACCACCGAGATCTACACCGAAATTAACACTCTTTCCCTACACGACGCTCTTCCGATC*T	CAAGCAGAAGACGGCATACGAGATAGCGACCGGTGACTGGAGTTCAGACGTGTGCTCTTCCGATC*T
D5_045	CTGTGCTT			D7_045	CCTCGAGG	CCTCGAGG		AATGATACGGCGACCACCGAGATCTACACCTGTGCTTACACTCTTTCCCTACACGACGCTCTTCCGATC*T	CAAGCAGAAGACGGCATACGAGATCCTCGAGGGTGACTGGAGTTCAGACGTGTGCTCTTCCGATC*T
D5_046	TAGAGATA			D7_046	CTCGTGTC	GACACGAG		AATGATACGGCGACCACCGAGATCTACACTAGAGATAACACTCTTTCCCTACACGACGCTCTTCCGATC*T	CAAGCAGAAGACGGCATACGAGATGACACGAGGTGACTGGAGTTCAGACGTGTGCTCTTCCGATC*T
D5_047	AGCGTATT			D7_047	TGGCGGTC	GACCGCCA		AATGATACGGCGACCACCGAGATCTACACAGCGTATTACACTCTTTCCCTACACGACGCTCTTCCGATC*T	CAAGCAGAAGACGGCATACGAGATGACCGCCAGTGACTGGAGTTCAGACGTGTGCTCTTCCGATC*T
D5_048	ATCGCTAA			D7_048	TGCTGGCA	TGCCAGCA		AATGATACGGCGACCACCGAGATCTACACATCGCTAAACACTCTTTCCCTACACGACGCTCTTCCGATC*T	CAAGCAGAAGACGGCATACGAGATTGCCAGCAGTGACTGGAGTTCAGACGTGTGCTCTTCCGATC*T
D5_049	ATGGAACA			D7_049	AGAGTCGA	TCGACTCT		AATGATACGGCGACCACCGAGATCTACACATGGAACAACACTCTTTCCCTACACGACGCTCTTCCGATC*T	CAAGCAGAAGACGGCATACGAGATTCGACTCTGTGACTGGAGTTCAGACGTGTGCTCTTCCGATC*T
D5_050	ACTTCCGA			D7_050	TGATTTCC	GGAAATCA		AATGATACGGCGACCACCGAGATCTACACACTTCCGAACACTCTTTCCCTACACGACGCTCTTCCGATC*T	CAAGCAGAAGACGGCATACGAGATGGAAATCAGTGACTGGAGTTCAGACGTGTGCTCTTCCGATC*T
D5_051	TCGTTTCG			D7_051	CAAGACCA	TGGTCTTG		AATGATACGGCGACCACCGAGATCTACACTCGTTTCGACACTCTTTCCCTACACGACGCTCTTCCGATC*T	CAAGCAGAAGACGGCATACGAGATTGGTCTTGGTGACTGGAGTTCAGACGTGTGCTCTTCCGATC*T
D5_052	TGGCATAA			D7_052	AAGCTCTT	AAGAGCTT		AATGATACGGCGACCACCGAGATCTACACTGGCATAAACACTCTTTCCCTACACGACGCTCTTCCGATC*T	CAAGCAGAAGACGGCATACGAGATAAGAGCTTGTGACTGGAGTTCAGACGTGTGCTCTTCCGATC*T
D5_053	ACGTTTAA			D7_053	TTTAAATG	CATTTAAA		AATGATACGGCGACCACCGAGATCTACACACGTTTAAACACTCTTTCCCTACACGACGCTCTTCCGATC*T	CAAGCAGAAGACGGCATACGAGATCATTTAAAGTGACTGGAGTTCAGACGTGTGCTCTTCCGATC*T
D5_054	ACGCCGCG			D7_054	ACCTGCGG	CCGCAGGT		AATGATACGGCGACCACCGAGATCTACACACGCCGCGACACTCTTTCCCTACACGACGCTCTTCCGATC*T	CAAGCAGAAGACGGCATACGAGATCCGCAGGTGTGACTGGAGTTCAGACGTGTGCTCTTCCGATC*T
D5_055	TAGTATTC			D7_055	TTGATGTG	CACATCAA		AATGATACGGCGACCACCGAGATCTACACTAGTATTCACACTCTTTCCCTACACGACGCTCTTCCGATC*T	CAAGCAGAAGACGGCATACGAGATCACATCAAGTGACTGGAGTTCAGACGTGTGCTCTTCCGATC*T
D5_056	ATCCATGT			D7_056	TCCTCGGT	ACCGAGGA		AATGATACGGCGACCACCGAGATCTACACATCCATGTACACTCTTTCCCTACACGACGCTCTTCCGATC*T	CAAGCAGAAGACGGCATACGAGATACCGAGGAGTGACTGGAGTTCAGACGTGTGCTCTTCCGATC*T
D5_057	GGTCCCTC			D7_057	GACCGCAG	CTGCGGTC		AATGATACGGCGACCACCGAGATCTACACGGTCCCTCACACTCTTTCCCTACACGACGCTCTTCCGATC*T	CAAGCAGAAGACGGCATACGAGATCTGCGGTCGTGACTGGAGTTCAGACGTGTGCTCTTCCGATC*T
D5_058	TAATATAG			D7_058	GGTCTTCC	GGAAGACC		AATGATACGGCGACCACCGAGATCTACACTAATATAGACACTCTTTCCCTACACGACGCTCTTCCGATC*T	CAAGCAGAAGACGGCATACGAGATGGAAGACCGTGACTGGAGTTCAGACGTGTGCTCTTCCGATC*T
D5_059	CAAACAAA			D7_059	GTTTCATG	CATGAAAC		AATGATACGGCGACCACCGAGATCTACACCAAACAAAACACTCTTTCCCTACACGACGCTCTTCCGATC*T	CAAGCAGAAGACGGCATACGAGATCATGAAACGTGACTGGAGTTCAGACGTGTGCTCTTCCGATC*T
D5_060	ACCAAAGC			D7_060	AAACATCG	CGATGTTT		AATGATACGGCGACCACCGAGATCTACACACCAAAGCACACTCTTTCCCTACACGACGCTCTTCCGATC*T	CAAGCAGAAGACGGCATACGAGATCGATGTTTGTGACTGGAGTTCAGACGTGTGCTCTTCCGATC*T
D5_061	AGCGCCCT			D7_061	GGGCGGCT	AGCCGCCC		AATGATACGGCGACCACCGAGATCTACACAGCGCCCTACACTCTTTCCCTACACGACGCTCTTCCGATC*T	CAAGCAGAAGACGGCATACGAGATAGCCGCCCGTGACTGGAGTTCAGACGTGTGCTCTTCCGATC*T
D5_062	CTACCGTG			D7_062	GCGAGGCG	CGCCTCGC		AATGATACGGCGACCACCGAGATCTACACCTACCGTGACACTCTTTCCCTACACGACGCTCTTCCGATC*T	CAAGCAGAAGACGGCATACGAGATCGCCTCGCGTGACTGGAGTTCAGACGTGTGCTCTTCCGATC*T
D5_385	ACGGTAAG			D7_385	TCTTCACG	CGTGAAGA		AATGATACGGCGACCACCGAGATCTACACACGGTAAGACACTCTTTCCCTACACGACGCTCTTCCGATC*T	CAAGCAGAAGACGGCATACGAGATCGTGAAGAGTGACTGGAGTTCAGACGTGTGCTCTTCCGATC*T
D5_064	CTTTGTGC			D7_064	CTTGAGCA	TGCTCAAG		AATGATACGGCGACCACCGAGATCTACACCTTTGTGCACACTCTTTCCCTACACGACGCTCTTCCGATC*T	CAAGCAGAAGACGGCATACGAGATTGCTCAAGGTGACTGGAGTTCAGACGTGTGCTCTTCCGATC*T
D5_065	CCGATAAA			D7_065	GCTGCGCT	AGCGCAGC		AATGATACGGCGACCACCGAGATCTACACCCGATAAAACACTCTTTCCCTACACGACGCTCTTCCGATC*T	CAAGCAGAAGACGGCATACGAGATAGCGCAGCGTGACTGGAGTTCAGACGTGTGCTCTTCCGATC*T
D5_066	GGCTATTC			D7_066	CGGGAACT	AGTTCCCG		AATGATACGGCGACCACCGAGATCTACACGGCTATTCACACTCTTTCCCTACACGACGCTCTTCCGATC*T	CAAGCAGAAGACGGCATACGAGATAGTTCCCGGTGACTGGAGTTCAGACGTGTGCTCTTCCGATC*T
D5_067	CGCCGCCC			D7_067	TGTCAGCT	AGCTGACA		AATGATACGGCGACCACCGAGATCTACACCGCCGCCCACACTCTTTCCCTACACGACGCTCTTCCGATC*T	CAAGCAGAAGACGGCATACGAGATAGCTGACAGTGACTGGAGTTCAGACGTGTGCTCTTCCGATC*T
D5_068	TGCCGGCA			D7_068	CTCTCCGG	CCGGAGAG		AATGATACGGCGACCACCGAGATCTACACTGCCGGCAACACTCTTTCCCTACACGACGCTCTTCCGATC*T	CAAGCAGAAGACGGCATACGAGATCCGGAGAGGTGACTGGAGTTCAGACGTGTGCTCTTCCGATC*T
D5_069	TAAGCTTT			D7_069	CTGCGTTA	TAACGCAG		AATGATACGGCGACCACCGAGATCTACACTAAGCTTTACACTCTTTCCCTACACGACGCTCTTCCGATC*T	CAAGCAGAAGACGGCATACGAGATTAACGCAGGTGACTGGAGTTCAGACGTGTGCTCTTCCGATC*T
D5_070	GCGCCTCT			D7_070	GCCTCATT	AATGAGGC		AATGATACGGCGACCACCGAGATCTACACGCGCCTCTACACTCTTTCCCTACACGACGCTCTTCCGATC*T	CAAGCAGAAGACGGCATACGAGATAATGAGGCGTGACTGGAGTTCAGACGTGTGCTCTTCCGATC*T
D5_071	CCTTTCCT			D7_071	GCGCGGAA	TTCCGCGC		AATGATACGGCGACCACCGAGATCTACACCCTTTCCTACACTCTTTCCCTACACGACGCTCTTCCGATC*T	CAAGCAGAAGACGGCATACGAGATTTCCGCGCGTGACTGGAGTTCAGACGTGTGCTCTTCCGATC*T
D5_072	GATGGCTA			D7_072	AGAGTTAC	GTAACTCT		AATGATACGGCGACCACCGAGATCTACACGATGGCTAACACTCTTTCCCTACACGACGCTCTTCCGATC*T	CAAGCAGAAGACGGCATACGAGATGTAACTCTGTGACTGGAGTTCAGACGTGTGCTCTTCCGATC*T
D5_073	GTGAGATT			D7_073	CCTGTAGC	GCTACAGG		AATGATACGGCGACCACCGAGATCTACACGTGAGATTACACTCTTTCCCTACACGACGCTCTTCCGATC*T	CAAGCAGAAGACGGCATACGAGATGCTACAGGGTGACTGGAGTTCAGACGTGTGCTCTTCCGATC*T
D5_074	AAGCTCCG			D7_074	CTCTTTAT	ATAAAGAG		AATGATACGGCGACCACCGAGATCTACACAAGCTCCGACACTCTTTCCCTACACGACGCTCTTCCGATC*T	CAAGCAGAAGACGGCATACGAGATATAAAGAGGTGACTGGAGTTCAGACGTGTGCTCTTCCGATC*T
D5_075	ATAGGCAA			D7_075	AGAGAGCC	GGCTCTCT		AATGATACGGCGACCACCGAGATCTACACATAGGCAAACACTCTTTCCCTACACGACGCTCTTCCGATC*T	CAAGCAGAAGACGGCATACGAGATGGCTCTCTGTGACTGGAGTTCAGACGTGTGCTCTTCCGATC*T
D5_076	CCGGTGCC			D7_076	CATCCACT	AGTGGATG		AATGATACGGCGACCACCGAGATCTACACCCGGTGCCACACTCTTTCCCTACACGACGCTCTTCCGATC*T	CAAGCAGAAGACGGCATACGAGATAGTGGATGGTGACTGGAGTTCAGACGTGTGCTCTTCCGATC*T
D5_077	TCCCTGCT			D7_077	AATTATCT	AGATAATT		AATGATACGGCGACCACCGAGATCTACACTCCCTGCTACACTCTTTCCCTACACGACGCTCTTCCGATC*T	CAAGCAGAAGACGGCATACGAGATAGATAATTGTGACTGGAGTTCAGACGTGTGCTCTTCCGATC*T
D5_078	TTGTATGT			D7_078	GACAACCG	CGGTTGTC		AATGATACGGCGACCACCGAGATCTACACTTGTATGTACACTCTTTCCCTACACGACGCTCTTCCGATC*T	CAAGCAGAAGACGGCATACGAGATCGGTTGTCGTGACTGGAGTTCAGACGTGTGCTCTTCCGATC*T
D5_079	CGCAGAGG			D7_079	GATACGGA	TCCGTATC		AATGATACGGCGACCACCGAGATCTACACCGCAGAGGACACTCTTTCCCTACACGACGCTCTTCCGATC*T	CAAGCAGAAGACGGCATACGAGATTCCGTATCGTGACTGGAGTTCAGACGTGTGCTCTTCCGATC*T
D5_080	TTTCGCGT			D7_080	CTATGTGA	TCACATAG		AATGATACGGCGACCACCGAGATCTACACTTTCGCGTACACTCTTTCCCTACACGACGCTCTTCCGATC*T	CAAGCAGAAGACGGCATACGAGATTCACATAGGTGACTGGAGTTCAGACGTGTGCTCTTCCGATC*T
D5_081	CGAAGCAA			D7_081	TTGTGCGG	CCGCACAA		AATGATACGGCGACCACCGAGATCTACACCGAAGCAAACACTCTTTCCCTACACGACGCTCTTCCGATC*T	CAAGCAGAAGACGGCATACGAGATCCGCACAAGTGACTGGAGTTCAGACGTGTGCTCTTCCGATC*T
D5_082	GTTAAACC			D7_082	AAAGGTCC	GGACCTTT		AATGATACGGCGACCACCGAGATCTACACGTTAAACCACACTCTTTCCCTACACGACGCTCTTCCGATC*T	CAAGCAGAAGACGGCATACGAGATGGACCTTTGTGACTGGAGTTCAGACGTGTGCTCTTCCGATC*T
D5_083	AGTGTCCG			D7_083	GGTCACAT	ATGTGACC		AATGATACGGCGACCACCGAGATCTACACAGTGTCCGACACTCTTTCCCTACACGACGCTCTTCCGATC*T	CAAGCAGAAGACGGCATACGAGATATGTGACCGTGACTGGAGTTCAGACGTGTGCTCTTCCGATC*T
D5_084	TAACCGCG			D7_084	CATTCTTC	GAAGAATG		AATGATACGGCGACCACCGAGATCTACACTAACCGCGACACTCTTTCCCTACACGACGCTCTTCCGATC*T	CAAGCAGAAGACGGCATACGAGATGAAGAATGGTGACTGGAGTTCAGACGTGTGCTCTTCCGATC*T
D5_085	GAAGAGCC			D7_085	CTCGACGT	ACGTCGAG		AATGATACGGCGACCACCGAGATCTACACGAAGAGCCACACTCTTTCCCTACACGACGCTCTTCCGATC*T	CAAGCAGAAGACGGCATACGAGATACGTCGAGGTGACTGGAGTTCAGACGTGTGCTCTTCCGATC*T
D5_086	ATCCTGAG			D7_086	AGTTGCAA	TTGCAACT		AATGATACGGCGACCACCGAGATCTACACATCCTGAGACACTCTTTCCCTACACGACGCTCTTCCGATC*T	CAAGCAGAAGACGGCATACGAGATTTGCAACTGTGACTGGAGTTCAGACGTGTGCTCTTCCGATC*T
D5_087	CCCGCATC			D7_087	GCGTATAT	ATATACGC		AATGATACGGCGACCACCGAGATCTACACCCCGCATCACACTCTTTCCCTACACGACGCTCTTCCGATC*T	CAAGCAGAAGACGGCATACGAGATATATACGCGTGACTGGAGTTCAGACGTGTGCTCTTCCGATC*T
D5_088	TTACGGAC			D7_088	CACCGCTT	AAGCGGTG		AATGATACGGCGACCACCGAGATCTACACTTACGGACACACTCTTTCCCTACACGACGCTCTTCCGATC*T	CAAGCAGAAGACGGCATACGAGATAAGCGGTGGTGACTGGAGTTCAGACGTGTGCTCTTCCGATC*T
D5_089	AAAGGCTG			D7_089	GTAAGATG	CATCTTAC		AATGATACGGCGACCACCGAGATCTACACAAAGGCTGACACTCTTTCCCTACACGACGCTCTTCCGATC*T	CAAGCAGAAGACGGCATACGAGATCATCTTACGTGACTGGAGTTCAGACGTGTGCTCTTCCGATC*T
D5_090	CTAAAGAA			D7_090	GGGTTTGA	TCAAACCC		AATGATACGGCGACCACCGAGATCTACACCTAAAGAAACACTCTTTCCCTACACGACGCTCTTCCGATC*T	CAAGCAGAAGACGGCATACGAGATTCAAACCCGTGACTGGAGTTCAGACGTGTGCTCTTCCGATC*T
D5_091	AGTTTAAA			D7_091	CGACGTTC	GAACGTCG		AATGATACGGCGACCACCGAGATCTACACAGTTTAAAACACTCTTTCCCTACACGACGCTCTTCCGATC*T	CAAGCAGAAGACGGCATACGAGATGAACGTCGGTGACTGGAGTTCAGACGTGTGCTCTTCCGATC*T
D5_092	TCAAGTTG			D7_092	AATGGGAC	GTCCCATT		AATGATACGGCGACCACCGAGATCTACACTCAAGTTGACACTCTTTCCCTACACGACGCTCTTCCGATC*T	CAAGCAGAAGACGGCATACGAGATGTCCCATTGTGACTGGAGTTCAGACGTGTGCTCTTCCGATC*T
D5_093	GAGGCAAG			D7_093	GTGGGCTT	AAGCCCAC		AATGATACGGCGACCACCGAGATCTACACGAGGCAAGACACTCTTTCCCTACACGACGCTCTTCCGATC*T	CAAGCAGAAGACGGCATACGAGATAAGCCCACGTGACTGGAGTTCAGACGTGTGCTCTTCCGATC*T
D5_094	ACGCACCC			D7_094	TTTCGGAG	CTCCGAAA		AATGATACGGCGACCACCGAGATCTACACACGCACCCACACTCTTTCCCTACACGACGCTCTTCCGATC*T	CAAGCAGAAGACGGCATACGAGATCTCCGAAAGTGACTGGAGTTCAGACGTGTGCTCTTCCGATC*T
D5_386	TACTGTTT			D7_386	TCGCTGAC	GTCAGCGA		AATGATACGGCGACCACCGAGATCTACACTACTGTTTACACTCTTTCCCTACACGACGCTCTTCCGATC*T	CAAGCAGAAGACGGCATACGAGATGTCAGCGAGTGACTGGAGTTCAGACGTGTGCTCTTCCGATC*T
D5_096	CGATGCTC			D7_096	TGCCAATG	CATTGGCA		AATGATACGGCGACCACCGAGATCTACACCGATGCTCACACTCTTTCCCTACACGACGCTCTTCCGATC*T	CAAGCAGAAGACGGCATACGAGATCATTGGCAGTGACTGGAGTTCAGACGTGTGCTCTTCCGATC*T

SetI

i5 barcodes				i7 barcodes						
**i5 Index**	**Sequence in oligo**			**i7 index**	**Sequence obtained**	**Sequence for oligo**		i5 oligo to order		i7 oligo to order
D5_481	AATAACGT			D7_481	AATCGTTA	TAACGATT		AATGATACGGCGACCACCGAGATCTACACAATAACGTACACTCTTTCCCTACACGACGCTCTTCCGATC*T		CAAGCAGAAGACGGCATACGAGATTAACGATTGTGACTGGAGTTCAGACGTGTGCTCTTCCGATC*T
D5_482	TTCTTGAA			D7_482	GTCTACAT	ATGTAGAC		AATGATACGGCGACCACCGAGATCTACACTTCTTGAAACACTCTTTCCCTACACGACGCTCTTCCGATC*T		CAAGCAGAAGACGGCATACGAGATATGTAGACGTGACTGGAGTTCAGACGTGTGCTCTTCCGATC*T
D5_483	GGCAGATC			D7_483	CGCTGCTC	GAGCAGCG		AATGATACGGCGACCACCGAGATCTACACGGCAGATCACACTCTTTCCCTACACGACGCTCTTCCGATC*T		CAAGCAGAAGACGGCATACGAGATGAGCAGCGGTGACTGGAGTTCAGACGTGTGCTCTTCCGATC*T
D5_484	CTATGTTA			D7_484	GATCAACA	TGTTGATC		AATGATACGGCGACCACCGAGATCTACACCTATGTTAACACTCTTTCCCTACACGACGCTCTTCCGATC*T		CAAGCAGAAGACGGCATACGAGATTGTTGATCGTGACTGGAGTTCAGACGTGTGCTCTTCCGATC*T
D5_485	GTTGACGC			D7_485	CGAAGGAC	GTCCTTCG		AATGATACGGCGACCACCGAGATCTACACGTTGACGCACACTCTTTCCCTACACGACGCTCTTCCGATC*T		CAAGCAGAAGACGGCATACGAGATGTCCTTCGGTGACTGGAGTTCAGACGTGTGCTCTTCCGATC*T
D5_486	ATCTACGA			D7_486	GATGCCGG	CCGGCATC		AATGATACGGCGACCACCGAGATCTACACATCTACGAACACTCTTTCCCTACACGACGCTCTTCCGATC*T		CAAGCAGAAGACGGCATACGAGATCCGGCATCGTGACTGGAGTTCAGACGTGTGCTCTTCCGATC*T
D5_487	CTCGACAG			D7_487	CTACGAAG	CTTCGTAG		AATGATACGGCGACCACCGAGATCTACACCTCGACAGACACTCTTTCCCTACACGACGCTCTTCCGATC*T		CAAGCAGAAGACGGCATACGAGATCTTCGTAGGTGACTGGAGTTCAGACGTGTGCTCTTCCGATC*T
D5_488	GAGGCTGC			D7_488	GATGCGTC	GACGCATC		AATGATACGGCGACCACCGAGATCTACACGAGGCTGCACACTCTTTCCCTACACGACGCTCTTCCGATC*T		CAAGCAGAAGACGGCATACGAGATGACGCATCGTGACTGGAGTTCAGACGTGTGCTCTTCCGATC*T
D5_489	CCTCGTAG			D7_489	CTACGGCA	TGCCGTAG		AATGATACGGCGACCACCGAGATCTACACCCTCGTAGACACTCTTTCCCTACACGACGCTCTTCCGATC*T		CAAGCAGAAGACGGCATACGAGATTGCCGTAGGTGACTGGAGTTCAGACGTGTGCTCTTCCGATC*T
D5_490	CATAGGCA			D7_490	GATTCCTT	AAGGAATC		AATGATACGGCGACCACCGAGATCTACACCATAGGCAACACTCTTTCCCTACACGACGCTCTTCCGATC*T		CAAGCAGAAGACGGCATACGAGATAAGGAATCGTGACTGGAGTTCAGACGTGTGCTCTTCCGATC*T
D5_491	AGATGAAC			D7_491	CTACTCGA	TCGAGTAG		AATGATACGGCGACCACCGAGATCTACACAGATGAACACACTCTTTCCCTACACGACGCTCTTCCGATC*T		CAAGCAGAAGACGGCATACGAGATTCGAGTAGGTGACTGGAGTTCAGACGTGTGCTCTTCCGATC*T
D5_492	CCGAGTAT			D7_492	GATTCGAG	CTCGAATC		AATGATACGGCGACCACCGAGATCTACACCCGAGTATACACTCTTTCCCTACACGACGCTCTTCCGATC*T		CAAGCAGAAGACGGCATACGAGATCTCGAATCGTGACTGGAGTTCAGACGTGTGCTCTTCCGATC*T
D5_493	AATATTGA			D7_493	AATCGGCG	CGCCGATT		AATGATACGGCGACCACCGAGATCTACACAATATTGAACACTCTTTCCCTACACGACGCTCTTCCGATC*T		CAAGCAGAAGACGGCATACGAGATCGCCGATTGTGACTGGAGTTCAGACGTGTGCTCTTCCGATC*T
D5_494	GTATACCG			D7_494	TTCGCCGA	TCGGCGAA		AATGATACGGCGACCACCGAGATCTACACGTATACCGACACTCTTTCCCTACACGACGCTCTTCCGATC*T		CAAGCAGAAGACGGCATACGAGATTCGGCGAAGTGACTGGAGTTCAGACGTGTGCTCTTCCGATC*T
D5_495	GATCCAAC			D7_495	CTGGCCTC	GAGGCCAG		AATGATACGGCGACCACCGAGATCTACACGATCCAACACACTCTTTCCCTACACGACGCTCTTCCGATC*T		CAAGCAGAAGACGGCATACGAGATGAGGCCAGGTGACTGGAGTTCAGACGTGTGCTCTTCCGATC*T
D5_496	AGATACGC			D7_496	GAACTTAT	ATAAGTTC		AATGATACGGCGACCACCGAGATCTACACAGATACGCACACTCTTTCCCTACACGACGCTCTTCCGATC*T		CAAGCAGAAGACGGCATACGAGATATAAGTTCGTGACTGGAGTTCAGACGTGTGCTCTTCCGATC*T
D5_497	GGTATCTT			D7_497	CGTATTGG	CCAATACG		AATGATACGGCGACCACCGAGATCTACACGGTATCTTACACTCTTTCCCTACACGACGCTCTTCCGATC*T		CAAGCAGAAGACGGCATACGAGATCCAATACGGTGACTGGAGTTCAGACGTGTGCTCTTCCGATC*T
D5_498	CCTCTGGC			D7_498	GAAGCACA	TGTGCTTC		AATGATACGGCGACCACCGAGATCTACACCCTCTGGCACACTCTTTCCCTACACGACGCTCTTCCGATC*T		CAAGCAGAAGACGGCATACGAGATTGTGCTTCGTGACTGGAGTTCAGACGTGTGCTCTTCCGATC*T
D5_499	CCATTGTG			D7_499	CTTAATAC	GTATTAAG		AATGATACGGCGACCACCGAGATCTACACCCATTGTGACACTCTTTCCCTACACGACGCTCTTCCGATC*T		CAAGCAGAAGACGGCATACGAGATGTATTAAGGTGACTGGAGTTCAGACGTGTGCTCTTCCGATC*T
D5_500	ACTACGGT			D7_500	GAAGTCTT	AAGACTTC		AATGATACGGCGACCACCGAGATCTACACACTACGGTACACTCTTTCCCTACACGACGCTCTTCCGATC*T		CAAGCAGAAGACGGCATACGAGATAAGACTTCGTGACTGGAGTTCAGACGTGTGCTCTTCCGATC*T
D5_501	AAGTGCTA			D7_501	GAAGAGGC	GCCTCTTC		AATGATACGGCGACCACCGAGATCTACACAAGTGCTAACACTCTTTCCCTACACGACGCTCTTCCGATC*T		CAAGCAGAAGACGGCATACGAGATGCCTCTTCGTGACTGGAGTTCAGACGTGTGCTCTTCCGATC*T
D5_502	GCCGAACG			D7_502	CGGATAAC	GTTATCCG		AATGATACGGCGACCACCGAGATCTACACGCCGAACGACACTCTTTCCCTACACGACGCTCTTCCGATC*T		CAAGCAGAAGACGGCATACGAGATGTTATCCGGTGACTGGAGTTCAGACGTGTGCTCTTCCGATC*T
D5_503	TGTCCACG			D7_503	GAATCTGG	CCAGATTC		AATGATACGGCGACCACCGAGATCTACACTGTCCACGACACTCTTTCCCTACACGACGCTCTTCCGATC*T		CAAGCAGAAGACGGCATACGAGATCCAGATTCGTGACTGGAGTTCAGACGTGTGCTCTTCCGATC*T
D5_504	GACACACT			D7_504	CTGATTGA	TCAATCAG		AATGATACGGCGACCACCGAGATCTACACGACACACTACACTCTTTCCCTACACGACGCTCTTCCGATC*T		CAAGCAGAAGACGGCATACGAGATTCAATCAGGTGACTGGAGTTCAGACGTGTGCTCTTCCGATC*T
D5_505	AATATGCT			D7_505	AATCCGTT	AACGGATT		AATGATACGGCGACCACCGAGATCTACACAATATGCTACACTCTTTCCCTACACGACGCTCTTCCGATC*T		CAAGCAGAAGACGGCATACGAGATAACGGATTGTGACTGGAGTTCAGACGTGTGCTCTTCCGATC*T
D5_506	TTCTCATA			D7_506	TGCGTACA	TGTACGCA		AATGATACGGCGACCACCGAGATCTACACTTCTCATAACACTCTTTCCCTACACGACGCTCTTCCGATC*T		CAAGCAGAAGACGGCATACGAGATTGTACGCAGTGACTGGAGTTCAGACGTGTGCTCTTCCGATC*T
D5_507	TCTGTGAT			D7_507	GAATCAAT	ATTGATTC		AATGATACGGCGACCACCGAGATCTACACTCTGTGATACACTCTTTCCCTACACGACGCTCTTCCGATC*T		CAAGCAGAAGACGGCATACGAGATATTGATTCGTGACTGGAGTTCAGACGTGTGCTCTTCCGATC*T
D5_508	CCGAACTT			D7_508	TGAGTCAG	CTGACTCA		AATGATACGGCGACCACCGAGATCTACACCCGAACTTACACTCTTTCCCTACACGACGCTCTTCCGATC*T		CAAGCAGAAGACGGCATACGAGATCTGACTCAGTGACTGGAGTTCAGACGTGTGCTCTTCCGATC*T
D5_509	GTCTAACA			D7_509	GAATGCTC	GAGCATTC		AATGATACGGCGACCACCGAGATCTACACGTCTAACAACACTCTTTCCCTACACGACGCTCTTCCGATC*T		CAAGCAGAAGACGGCATACGAGATGAGCATTCGTGACTGGAGTTCAGACGTGTGCTCTTCCGATC*T
D5_510	GACGCCAT			D7_510	GAATATCC	GGATATTC		AATGATACGGCGACCACCGAGATCTACACGACGCCATACACTCTTTCCCTACACGACGCTCTTCCGATC*T		CAAGCAGAAGACGGCATACGAGATGGATATTCGTGACTGGAGTTCAGACGTGTGCTCTTCCGATC*T
D5_511	GCCAATGT			D7_511	CTTATGAA	TTCATAAG		AATGATACGGCGACCACCGAGATCTACACGCCAATGTACACTCTTTCCCTACACGACGCTCTTCCGATC*T		CAAGCAGAAGACGGCATACGAGATTTCATAAGGTGACTGGAGTTCAGACGTGTGCTCTTCCGATC*T
D5_512	CCAACGTC			D7_512	TCGGCACC	GGTGCCGA		AATGATACGGCGACCACCGAGATCTACACCCAACGTCACACTCTTTCCCTACACGACGCTCTTCCGATC*T		CAAGCAGAAGACGGCATACGAGATGGTGCCGAGTGACTGGAGTTCAGACGTGTGCTCTTCCGATC*T
D5_513	GTAGATAA			D7_513	AAGAAGCG	CGCTTCTT		AATGATACGGCGACCACCGAGATCTACACGTAGATAAACACTCTTTCCCTACACGACGCTCTTCCGATC*T		CAAGCAGAAGACGGCATACGAGATCGCTTCTTGTGACTGGAGTTCAGACGTGTGCTCTTCCGATC*T
D5_514	CTTACGGC			D7_514	CTCACGAT	ATCGTGAG		AATGATACGGCGACCACCGAGATCTACACCTTACGGCACACTCTTTCCCTACACGACGCTCTTCCGATC*T		CAAGCAGAAGACGGCATACGAGATATCGTGAGGTGACTGGAGTTCAGACGTGTGCTCTTCCGATC*T
D5_515	CCAAGTGC			D7_515	TCGGTCGA	TCGACCGA		AATGATACGGCGACCACCGAGATCTACACCCAAGTGCACACTCTTTCCCTACACGACGCTCTTCCGATC*T		CAAGCAGAAGACGGCATACGAGATTCGACCGAGTGACTGGAGTTCAGACGTGTGCTCTTCCGATC*T
D5_516	CTAACTCA			D7_516	TCGGTAAG	CTTACCGA		AATGATACGGCGACCACCGAGATCTACACCTAACTCAACACTCTTTCCCTACACGACGCTCTTCCGATC*T		CAAGCAGAAGACGGCATACGAGATCTTACCGAGTGACTGGAGTTCAGACGTGTGCTCTTCCGATC*T
D5_517	AATATCTG			D7_517	AAGATACA	TGTATCTT		AATGATACGGCGACCACCGAGATCTACACAATATCTGACACTCTTTCCCTACACGACGCTCTTCCGATC*T		CAAGCAGAAGACGGCATACGAGATTGTATCTTGTGACTGGAGTTCAGACGTGTGCTCTTCCGATC*T
D5_518	TTATATCA			D7_518	GTCGCTGT	ACAGCGAC		AATGATACGGCGACCACCGAGATCTACACTTATATCAACACTCTTTCCCTACACGACGCTCTTCCGATC*T		CAAGCAGAAGACGGCATACGAGATACAGCGACGTGACTGGAGTTCAGACGTGTGCTCTTCCGATC*T
D5_519	CTGCGGAT			D7_519	TCGGATGT	ACATCCGA		AATGATACGGCGACCACCGAGATCTACACCTGCGGATACACTCTTTCCCTACACGACGCTCTTCCGATC*T		CAAGCAGAAGACGGCATACGAGATACATCCGAGTGACTGGAGTTCAGACGTGTGCTCTTCCGATC*T
D5_520	GCGGCTTG			D7_520	CGAGCCGG	CCGGCTCG		AATGATACGGCGACCACCGAGATCTACACGCGGCTTGACACTCTTTCCCTACACGACGCTCTTCCGATC*T		CAAGCAGAAGACGGCATACGAGATCCGGCTCGGTGACTGGAGTTCAGACGTGTGCTCTTCCGATC*T
D5_521	GAGTTGAT			D7_521	CGATTATC	GATAATCG		AATGATACGGCGACCACCGAGATCTACACGAGTTGATACACTCTTTCCCTACACGACGCTCTTCCGATC*T		CAAGCAGAAGACGGCATACGAGATGATAATCGGTGACTGGAGTTCAGACGTGTGCTCTTCCGATC*T
D5_522	GCACTGAG			D7_522	TCGAAGCT	AGCTTCGA		AATGATACGGCGACCACCGAGATCTACACGCACTGAGACACTCTTTCCCTACACGACGCTCTTCCGATC*T		CAAGCAGAAGACGGCATACGAGATAGCTTCGAGTGACTGGAGTTCAGACGTGTGCTCTTCCGATC*T
D5_523	GACCACCT			D7_523	CTATCATT	AATGATAG		AATGATACGGCGACCACCGAGATCTACACGACCACCTACACTCTTTCCCTACACGACGCTCTTCCGATC*T		CAAGCAGAAGACGGCATACGAGATAATGATAGGTGACTGGAGTTCAGACGTGTGCTCTTCCGATC*T
D5_524	TGGCTAGG			D7_524	CGCGCCAA	TTGGCGCG		AATGATACGGCGACCACCGAGATCTACACTGGCTAGGACACTCTTTCCCTACACGACGCTCTTCCGATC*T		CAAGCAGAAGACGGCATACGAGATTTGGCGCGGTGACTGGAGTTCAGACGTGTGCTCTTCCGATC*T
D5_525	CCTACCGG			D7_525	CGAACGGA	TCCGTTCG		AATGATACGGCGACCACCGAGATCTACACCCTACCGGACACTCTTTCCCTACACGACGCTCTTCCGATC*T		CAAGCAGAAGACGGCATACGAGATTCCGTTCGGTGACTGGAGTTCAGACGTGTGCTCTTCCGATC*T
D5_526	GGAGGATG			D7_526	CTACTGAC	GTCAGTAG		AATGATACGGCGACCACCGAGATCTACACGGAGGATGACACTCTTTCCCTACACGACGCTCTTCCGATC*T		CAAGCAGAAGACGGCATACGAGATGTCAGTAGGTGACTGGAGTTCAGACGTGTGCTCTTCCGATC*T
D5_527	CGCTGAAT			D7_527	TCTTAAGT	ACTTAAGA		AATGATACGGCGACCACCGAGATCTACACCGCTGAATACACTCTTTCCCTACACGACGCTCTTCCGATC*T		CAAGCAGAAGACGGCATACGAGATACTTAAGAGTGACTGGAGTTCAGACGTGTGCTCTTCCGATC*T
D5_528	TGTGACGA			D7_528	TTAGAGTC	GACTCTAA		AATGATACGGCGACCACCGAGATCTACACTGTGACGAACACTCTTTCCCTACACGACGCTCTTCCGATC*T		CAAGCAGAAGACGGCATACGAGATGACTCTAAGTGACTGGAGTTCAGACGTGTGCTCTTCCGATC*T
D5_529	AATAGATT			D7_529	AAGACGAA	TTCGTCTT		AATGATACGGCGACCACCGAGATCTACACAATAGATTACACTCTTTCCCTACACGACGCTCTTCCGATC*T		CAAGCAGAAGACGGCATACGAGATTTCGTCTTGTGACTGGAGTTCAGACGTGTGCTCTTCCGATC*T
D5_530	TTAGCGCA			D7_530	TTATTATG	CATAATAA		AATGATACGGCGACCACCGAGATCTACACTTAGCGCAACACTCTTTCCCTACACGACGCTCTTCCGATC*T		CAAGCAGAAGACGGCATACGAGATCATAATAAGTGACTGGAGTTCAGACGTGTGCTCTTCCGATC*T
D5_531	GCGGCCGT			D7_531	CGCTATTA	TAATAGCG		AATGATACGGCGACCACCGAGATCTACACGCGGCCGTACACTCTTTCCCTACACGACGCTCTTCCGATC*T		CAAGCAGAAGACGGCATACGAGATTAATAGCGGTGACTGGAGTTCAGACGTGTGCTCTTCCGATC*T
D5_532	CAGTAACC			D7_532	TCTATCAG	CTGATAGA		AATGATACGGCGACCACCGAGATCTACACCAGTAACCACACTCTTTCCCTACACGACGCTCTTCCGATC*T		CAAGCAGAAGACGGCATACGAGATCTGATAGAGTGACTGGAGTTCAGACGTGTGCTCTTCCGATC*T
D5_533	GCCTAGTA			D7_533	CGGTGGTA	TACCACCG		AATGATACGGCGACCACCGAGATCTACACGCCTAGTAACACTCTTTCCCTACACGACGCTCTTCCGATC*T		CAAGCAGAAGACGGCATACGAGATTACCACCGGTGACTGGAGTTCAGACGTGTGCTCTTCCGATC*T
D5_534	CACGGCGC			D7_534	TCACCAAT	ATTGGTGA		AATGATACGGCGACCACCGAGATCTACACCACGGCGCACACTCTTTCCCTACACGACGCTCTTCCGATC*T		CAAGCAGAAGACGGCATACGAGATATTGGTGAGTGACTGGAGTTCAGACGTGTGCTCTTCCGATC*T
D5_535	GGTGCAGA			D7_535	CTGGAAGC	GCTTCCAG		AATGATACGGCGACCACCGAGATCTACACGGTGCAGAACACTCTTTCCCTACACGACGCTCTTCCGATC*T		CAAGCAGAAGACGGCATACGAGATGCTTCCAGGTGACTGGAGTTCAGACGTGTGCTCTTCCGATC*T

D5_536	TCGCTGAC			D7_536	CGTAAGAG	CTCTTACG		AATGATACGGCGACCACCGAGATCTACACTCGCTGACACACTCTTTCCCTACACGACGCTCTTCCGATC*T	CAAGCAGAAGACGGCATACGAGATCTCTTACGGTGACTGGAGTTCAGACGTGTGCTCTTCCGATC*T
D5_537	CAGCCAGT			D7_537	AAGAGAGC	GCTCTCTT		AATGATACGGCGACCACCGAGATCTACACCAGCCAGTACACTCTTTCCCTACACGACGCTCTTCCGATC*T	CAAGCAGAAGACGGCATACGAGATGCTCTCTTGTGACTGGAGTTCAGACGTGTGCTCTTCCGATC*T
D5_538	CGTCAACC			D7_538	TCAACGAG	CTCGTTGA		AATGATACGGCGACCACCGAGATCTACACCGTCAACCACACTCTTTCCCTACACGACGCTCTTCCGATC*T	CAAGCAGAAGACGGCATACGAGATCTCGTTGAGTGACTGGAGTTCAGACGTGTGCTCTTCCGATC*T
D5_539	GCCGGCGA			D7_539	TGCGAGAC	GTCTCGCA		AATGATACGGCGACCACCGAGATCTACACGCCGGCGAACACTCTTTCCCTACACGACGCTCTTCCGATC*T	CAAGCAGAAGACGGCATACGAGATGTCTCGCAGTGACTGGAGTTCAGACGTGTGCTCTTCCGATC*T
D5_540	GCCTCCGG			D7_540	CCTGGTGT	ACACCAGG		AATGATACGGCGACCACCGAGATCTACACGCCTCCGGACACTCTTTCCCTACACGACGCTCTTCCGATC*T	CAAGCAGAAGACGGCATACGAGATACACCAGGGTGACTGGAGTTCAGACGTGTGCTCTTCCGATC*T
D5_541	AATAGTCC			D7_541	AAGTAAGT	ACTTACTT		AATGATACGGCGACCACCGAGATCTACACAATAGTCCACACTCTTTCCCTACACGACGCTCTTCCGATC*T	CAAGCAGAAGACGGCATACGAGATACTTACTTGTGACTGGAGTTCAGACGTGTGCTCTTCCGATC*T
D5_542	TTAGACGT			D7_542	TGACTGAA	TTCAGTCA		AATGATACGGCGACCACCGAGATCTACACTTAGACGTACACTCTTTCCCTACACGACGCTCTTCCGATC*T	CAAGCAGAAGACGGCATACGAGATTTCAGTCAGTGACTGGAGTTCAGACGTGTGCTCTTCCGATC*T
D5_543	GTGGACTA			D7_543	AAGACTGT	ACAGTCTT		AATGATACGGCGACCACCGAGATCTACACGTGGACTAACACTCTTTCCCTACACGACGCTCTTCCGATC*T	CAAGCAGAAGACGGCATACGAGATACAGTCTTGTGACTGGAGTTCAGACGTGTGCTCTTCCGATC*T
D5_544	CACGGACG			D7_544	CAATGATG	CATCATTG		AATGATACGGCGACCACCGAGATCTACACCACGGACGACACTCTTTCCCTACACGACGCTCTTCCGATC*T	CAAGCAGAAGACGGCATACGAGATCATCATTGGTGACTGGAGTTCAGACGTGTGCTCTTCCGATC*T
D5_545	CACTAGAG			D7_545	CACAGTAA	TTACTGTG		AATGATACGGCGACCACCGAGATCTACACCACTAGAGACACTCTTTCCCTACACGACGCTCTTCCGATC*T	CAAGCAGAAGACGGCATACGAGATTTACTGTGGTGACTGGAGTTCAGACGTGTGCTCTTCCGATC*T
D5_546	GCAGATGG			D7_546	TGGTCATT	AATGACCA		AATGATACGGCGACCACCGAGATCTACACGCAGATGGACACTCTTTCCCTACACGACGCTCTTCCGATC*T	CAAGCAGAAGACGGCATACGAGATAATGACCAGTGACTGGAGTTCAGACGTGTGCTCTTCCGATC*T
D5_547	CTCTCACG			D7_547	CAACCGTG	CACGGTTG		AATGATACGGCGACCACCGAGATCTACACCTCTCACGACACTCTTTCCCTACACGACGCTCTTCCGATC*T	CAAGCAGAAGACGGCATACGAGATCACGGTTGGTGACTGGAGTTCAGACGTGTGCTCTTCCGATC*T
D5_548	GGAATCAC			D7_548	TGGTGCAC	GTGCACCA		AATGATACGGCGACCACCGAGATCTACACGGAATCACACACTCTTTCCCTACACGACGCTCTTCCGATC*T	CAAGCAGAAGACGGCATACGAGATGTGCACCAGTGACTGGAGTTCAGACGTGTGCTCTTCCGATC*T
D5_549	CGTTGACG			D7_549	CCACAATG	CATTGTGG		AATGATACGGCGACCACCGAGATCTACACCGTTGACGACACTCTTTCCCTACACGACGCTCTTCCGATC*T	CAAGCAGAAGACGGCATACGAGATCATTGTGGGTGACTGGAGTTCAGACGTGTGCTCTTCCGATC*T
D5_550	CATCAGGT			D7_550	TGTGTGCC	GGCACACA		AATGATACGGCGACCACCGAGATCTACACCATCAGGTACACTCTTTCCCTACACGACGCTCTTCCGATC*T	CAAGCAGAAGACGGCATACGAGATGGCACACAGTGACTGGAGTTCAGACGTGTGCTCTTCCGATC*T
D5_551	CGTTGTAA			D7_551	CACCACGG	CCGTGGTG		AATGATACGGCGACCACCGAGATCTACACCGTTGTAAACACTCTTTCCCTACACGACGCTCTTCCGATC*T	CAAGCAGAAGACGGCATACGAGATCCGTGGTGGTGACTGGAGTTCAGACGTGTGCTCTTCCGATC*T
D5_552	GGCACGGT			D7_552	TGTGTTAA	TTAACACA		AATGATACGGCGACCACCGAGATCTACACGGCACGGTACACTCTTTCCCTACACGACGCTCTTCCGATC*T	CAAGCAGAAGACGGCATACGAGATTTAACACAGTGACTGGAGTTCAGACGTGTGCTCTTCCGATC*T
D5_553	AATAGCAA			D7_553	AAGTTATC	GATAACTT		AATGATACGGCGACCACCGAGATCTACACAATAGCAAACACTCTTTCCCTACACGACGCTCTTCCGATC*T	CAAGCAGAAGACGGCATACGAGATGATAACTTGTGACTGGAGTTCAGACGTGTGCTCTTCCGATC*T
D5_554	TGATCGGT			D7_554	GTACAGCT	AGCTGTAC		AATGATACGGCGACCACCGAGATCTACACTGATCGGTACACTCTTTCCCTACACGACGCTCTTCCGATC*T	CAAGCAGAAGACGGCATACGAGATAGCTGTACGTGACTGGAGTTCAGACGTGTGCTCTTCCGATC*T
D5_555	AGTAGTAT			D7_555	CAACTGCT	AGCAGTTG		AATGATACGGCGACCACCGAGATCTACACAGTAGTATACACTCTTTCCCTACACGACGCTCTTCCGATC*T	CAAGCAGAAGACGGCATACGAGATAGCAGTTGGTGACTGGAGTTCAGACGTGTGCTCTTCCGATC*T
D5_556	GTTAGAGG			D7_556	CATGATGA	TCATCATG		AATGATACGGCGACCACCGAGATCTACACGTTAGAGGACACTCTTTCCCTACACGACGCTCTTCCGATC*T	CAAGCAGAAGACGGCATACGAGATTCATCATGGTGACTGGAGTTCAGACGTGTGCTCTTCCGATC*T
D5_557	CCTTACAG			D7_557	TGACTACT	AGTAGTCA		AATGATACGGCGACCACCGAGATCTACACCCTTACAGACACTCTTTCCCTACACGACGCTCTTCCGATC*T	CAAGCAGAAGACGGCATACGAGATAGTAGTCAGTGACTGGAGTTCAGACGTGTGCTCTTCCGATC*T
D5_558	GTACATTG			D7_558	CAGAAGAT	ATCTTCTG		AATGATACGGCGACCACCGAGATCTACACGTACATTGACACTCTTTCCCTACACGACGCTCTTCCGATC*T	CAAGCAGAAGACGGCATACGAGATATCTTCTGGTGACTGGAGTTCAGACGTGTGCTCTTCCGATC*T
D5_559	GGAGACCA			D7_559	TGAGGCGC	GCGCCTCA		AATGATACGGCGACCACCGAGATCTACACGGAGACCAACACTCTTTCCCTACACGACGCTCTTCCGATC*T	CAAGCAGAAGACGGCATACGAGATGCGCCTCAGTGACTGGAGTTCAGACGTGTGCTCTTCCGATC*T
D5_560	CGAACACC			D7_560	CAGGTTCC	GGAACCTG		AATGATACGGCGACCACCGAGATCTACACCGAACACCACACTCTTTCCCTACACGACGCTCTTCCGATC*T	CAAGCAGAAGACGGCATACGAGATGGAACCTGGTGACTGGAGTTCAGACGTGTGCTCTTCCGATC*T
D5_561	GAGAACAA			D7_561	TGAACAGG	CCTGTTCA		AATGATACGGCGACCACCGAGATCTACACGAGAACAAACACTCTTTCCCTACACGACGCTCTTCCGATC*T	CAAGCAGAAGACGGCATACGAGATCCTGTTCAGTGACTGGAGTTCAGACGTGTGCTCTTCCGATC*T
D5_562	TGTGAATC			D7_562	CAGTGTGG	CCACACTG		AATGATACGGCGACCACCGAGATCTACACTGTGAATCACACTCTTTCCCTACACGACGCTCTTCCGATC*T	CAAGCAGAAGACGGCATACGAGATCCACACTGGTGACTGGAGTTCAGACGTGTGCTCTTCCGATC*T
D5_563	GGTTAAGG			D7_563	TTCCACCA	TGGTGGAA		AATGATACGGCGACCACCGAGATCTACACGGTTAAGGACACTCTTTCCCTACACGACGCTCTTCCGATC*T	CAAGCAGAAGACGGCATACGAGATTGGTGGAAGTGACTGGAGTTCAGACGTGTGCTCTTCCGATC*T
D5_564	AGACCGCA			D7_564	CCGCTGTT	AACAGCGG		AATGATACGGCGACCACCGAGATCTACACAGACCGCAACACTCTTTCCCTACACGACGCTCTTCCGATC*T	CAAGCAGAAGACGGCATACGAGATAACAGCGGGTGACTGGAGTTCAGACGTGTGCTCTTCCGATC*T
D5_565	AATACAGG			D7_565	AAGTTGGA	TCCAACTT		AATGATACGGCGACCACCGAGATCTACACAATACAGGACACTCTTTCCCTACACGACGCTCTTCCGATC*T	CAAGCAGAAGACGGCATACGAGATTCCAACTTGTGACTGGAGTTCAGACGTGTGCTCTTCCGATC*T
D5_566	TGATGGCC			D7_566	GGACAACG	CGTTGTCC		AATGATACGGCGACCACCGAGATCTACACTGATGGCCACACTCTTTCCCTACACGACGCTCTTCCGATC*T	CAAGCAGAAGACGGCATACGAGATCGTTGTCCGTGACTGGAGTTCAGACGTGTGCTCTTCCGATC*T
D5_567	TGTCACCT			D7_567	TTCGAACC	GGTTCGAA		AATGATACGGCGACCACCGAGATCTACACTGTCACCTACACTCTTTCCCTACACGACGCTCTTCCGATC*T	CAAGCAGAAGACGGCATACGAGATGGTTCGAAGTGACTGGAGTTCAGACGTGTGCTCTTCCGATC*T
D5_568	GCTTCGGC			D7_568	CAGACCAC	GTGGTCTG		AATGATACGGCGACCACCGAGATCTACACGCTTCGGCACACTCTTTCCCTACACGACGCTCTTCCGATC*T	CAAGCAGAAGACGGCATACGAGATGTGGTCTGGTGACTGGAGTTCAGACGTGTGCTCTTCCGATC*T
D5_569	CCAGTGGT			D7_569	TTCTGGTG	CACCAGAA		AATGATACGGCGACCACCGAGATCTACACCCAGTGGTACACTCTTTCCCTACACGACGCTCTTCCGATC*T	CAAGCAGAAGACGGCATACGAGATCACCAGAAGTGACTGGAGTTCAGACGTGTGCTCTTCCGATC*T
D5_570	GCACACGC			D7_570	CAATCGAA	TTCGATTG		AATGATACGGCGACCACCGAGATCTACACGCACACGCACACTCTTTCCCTACACGACGCTCTTCCGATC*T	CAAGCAGAAGACGGCATACGAGATTTCGATTGGTGACTGGAGTTCAGACGTGTGCTCTTCCGATC*T
D5_571	GTCACGTC			D7_571	AAGTACAG	CTGTACTT		AATGATACGGCGACCACCGAGATCTACACGTCACGTCACACTCTTTCCCTACACGACGCTCTTCCGATC*T	CAAGCAGAAGACGGCATACGAGATCTGTACTTGTGACTGGAGTTCAGACGTGTGCTCTTCCGATC*T
D5_572	GCAGCTCC			D7_572	CCGTGCCA	TGGCACGG		AATGATACGGCGACCACCGAGATCTACACGCAGCTCCACACTCTTTCCCTACACGACGCTCTTCCGATC*T	CAAGCAGAAGACGGCATACGAGATTGGCACGGGTGACTGGAGTTCAGACGTGTGCTCTTCCGATC*T
D5_573	CATGCAGC			D7_573	CATTGCAC	GTGCAATG		AATGATACGGCGACCACCGAGATCTACACCATGCAGCACACTCTTTCCCTACACGACGCTCTTCCGATC*T	CAAGCAGAAGACGGCATACGAGATGTGCAATGGTGACTGGAGTTCAGACGTGTGCTCTTCCGATC*T
D5_574	ACGATTGC			D7_574	TTACCTGG	CCAGGTAA		AATGATACGGCGACCACCGAGATCTACACACGATTGCACACTCTTTCCCTACACGACGCTCTTCCGATC*T	CAAGCAGAAGACGGCATACGAGATCCAGGTAAGTGACTGGAGTTCAGACGTGTGCTCTTCCGATC*T
D5_575	GACATTCG			D7_575	CTGCAACG	CGTTGCAG		AATGATACGGCGACCACCGAGATCTACACGACATTCGACACTCTTTCCCTACACGACGCTCTTCCGATC*T	CAAGCAGAAGACGGCATACGAGATCGTTGCAGGTGACTGGAGTTCAGACGTGTGCTCTTCCGATC*T
D5_576	GCGAATAC			D7_576	TACTGTTA	TAACAGTA		AATGATACGGCGACCACCGAGATCTACACGCGAATACACACTCTTTCCCTACACGACGCTCTTCCGATC*T	CAAGCAGAAGACGGCATACGAGATTAACAGTAGTGACTGGAGTTCAGACGTGTGCTCTTCCGATC*T

Supplementary Table 2:
Enzymes and cycling conditions used in this study

Phase 1 PCR Enzymes and conditions

Enzyme	Company	Format	Cycling conditions				
			Initial Denaturation	Denaturation	Annealing	Extension	Polishing
Simplifi	Bioline	2x	95C - 30 sec	95C - 15 sec	60C - 15 sec	72C -60 sec	72C -5 min
Verifi HS	PCRBio	2x	95C - 60 sec	95C - 15 sec	60C - 15 sec	72C -60 sec	72C -5 min
HiFi mastermix	Youseq	2x	95C - 2 min	95C - 20 sec	60C - 15 sec	72C -60 sec	72C -5 min
iProof	BioRad	2x	98C - 2 min	98C - 10 sec	60C - 15 sec	72C -60 sec	72C -5 min
Allin HiFi	HighQu	2x	95C - 60 sec	95C - 15 sec	60C - 15 sec	72C -60 sec	72C -5 min
repliQa Toughmix HiFi	Quantabio	2x	98C - 30 sec	98C - 10 sec	60C - 15 sec	68C -60 sec	68C -60 sec
Primestar Max	Takara	2x		98C - 10 sec	60C - 15 sec	72C -60 sec	72C -60 sec
AQ97	Ampliquon	2x	98C - 2 min	98C - 20 sec	60C - 15 sec	72C -60 sec	72C -5 min
SparQ	Quantabio	2x	98C - 2 min	98C - 20 sec	60C - 15 sec	72C -60 sec	72C -60 sec
HiFi	Kapa	2x	98C - 2 min	98C - 20 sec	60C - 15 sec	72C -60 sec	72C -2 min
Platinum superfi Ii	Invitrogen	2x	98C - 30 sec	98C - 10 sec	60C - 15 sec	72C -60 sec	72C -60 sec
Collibri Mastermix	Invitrogen	2x	98C - 30 sec	98C - 15 sec	60C - 15 sec	72C -60 sec	72C -60 sec
Veraseq 2.0	Enzymatics	Enzyme + buffer	98C - 30 sec	98C - 10 sec	60C - 15 sec	72C -60 sec	72C -60 sec
UCP HiFidelity PCR mix	QIAGEN	2x	98C - 30 sec	98C - 10 sec	60C - 15 sec	72C -60 sec	72C -60 sec
Phusion U multiplex PCR mastermix	Thermo	2x	98C - 30 sec	98C - 10 sec	60C - 15 sec	72C -60 sec	72C -5 min
Ultra hiFi	Tools Biotech	2x	95C - 5 min	95C - 20 sec	60C - 15 sec	72C -60 sec	72C -5 min
Universe	Biotool	2x	95C - 60 sec	95C - 15 sec	60C - 15 sec	72C -60 sec	72C -5 min
Herculase II	Agilent	Enzyme + buffer	98C - 2 min	98C - 20 sec	60C - 15 sec	72C -60 sec	72C -5 min
PfuUltra™ II Fusion HS DNA Polymerase	Agilent	Enzyme + buffer	95C - 2 min	95C - 20 sec	60C - 15 sec	72C -60 sec	72C -5 min
HiFi	Kapa	2x	95C - 2 min	95C - 30 sec	60C - 15 sec	72C -60 sec	72C -2 min
HiFi	Kapa	2x	94C - 2 min	94C - 15 sec	60C - 15 sec	72C -60 sec	72C -2 min
Q5	NEB	2x	98C - 30 sec	98C - 10 sec	60C - 15 sec	72C -60 sec	72C -60 sec
HiFi	Cole Palmer	2X		98 - 30 sec	60C - 15 sec	72C -60 sec	72C -60 sec

Phase 2 PCR Enzymes

Enzyme	Company	Format	Cycling conditions used	Comment
repliQa Toughmix HiFi	Quantabio	2x	A (plus E,F for plasmodium)	
HiFi	Kapa	2x	B, C, D, E	
Collibri Mastermix	Invitrogen	2x	B, C, D, E	
Herculase II	Agilent	Enzyme + buffer	B, C, D, E	
Equinox	Watchmaker	2x	C, D, E, F, K	
Q5	NEB	2x	G, H, I, J	
Ex Premier	Takara	2x	L	

Phase 2 PCR cycling Conditions

	Cycling conditions				
	Initial Denaturation	Denaturation	Annealing	Extension	Polishing
A	98C - 30 sec	98C - 10 sec	60C - 15 sec	68C -60 sec	68C -60 sec
B	98C - 2 min	98C - 20 sec	60C - 30 sec	72C -60 sec	72C -2 min
C	95C - 2 min	95C - 30 sec	60C - 30 sec	72C -60 sec	72C -2 min
D	94C - 2 min	94C - 15 sec	60C - 30 sec	72C -60 sec	72C -2 min
E	95C - 3 min	98C - 20 sec	60C - 30 sec	72C -60 sec	72C -2 min
F	98C - 30 sec	98C - 10 sec	60C - 15 sec	72C -60 sec	72C -60 sec
G	98C - 30 sec	98C - 10 sec	60C - 30 sec	65C - 75 sec	65C - 5 min
H	95C - 3 min	98C - 10 sec	65C - 75 sec		65C - 5 min
I	95C - 3 min	98C - 10 sec	65C - 75 sec		65C - 5 min
J	94C - 3 min	94C - 30 sec	65C - 75 sec		65C - 5 min
K	98C - 45 sec	98C - 10 sec	60C - 15 sec	72C -60 sec	72C -60 sec
L	94C - 1 min	98C - 10 sec	60C - 15 sec	68C -30 sec	68C -60 sec

Long range PCR cycling Conditions

		Cycling conditions				
**Company**	**Enzyme**	**Denature**	**Denature**	**Anneal**	**Extend**	**Polish**
promega	go taq long	94-2min	94-30s	60-15sec	72-20min	72-10min
Takara	primestar GXL	98-30s	98-10s	60-15sec	72-20min	72-1min
Takara	Terra	98-2min	98-10sec	60-15sec	68-20min	68-1min
Roche	Kapa long	94-3min	94-25sec	60-15sec	68-20min	68-1min
Roche	kapa HiFi	98-2min	98-20s	60-15sec	72- 20min	72-5min
NEB	long amp	94-30s	94-30sec	60-15sec	65-18min	65-10min
Takara	LA taq	94-1min	98-10sec	60-15sec	68-20min	72-10min
Invitrogen	Platinum SuperFi II	98-30s	98-10sec	60-15sec	72-20min	72-5min
Roche	Expand	94-2min	94-10sec	60-15sec	68-20min	68-7min
Qiagen	Ultrarun	93-3 min	93-30s	60-15sec	65s-10min	72-10min
PCRbio	Verifi	95-1min	95-15sec	60-15sec	72-20min	72-5min
Agilent	PfuUltra™ II Fusion H	95-2min	95-30s	60-30s	72-20min	72-5min
Quantabio	repliQa	98-30sec	98-10s	60-15sec	68-10min	68-1min
watchmaker	Equinox	98-45s	98-15s	60-30s	72 - 10 min	72 1min
Universe	Biotool	95-1min	95-15sec	60-15sec	72-20min	72-5min
NEB	Q5	98-30SEC	98-10S	60-15sec	65-20 min	65-5 min

Supplementary Table 3:

PCR Yields from long range PCR with different enzymes after 15 cycles of PCR using 1ng S. cerevisiae genomic DNA template

* A) ELF fractionated template, B) Bluepippin fractionated template

Enzyme	Template*	DNA Yield (ng/ul)	Total Yield (ng)
Terra	A	0.952	28.55
Terra	B	5.895	176.86
repliQa	A	5.351	160.53
repliQa	B	10.629	318.88
LongAmp	A	5.061	151.82
LongAmp	B	1.120	33.6
Q5	A	0.765	22.94
Q5	B	0.973	29.19
wm 1x	A	0.739	22.18
wm 1x	B	1.143	34.28
wm 0.8x	A	0.842	25.27
wm 0.8x	B	0.853	25.58
promega	A	9.635	289.04
promega	B	16.926	507.77
LaTaq	A	6.651	199.54
LaTaq	B	1.261	37.83
Extend	A	0.856	25.67
Extend	B	0.443	13.3
Universe	A	0.611	18.34
Universe	B	1.128	33.83
Superfill	A	1.288	38.65
Superfill	B	2.371	71.14
verifi 2x	A	1.280	38.4
verifi 2x	B	0.976	29.28
Pfu ultra II	A	0.673	20.19
Pfu ultra II	B	0.378	11.34
Primestar MAX	A	0.758	22.73
Primestar MAX	B	1.276	38.27
PrimestarGXL	A	1.188	35.64
PrimestarGXL	B	1.100	33.01
Takara Ex Premier	B	0.590	17.69
